# Constructing and Validating a Pyroptosis-Related Genes Prognostic Signature for Stomach Adenocarcinoma and Immune Infiltration: Potential Biomarkers for Predicting the Overall Survival

**DOI:** 10.1155/2022/3102743

**Published:** 2022-09-26

**Authors:** Jingmin Xu, Ke Chen, Zhou Wei, Zixuan Wu, Xuyan Huang, Minjie Cai, Kai Yuan, Peidong Huang, Jing Zhang, Shuai Wang

**Affiliations:** ^1^Yantai Hospital of Traditional Chinese Medicine, Shandong Province, China; ^2^Department of Clinical Laboratory, The Sixth Affiliated Hospital of Sun Yat-Sen University, Guangzhou, 510655 Guangdong, China; ^3^Guangzhou University of Chinese Medicine, Guangzhou, Guangdong Province, China 510006; ^4^Shantou Health School, Shantou, Guangdong Province, China 515061; ^5^Yunnan University of Chinese Medicine, Kunming, Yunnan Province, China 650500; ^6^Department of Pediatrics, Shandong Second Provincial General Hospital, Shandong, China; ^7^Department of Pediatric Surgery, Shandong Provincial Hospital Affiliated to Shandong First Medical University, China

## Abstract

**Background:**

Stomach adenocarcinoma (STAD) is a kind of cancer that begins in the stomach cells and has a poor overall survival rate. Following resection surgery, chemotherapy has been suggested as a curative method for stomach cancer. However, it is ineffective. Pyroptosis, a kind of inflammatory programmed cell death, has been shown to play a significant role in the development and progression of STAD. However, whether pyroptosis-related genes (PRGs) can be utilized to predict the diagnosis and prognosis of gastric cancer remains unknown.

**Method:**

The research measured at predictive PRGs in STAD samples from TCGA and GEO. Lasso regression was used to build the prediction model. Coexpression analysis revealed that gene expression was linked to pyroptosis. PRGs were found to be overexpressed in high-risk individuals, implying that they could be used in a model to predict STAD prognosis.

**Result:**

Immunological and tumor-related pathways were discovered using GSEA. In STAD patients, the genes *GPX3*, *PDGFRL*, *RGS2*, and *SERPINE1* may be connected to the cancer process. The levels of expression also differed between the two risk groups.

**Conclusion:**

The purpose of this study is to identify and verify STAD-associated PRGs that can effectively guide prognosis and the immunological milieu in STAD patients as well as offer evidence for the development of pyroptosis-related molecularly targeted therapeutics. Therefore, PRGs and the link between immunological and PRGs in STAD may be therapeutic targets.

## 1. Introduction

Gastric cancer (GC), a disease with a wide range of manifestations, is the fifth most frequent cancer and the third biggest cause of cancer-related deaths globally. The most frequent histologic form of gastric cancer, stomach adenocarcinoma (STAD), is a fast developing, aggressive, and malignant GC that accounts for 95 percent of all gastric tumors. Several previous studies have found that Helicobacter pylori infection causes 90% of STAD cases [1]. Many researchers have recently proposed that STAD could also be brought about by autoimmunity, other bacteria, and their metabolites (such as N-nitroso compounds or acetaldehyde) [2]. STAD research has advanced to the point where it may be regarded as a collection of uncommon illnesses that risk human health [3]. Currently, the treatment of this disease is as important in the field of tumor research [4]. Chemotherapy is a significant factor of tumor treatment, but because chemotherapy drugs are cytotoxic and seem to have a lot of side effects, long-term use will cause major problems for patients. Repeated use can easily result in tumor cell drug resistance, reducing the curative effect [5]. Despite this, the absence of precise biomarkers for early tumor diagnosis, as well as restricted preclinical models, has impeded successful STAD therapeutic treatment [6, 7]. As a result, there is an urgent need to identify novel and reliable biomarkers for the early identification and prognosis of STAD. Finding treatment targets for STAD and elucidating the molecular identification of diagnostic biomarkers are critical for basic and clinical STAD research.

One of life's most fundamental challenges is cell death. The capacity to avoid cell death, which is a characteristic of cancer, not only contributes to the formation of cancer but also plays a significant role in the development of therapeutic resistance, recurrence, and metastasis [8]. The ultimate objective of cancer therapies like radiation, chemotherapy, and immunotherapy, which has recently made great progress, is to maximize tumor cell death while causing the least amount of injury to normal tissues. Tumor cells' innate genetic and epigenetic heterogeneity, as well as metabolic flexibility and other variables, provide greater adaptability to adverse tumor settings, resulting in treatment resistance and spread potential [9]. Pyroptosis is a double-edged sword that plays a twofold role in modifying tumor growth due to the ongoing activation of the inflammasome. Pyroptosis aids in the formation of a tumor-suppressive immunological milieu by unleashing inflammatory chemicals capable of directly destroying cancer cells and galvanizing an anticancer immune response [10]. Pyroptosis, a highly immunogenic form of cell death, induces local inflammation and draws inflammatory cell infiltration, offering a good chance to reduce immunosuppression of tumor microenvironments (TME) and stimulate a systemic immune response in the treatment of solid tumors [11]. In rare cases, triggering pyroptosis can directly kill tumor cells. According to new study, pyroptosis has a role in cancer formation, differentiation, invasion, and late metastasis as well as tumor sensitivity to immune medication therapy [12]. Pyroptosis-related chemicals have a crucial oncogenic function in the development of gastric cancer.

Immune checkpoint inhibitor (ICI) profiles in STAD patients may aid in diagnosing, analyzing, and anticipating therapy results [13]. The reason and methods of STAD's aberrant gene expression and pyroptosis remain unclear at this time. Understanding how PRGs regulate STAD production might result in the development of an indicator that can be employed as a therapeutic strategy.

## 2. Materials and Methods

We used the approaches proposed by Zi-Xuan Wu, et al. 2021 [14].

### 2.1. Datasets and PRGs

The Cancer Genome Atlas was used to collect STAD gene expression patterns and clinical data (TCGA) [15]. 375 STADs and 32 normal data were registered in the TCGA on May 6, 2022. The Gene Expression Omnibus (GEO) was searched for mRNA expression on May 6, 2022. Series: GSE84437. Platform: GPL6947-13512. The GEO was used to maintain 433 STAD cases [16](Table 1). We also identified 52 PRGs in total [17] (Table S1).

### 2.2. DEGs Linked to Pyroptosis and Mutation Rates

Perl matched and sorted transcription data and human configuration files to acquire exact mRNA data. The gene IDs were converted into gene names using information from the ensemble database. The R Limma was utilized to get the expression data for the PRGs. FDR < 0.05 and |log2FC| ≥ 1 were used to evaluate if there was a significant change in PRG expression [18]. The role of differentially expressed PRGs that were both up- and down-regulated was investigated (DEGs). We also explored the genetic alterations in these genes. Cbioportal was used to estimate DEG mutation frequencies.

### 2.3. Tumor Classification Based on the DEGs

First, we used the Limma and ConsensusClusterPlus package to do cluster analysis, and we separated the prognosis-related PRGs into two clusters: cluster 1 and 2. Survminer was being used to study the survival of PRG subgroups, and survival was used to evaluate PRG's predictive validity. The pheat map was used to generate a heat map of the differential gene expression of prognosis-related PRGs, and the relationship between PRGs and clinicopathological features was explored. The limma was used to identify differences in target gene expression across categories. To study the gene interaction between STAD target genes and prognostic PRGs, the limma and corrplot programs were utilized.

### 2.4. Development of PRGs Prognostic Signature

Every STAD patient's risk score was also evaluated. The DEGs were divided into two groups based on their support for the median score: low-risk and high-risk. Lasso regression was shown to be associated with two risk classifications. The boldness interval and risk ratio were estimated after seeing the image, and the forest diagram was created as a consequence. Survival curves for the two groups were developed and compared. To test the model's accuracy in predicting survival in STAD, the timeROC was used to generate a comparable receiver-operating characteristics (ROC) curve. The risk and survival status of PRGs were explored using the risk score's probability curve. The link between two PRGs patients was established, as was the relationship between clinical characteristics and the risk prediction model. Risk and clinical association analyses were distributed. T-distributed Neighbor Embedding (T-SNE) and Principal Component Analysis (PCA) were also examined. To determine if the prognostic model correctly classified patients into two risk groups, a representation was constructed to predict the 1-, 3-, and 5-year OS of STAD patients by the desegregation of prognosticative signals.

### 2.5. Functional Enrichment

The associated biological pathways were then examined using Gene Ontology (GO). BP, MF, and CC are controlled by differentially expressed PRGs. PRGs were further investigated using R based on KEGG dataset [19]. Filterpvalue < 0.05 was used to evaluate if there was a significant change in GO and KEGG.

### 2.6. GSEA Enrichment Analyses

GSEA was used to find related functions and route alterations in a variety of samples, while Perl was used to input data. The accompanying score and graphs were used to assess whether the activities and routes within the different risk categories were dynamic or not. Each sample was assigned a ‘H' or ‘L' label based on whether it included a high-risk cluster of prognosis-related PRGs.

### 2.7. Comparison of the Immune Activity

We examined the enriched score of immune cells and activities in two risk groups using the ssGSEA in both the TCGA and GEO cohorts. We also explored the connection between PRGs, checkpoints, and m^6^a.

## 3. Results

### 3.1. PRGs That Differ in Expression

**Figure 1:**
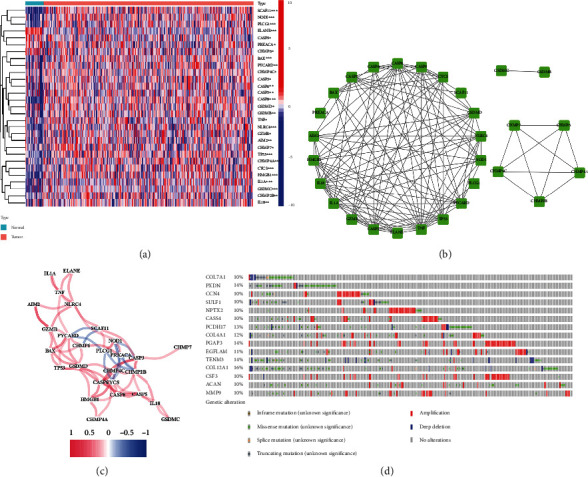
PRGs' expressions and interactions. (a) Heat map. (b) PPI network. (c) Correlation network. (d) Mutations.

Twenty-nine DEGs have been linked to pyroptosis (23 upregulated, 6 downregulated; Table S2). (Figure 1(a)). We conducted a protein-protein interaction (PPI) research, the results of which are given in Figure 1(b). By setting the minimum required interaction value to 0.4, we identified that *TNF*, *CASP8*, *IL18*, *CASP3*, *IL1A*, *CASP9*, *PYCARD*, *HMGB1*, *GSDMD*, and *TP53* were hub genes (Table S3). These genes might be utilized to create independent STAD prognostic indicators. The correlation network, seen in Figure 1(c), is made up. We discovered that the gene mutations were truncating and missense variants (Figure 1(d)). 15 genes had a 10% mutation rate, with *COL12A1* being the commonly altered (16%).

### 3.2. Drug Prediction Models and Sensitivity Analysis

**Figure 2:**
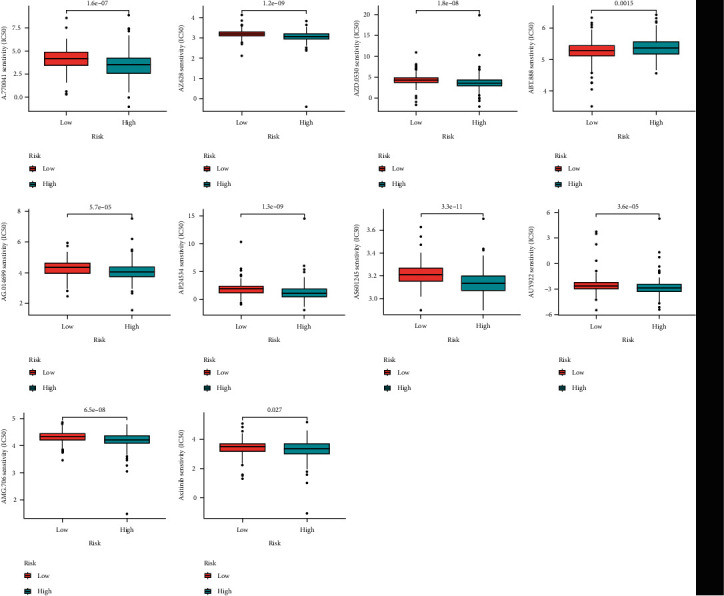
Drug prediction models.

**Figure 3:**
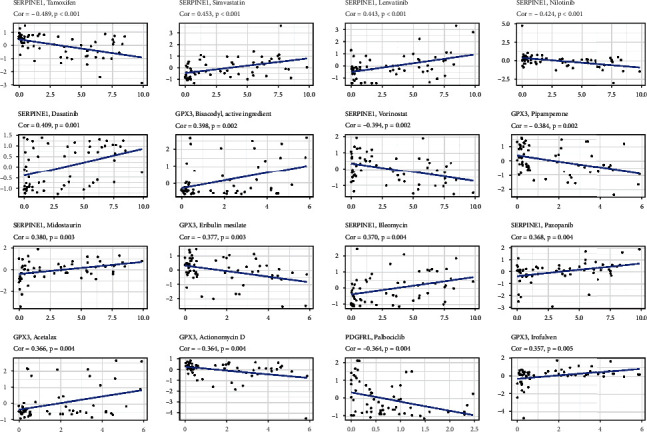
Drug sensitivity analysis.

The drug prediction of the model showed that there were some genes with significant differences (Figure 2). Furthermore, the association analysis between DEG expression in the prognostic model revealed that several genes were substantially linked with medication sensitivity. For example, there was a strong association between SERPINE1 expression and Tamoxifen, Simvastatin, Lenvatinib, Nilotinib, Dasatinib, Vorinostat, Midostaurin, Bleomycin, and Pazopanib. These findings suggest possible future medication development paths (Figure 3).

### 3.3. Tumor Classification

**Figure 4:**
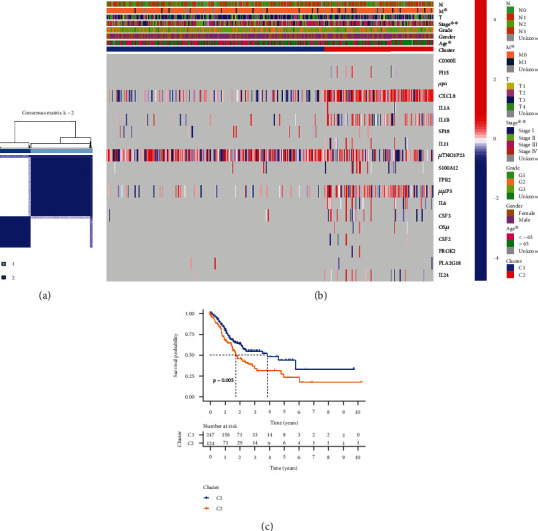
Tumor categorization. (a) Consensus clustering matrix. (b) Heat map. (c) Kaplan–Meier OS curves.

In TCGA cohort, we conducted a consensus clustering analysis on 375 STAD sufferers to investigate the relationships between PRGs and STAD subgroups. The intragroup correlations were highest and the intergroup correlations were weakest when the clustering variable (*k*) was adjusted to 2. (Figure 4(a)). A heat map reflects both the gene expression patterns and clinical characteristics (Figure 4(b), Table S4). PRG subgroups were used in a survival study to explore the predictive capacity of PRGs, and cluster 1 had a higher survival rate (*P* = 0.005; Figure 4(c)), as shown in Figure 4(c).

### 3.4. In the TCGA Cohort, a Prognostic Gene Model Was Developed

**Figure 5:**
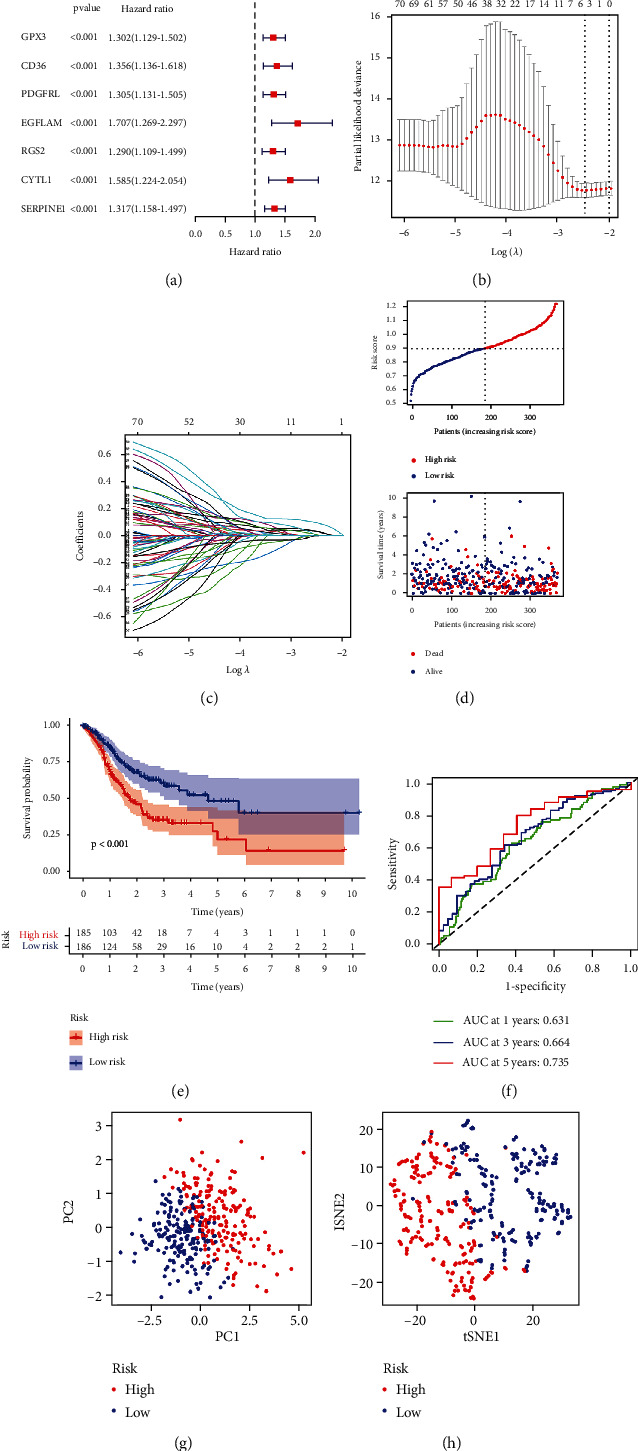
In the TCGA cohort, the formation of a risk signature. (a) Univariate cox regression analysis. (b) LASSO regression. (c) Cross-validation. (d) Survival status. (e) Kaplan–Meier curve. (f) The AUC of the survival rate. (g) PCA plot. (h) t-SNE plot.

Seven important PRGs were found throughout the COX investigation. These PRGs (GPX3, CD36, PDGFRL, EGFLAM, RGS2, CYTL1, and SERPINE1) were found as independent STAD prognostic markers (Figure 5(a)). The most minor absolute shrinkage and choice operator Cox regression analysis (LASSO) and the optimal value were used to build a gene signature (Figures 5(b) and 5(c)). We observed that a patient's risk score was negatively connected to STAD patients' survival using a risk survival standing plot. The presence of high-risk PRG signatures was linked to a reduced chance of survival (*P* < 0.001, Figure 5(e)). For 1-, 3-, and 5-year survival rates, the AUC of the unique PRGs signature was 0.631, 0.664, and 0.735, respectively (Figure 5(f)). Our data analysis revealed that the great majority of STAD patients lived for less than 5 years, therefore the AUC of less than 0.6 in the fifth year is a result of this. PCA and t-SNE results indicated that patients with varying risks were divided into two groups (Figures 5(g) and 5(h)).

### 3.5. The Risk Signature Is Externally Validated

**Figure 6:**
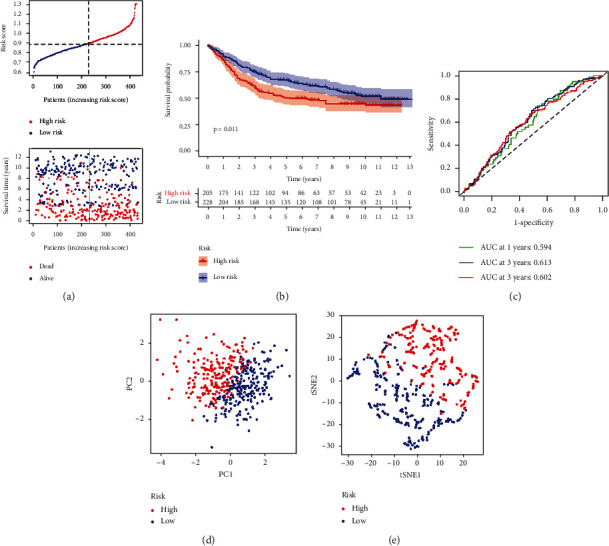
In the GEO cohort, the risk model was verified. (a) Survival status. (b) Kaplan–Meier curve. (c) The AUC of the survival rate. (d) PCA plot. (e) t-SNE plot.

We observed that a patient's risk score was adversely related to STAD patient survival. Surprisingly, majority of the novel PRGs discovered during this research were adversely related with our risk model, comparable to the TCGA findings (Figure 6(a)). High-risk PRG signatures were related with a lower likelihood of survival (*P* = 0.011, Figure 6(b)). The AUC of the distinctive PRGs signature was 0.594, 0.613, and 0.602 for 1-, 3-, and 5-year survival rates, respectively (Figures 6(c)). Our data analysis found that the vast majority of STAD patients lived for more than 1 years, resulting in an AUC of less than 0.7 in the fifth year. The PCA and t-SNE findings showed that patients with variable risks were efficiently sorted into two different groups (Figures 6(d) and 6(e)). We also created a heat map (Figure 6(e)).

### 3.6. Independent Prognostic Value of the Risk Model

**Figure 7:**
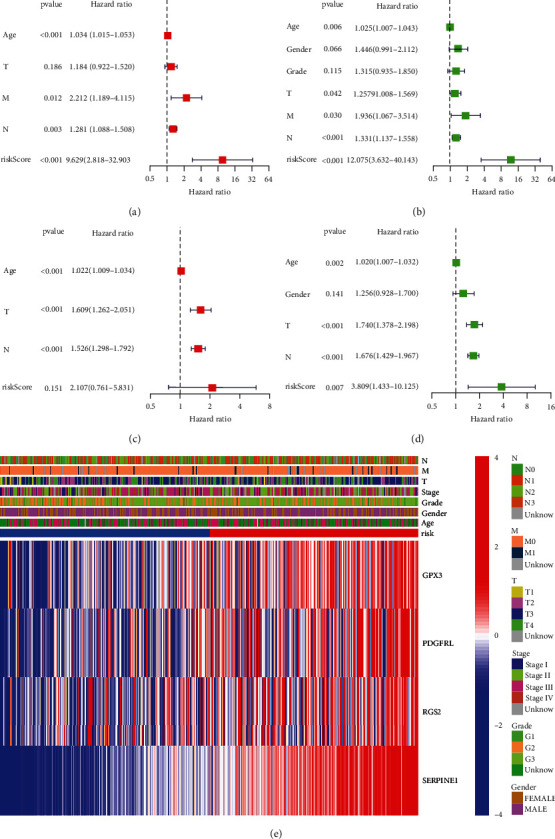
Cox regression analysis. (a,b) TCGA cohort. (c,d) GEO cohort. (a,c) Multivariate analysis. (b,d) Univariate analysis. (e) Heat map highlighting the relationships between clinicopathological characteristics and risk categories.

In TCGA cohort, COX analysis demonstrated that the PRGs signature (HR: 9.629, 95CI: 2.818-32.903), Age (HR: 1.034, 95CI: 1.015-1.053), M stage (HR: 2.212, 95CI: 1.189-4.115), and N stage (HR: 1.281, 95CI: 1.088-1.508) were primarily independent predictive variables for the OS of STAD patients (Figures 7(a) and 7(b)). In GEO cohort, COX analysis demonstrated that Age (HR: 1.022, 95CI: 1.009-1.034), T stage (HR: 1.609, 95CI: 1.262-2.051), and N stage (HR: 1.526, 95CI: 1.298-1.792) were primarily independent predictive variables for the OS of STAD patients (Figures 7(c) and 7(d)). In addition, for the TCGA cohort, we constructed a heat map of clinical characteristics (Figure 7(e)) (Table S5-6).

### 3.7. Enrichment Analysis of Pyroptosis-Related Genes

**Figure 8:**
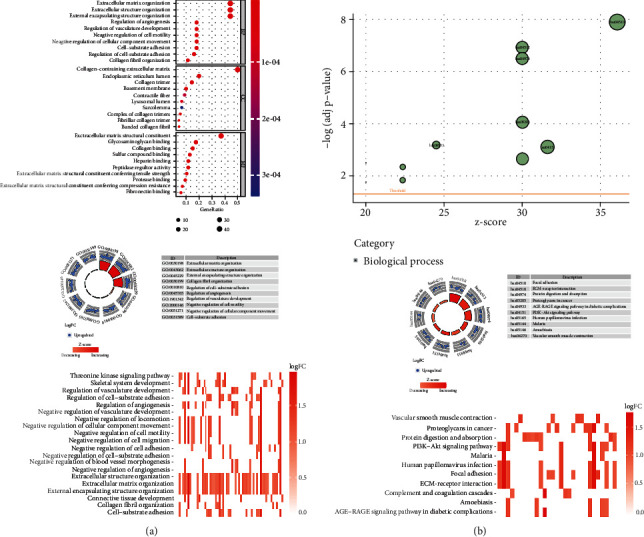
Enrichment analysis for PRGs. (a) GO and (b) KEGG.

GO enrichment analysis revealed 550 core targets, including Biological processes (BP), molecular functions (MF), and cellular components (CC). The MF mainly involves actin–binding (GO:0003779) and enzyme inhibitor activity (GO:0004857). The CC mainly involves focal adhesion (GO:0005925) and cell leading edge (GO:0031252). The BP mainly involves skeletal system development (GO:0001501), cell growth (GO:0016049), and negative regulation of hydrolase activity (GO:0051346). In addition, the main signaling pathways were identified by KEGG enrichment analysis, it revealed the over-expressed genes were mainly involved in PI3K-Akt signaling pathway (hsa04151), Proteoglycans in cancer (hsa05205), Focal adhesion (hsa04510), Vascular smooth muscle contraction (hsa04270), Protein digestion and absorption (hsa04974), and Amoebiasis (hsa05146) (Figure 8 and Table S7a-b).

### 3.8. Analyses of GSEA

**Figure 9:**
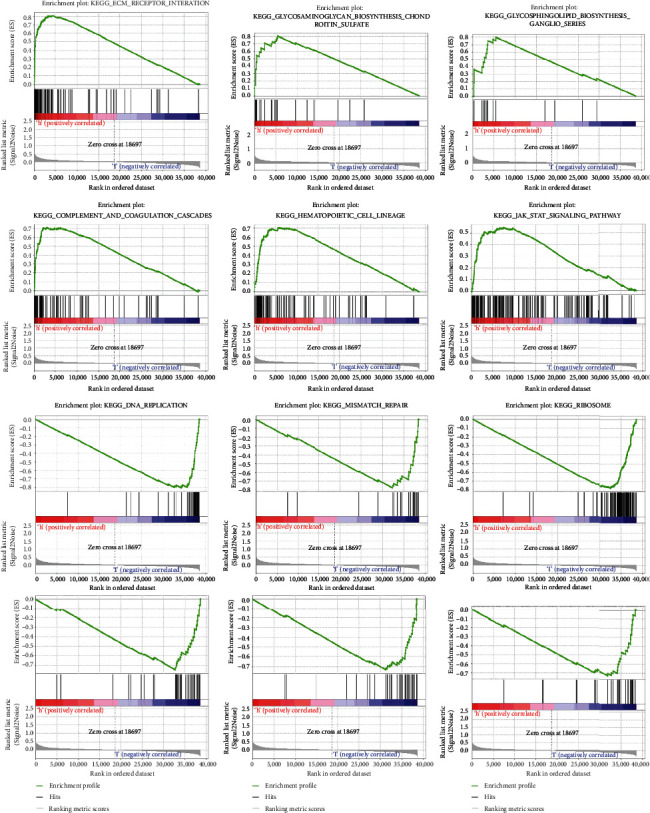
GSEA analyses.

According to GSEA, the majority of PRG prognostic signatures controlled immunological and tumor-related pathways—ecm receptor interaction, complement and coagulation cascades, hedgehog, tgf beta, jak stat, and chemokine signaling pathway, etc. Each cluster's top six enriched functions or pathways are displayed (Figure 9, Table 2). The “hedgehog signaling pathway” was the most enriched, and some of the genes were shown to be positively associated to H or L. (Table S8a-b).

### 3.9. Immune Activity Comparisons

**Figure 10:**
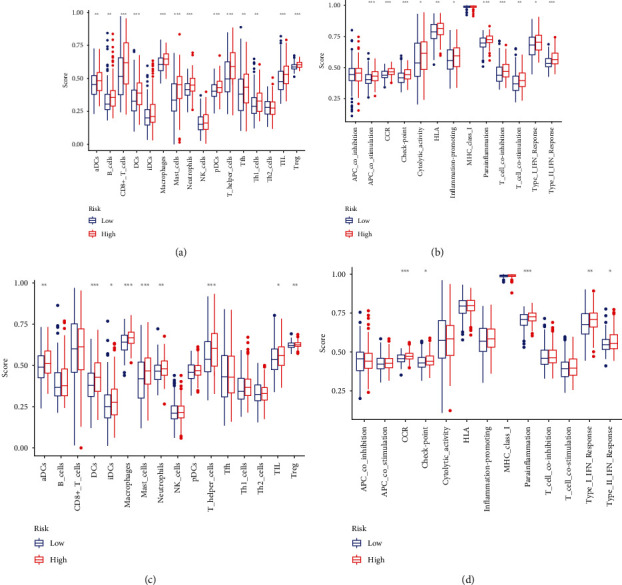
The ssGSEA scores for immune cells and immune function. (a,b) TCGA cohort. (c,d) GEO cohort.

We investigated the enrichment scores of 16 kinds of immune cells and the activity of 13 immune-related activities in both the TCGA and GEO cohorts using the single-sample gene set enrichment approach (ssGSEA). In the TCGA cohort, IDCs, NK cells, and Th2 cells did not differ significantly between the two groups (*P* > 0.05). Other immune cells generally show higher levels of infiltration in the high-risk grouping (Figure 10(a)). APC coinhibition and MHC class I did not differ significantly between the two groups (*P* > 0.05). Other immune-related function generally show higher levels in the high-risk grouping (Figure 10(b)). When assessing the immune status in the GEO cohort, similar conclusions were drawn (Figures 10(c) and 10(d)).

### 3.10. An Examination of the Relationship between PRGs and Immunological Checkpoints and m^6^a

**Figure 11:**
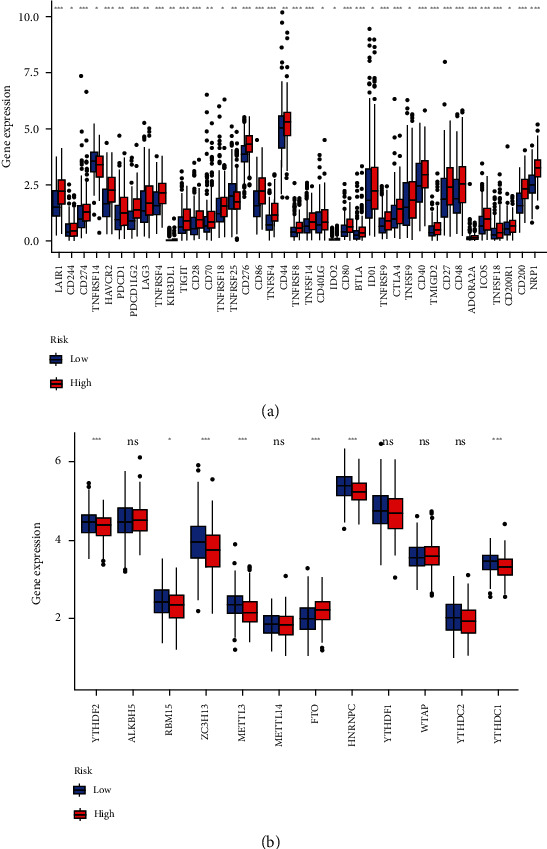
(a) Expression of immune checkpoints and (b) the expression of m^6^a-related genes.

*LAIR1*, *CD274*, *HAVCR2*, *PDCD1LG2*, *TNFRSF4*, and other genes were expressed differently (Figure 11(a)). When PRG expression levels were compared between the two-risk groups, *FTO* was significantly higher in the high-risk group. While *YTHDF2*, *RBM15*, *ZC3H13*, *METTL3*, *HNRNPC*, and *YTHDC1* were shown to be much more significant in the low-risk group (Figure 11(b)). The expression of *FTO* associated with m^6^a modification was higher in the high risk group, indicating that it may be linked to the malignancy activity in STAD sufferers. While *YTHDF2*, *RBM15*, *ZC3H13*, *METTL3*, *HNRNPC*, *YTHDC1* with m^6^a modifications had higher expression in the low risk group, indicating that they might be tumor suppressors.

## 4. Discussion

Treating STAD is a severe clinical issue because of its advanced stage and terrible prognosis. The current state of precision medicine for STAD is limited by a scarcity of powerful tumor-killing initiators and selective tumor-targeting therapeutic agents. Recent study has shown that the focused therapeutic impact of STAD may be successfully increased by modifying the process of programmed tumor cell death [20]. Pyroptosis, a recently identified process of programmed cell death, is gaining prominence in the context of innate immunity, carcinogenesis, and patient responses to anticancer therapy [21, 22]. Pyroptosis occurs in pathogen-infected cells, causing an inflammatory reaction and cell lysis within the host body [23]. Pyroptosis manifests itself in malignancies in two ways. On the one hand, the inflammasome can efficiently promote tumor cell death by activating the pyroptosis pathway, therefore reducing tumor cell growth and invasion [24]. It is unknown how it impacts STAD development by modifying PRGs. We studied the function of critical proteins and processes in STAD prognosis and established a suitable biomarker and anticancer activity.

In a university Cox regression investigation, PRGs were found to be strongly linked with STAD prognosis. The researchers discovered four prognostic PRGs that have been expressed differently in two-risk persons. Some PRGs were identified to be highly expressed in high-risk, whereas others were seen to be differentially expressed in low-risk (*P* < 0.05). A survival analysis was used to find the prognostic capacity of PRGs after additional examination into their influence. Individuals with STAD who had low-risk PRGs survived longer. The markers *GPX3*, *PDGFRL*, *RGS2*, and *SERPINE1* were found to be significantly increased in the high-risk group, suggesting that all of these markers may be implicated in the malignancy processes for STAD patients and may be cancer-promoting factors. The findings of the above-mentioned biomarker suggest some suggestions for future work, but concrete evidence that they will be responsible for the synthesis of important transcription factors associated with pyroptosis regulation, such as *PD-L1*, *GSDMB*, and *ROS-NLRP3* [25–27], is lacking, necessitating further exploration.

Compared to normal tissues and cells, Gpx3 expression was lower in gastric cancer (GC) patients and GC cell lines. Cai et al. believes that Gpx3 inhibits gastric cancer migration and invasion by targeting NFкB/Wnt5a/JNK signaling [28]. When GPX3 expression in breast cancer cells and tissues was compared to normal controls, it was shown to be low. GPX3 overexpression inhibited breast cancer growth, colony formation, migration, and invasion in vitro. Furthermore, hypermethylation of the GPX3 promoter and suppression of hsa-miR-324-5p release have been identified as probable pathways for GPX3 downregulation in breast cancer [29]. Through bioinformatics analysis, Huo et al. discovered PDGFRL was one of the tumor-associated macrophages (TAMs). It is vital in the progression of malignant tumors and performed well in predicting overall survival (OS) in GC [30]. Cancer cell dormancy and tumor relapse are mediated by RGS2-mediated translational control [31]. SERPINE1 was found to be significantly upregulated in gastric tissues and associated with poor outcomes in a genome-scale analysis. As a result, Liao et al. thought of SERPINE1 as a diagnostic and prognostic biomarker in GC [32]. Because these PRGs appear to be associated with cancer processes in STAD patients, these studies highlight the validity and plausibility of our findings. According to the OS and ROC analyses of the GSE84437 KM-curves, a PRGs-signature might be employed as a viable predictive predictor. Only a few investigations on the gene alterations associated with pyroptosis have been conducted. More research is needed to fully understand the mechanics of PRG alteration and classification, as well as to validate our findings.

KEGG analysis found that the genes were primarily involved in PI3K-Akt signaling pathway. DHA protects against hepatic ischemia reperfusion injury by inhibiting pyrolysis and activating the PI3K/Akt signaling pathway [33]. Pioglitazone Provides Neuroprotection Against Ischemia-Induced Pyroptosis by inhibiting the RAGE pathway by Activating PPAR-ɤ [34]. Consequently, Pyroptosis is crucial in STAD. In GSEA, the hedgehog signaling pathway was found to be the most significantly enriched pathway. Smo and Gli1 genes are components of the hedgehog signaling pathway, and their over expression can cause STAD. The degree of expression is linked to the stage and severity of STAD [35]. Furthermore, studies have shown that Hedgehog-interactingprotein (HHIP) may inhibit the growth and proliferation of STAD cell lines by blocking Hedgehog signal transduction, which may become a new biological marker for STAD and a new approach for STAD treatment by targeting the drug target of HHIP formation [36]. Overactivation of the hedgehog pathway is linked to the occurrence and progression of STAD, and specific targeted therapy targeting this pathway may become an effective new measure for clinical treatment of STAD [37]. Taking the aforementioned properties into account, PRGs may influence STAD cell migration and proliferation via modulating the nod like receptor signaling pathway.

Furthermore, our technique accurately predicts STAD patients' survival. Increases in risk score are linked to higher death rates and a higher high-risk ratio. Based on our findings and data from the literature, PRGs appear to be significant biomarkers for predicting STAD patient outcomes. Recent research has discovered a link between several cell death mechanisms and anticancer immunity. Even in ICI-resistant tumors, pyroptosis, ferroptosis, and necroptosis activation in conjunction with ICIs resulted in synergistically improved anticancer efficacy [38, 39]. De novo pyroptosis in ICI-resistant cancers can produce an inflammatory milieu that mediates tumor susceptibility to immune checkpoint inhibitors (ICI), promoting pyroptosis and inhibiting tumor growth in autochthonous tumors [40]. Despite the fact that there has been minimal study on PRGs and STAD, based on the evidence presented above, it is reasonable to believe that PRG changes were associated with the onset and development of STAD.

There have been a number of publications published in recent years that examine the association between pyroptosis and STAD [41, 42]. However, when compared to other studies, the approach used in this study is novel. To begin, PRGs in the TCGA database are routinely updated. We have made further additions to previous articles. Second, TCGA data were used as the primary analysis, with GEO data included into the common pattern for model validation. Third, GO and KEGG analyses, as well as a GSEA analysis, were performed. The conclusions of the two investigations coincided, which increased trust. Fourth, to increase the trustworthiness of the results, we employed different databases to quantify immune cells and functions.

Our analysis has the following limitations: (1) we will be unable to obtain sufficient different data sources from other publically available sites to validate the model's trustworthiness. (2) We investigated the functional enrichment processes engaged in the regulatory networks of distinct risk groups; however, their particular mechanisms in enabling pyroptosis require more exploration to corroborate our findings. (3) Although the model was validated in the GEO dataset, the prediction model developed in this work still has to be externally and practically verified before it can be used on clinical patients.

## 5. Conclusions

In STAD sufferers, 4 expected PRGs were discovered. The findings contribute to a better understanding of the immunological system's role in pyroptosis, perhaps paving the way for new effective treatments and prognostic biomarkers. Pyroptosis regulation may be a promising therapeutic technique for improving the result of STAD immunotherapy and providing a tailored prognostic tool for prognosis and immune response.

## Figures and Tables

**Table 1:** Patients' clinical features.

Variable	Number of samples
*TCGA*	
Gender	
Male/female	285/158
Age at diagnosis	
≤65/>65/NA	197/241/5
Grade	
G1/G2/G3/G4/NA	Unknown
Stage	
I/II/III/IV/NA	59/130/183/44/27
T	
T1/T2/T3/T4/NA	23/93/198/119/10
M	
M0/M1/NA	391/30/22
N	
N0/N1/N2/N3/NA	132/119/85/88/19
*GEO*	
Gender	
Male/female	296/137
Age at diagnosis	
≤65/>65	283/150
Grade	
G1/G2/G3/G4/NA	Unknown
Stage	
I/II/III/IV/NA	Unknown
T	
T1/T2/T3/T4	11/38/92/292
M	
M0/M1/NA	Unknown
N	
N0/N1/N2/N3	80/188/132/33

**Table 2:** The top six enriched functions or pathways.

NAME	ES	NES	NOM *p* value	FDR *q* value
DNA replication	0.6792336	1.622164	0.054435484	0.67065173
Mismatch repair	0.67632705	1.6375834	0.04192872	0.75468564
Spliceosome	0.6235964	1.8821576	0.014522822	0.47306138
Homologous recombination	0.5929925	1.5829824	0.06329114	0.42215267
Cell cycle	0.5691944	1.8317317	0.024896266	0.3576104
Proteasome	0.557963	1.4626185	0.13636364	0.3447309
Other glycan degradation	-0.664515	-1.7258755	0.018480493	0.226103
Alpha linolenic acid metabolism	-0.64434105	-1.8918467	0.004106776	0.10805818
Ribosome	-0.61395043	-1.4429549	0.18145162	0.4314213
Fructose and mannose metabolism	-0.58743125	-1.8711866	0.006085193	0.08654529
Fatty acid metabolism	-0.5829716	-1.9166667	0.006097561	0.16615777
Glycosaminoglycan degradation	-0.5582348	-1.6052122	0.046511628	0.2974447

## Data Availability

The datasets presented in this study can be found in online repositories. The names of the repository/repositories and accession numbers can be found in the article/Supplementary Material. The [Diseases] data used to support the findings of this study have been deposited in the [GEO] repository (https://www.ncbi.nlm.nih.gov/geo/). [TCGA] repository (https://portal.gdc.cancer.gov/).

## Abbreviations

STAD: Stomach adenocarcinoma
GO: Gene ontology
AUC: Areas under the curve
MF: Molecular functions
ICIs: Immune checkpoint inhibitors
ROC: Receiver-operating characteristics
GSEA: Gene set enrichment analyses
KEGG: Kyoto Encyclopedia of Genes and Genomes
TCGA: The Cancer Genome Atlas
PRGs: Pyroptosis-related genes
BP: Biological processes
CC: Cellular components
OS: Overall survival
GEO: Gene Expression Omnibus
DEGs: Differentially expressed genes.

## Supplementary Materials

Supplementary Materials Appendix 1-52 pyroptosis-related genes. Appendix 2-DEGs linked to PRGs. Appendix 3-hub genes analysis. Appendix 4-The gene expression profile and clinical characteristics. Appendix 5-4 risk PRGs. Appendix 6-Table 6. Clinical features for the TCGA cohort. Appendix 7-GO and KEGG enrichment analysis. Appendix 8-gene set enrichment analyses (GSEA).
