# Supplementary material for: Constructing and Validating a Pyroptosis-Related Genes Prognostic Signature for Stomach Adenocarcinoma and Immune Infiltration: Potential Biomarkers for Predicting the Overall Survival
Source: J Oncol. 2022 Sep 26;2022:3102743. doi: 10.1155/2022/3102743 (PMC9529402; doi:10.1155/2022/3102743)
Supplement: Supplementary Materials — Appendix 1-52 pyroptosis-related genes. Appendix 2-DEGs linked to PRGs. Appendix 3-hub genes analysis. Appendix 4-The gene expression profile and clinical characteristics. Appendix 5-4 risk PRGs. Appendix 6-Table 6. Clinical features for the TCGA cohort. Appendix 7-GO and KEGG enrichment analysis. Appendix 8-gene set enrichment analyses (GSEA). [file 3102743.f1.doc]

Constructing and Validating a Pyroptosis-Related Genes Prognostic Signature for Stomach adenocarcinoma and Immune Infiltration: Potential Biomarkers for Predicting the Overall Survival

**Supplementary appendix to the manuscript**

Contents of supplementary appendix

[Appendix 1 3](#__RefHeading___Toc21568)

[52 pyroptosis-related genes 3](#__RefHeading___Toc30542)

[Table 1. 52 pyroptosis-related genes 3](#__RefHeading___Toc22673)

[Appendix 2 4](#__RefHeading___Toc20790)

[DEGs linked to PRGs 4](#__RefHeading___Toc24298)

[Table 2. 29 DEGs linked to PRGs. 4](#__RefHeading___Toc10258)

[Appendix 3 6](#__RefHeading___Toc10820)

[hub genes analysis 6](#__RefHeading___Toc11619)

[Table 3. Hub genes. 6](#__RefHeading___Toc24278)

[Appendix 4 8](#__RefHeading___Toc21051)

[The gene expression profile and clinical characteristics 8](#__RefHeading___Toc30782)

[Table 4. The gene expression profile and clinical characteristics. 8](#__RefHeading___Toc32589)

[Appendix 5 21](#__RefHeading___Toc20937)

[4 risk PRGs 21](#__RefHeading___Toc27278)

[Table 5. 4 risk PRGs. 21](#__RefHeading___Toc23047)

[Appendix 6 35](#__RefHeading___Toc24306)

[Table 6. Clinical features for the TCGA cohort. 35](#__RefHeading___Toc5147)

[Appendix 7 38](#__RefHeading___Toc12045)

[GO and KEGG enrichment analysis 38](#__RefHeading___Toc23651)

[Table 7a. GO enrichment analysis. 38](#__RefHeading___Toc4498)

[Table 7b. KEGG enrichment analysis. 82](#__RefHeading___Toc21345)

[Appendix 8 83](#__RefHeading___Toc24336)

[gene set enrichment analyses (GSEA) 83](#__RefHeading___Toc10524)

[Table 8a. GSEA of high rish. 83](#__RefHeading___Toc8800)

[Table 8b. GSEA of low rish. 92](#__RefHeading___Toc5194)

# Appendix 1

**52 pyroptosis-related genes**

**Table 1. 52 pyroptosis-related genes**

| BAK1 | CHMP4B | IL18 | CASP9 | NLRP6 |
| --- | --- | --- | --- | --- |
| BAX | CHMP4C | IL1A | GPX4 | NLRP7 |
| CASP1 | CHMP6 | IL1B | GSDMA | NOD1 |
| CASP3 | CHMP7 | IRF1 | GSDMB | NOD2 |
| CASP4 | CYCS | IRF2 | GSDMC | PJVK |
| CASP5 | ELANE | TP53 | IL6 | PLCG1 |
| CHMP2A | GSDMD | TP63 | NLRC4 | PRKACA |
| CHMP2B | GSDME | AIM2 | NLRP1 | PYCARD |
| CHMP3 | GZMB | CASP6 | NLRP2 | SCAF11 |
| CHMP4A | HMGB1 | CASP8 | NLRP3 | TIRAP |
| TNF | GZMA |  |  |  |

# Appendix 2

## **DEGs linked to PRGs**

**Table 2. 29 DEGs linked to PRGs.**

| gene | conMean | treatMean | logFC | pValue |
| --- | --- | --- | --- | --- |
| BAX | 12.30830638 | 19.5392392 | 0.666742034 | 3.47E-05 |
| CASP3 | 13.74911113 | 16.61961766 | 0.273548841 | 0.020888993 |
| CASP5 | 1.037437234 | 1.377675699 | 0.409212268 | 0.004987567 |
| CHMP2B | 16.96701906 | 21.19193259 | 0.32078204 | 0.003239613 |
| CHMP4A | 1.102758406 | 1.474568969 | 0.419176544 | 0.000265347 |
| CHMP4C | 10.85897932 | 15.19332267 | 0.484548906 | 0.014065666 |
| CHMP6 | 10.12741319 | 8.475629323 | -0.25687332 | 0.02948712 |
| CHMP7 | 7.698824906 | 9.183392405 | 0.254388933 | 0.030434607 |
| CYCS | 30.75615125 | 46.01526984 | 0.581237709 | 3.45E-05 |
| ELANE | 0.577562665 | 3.586221186 | 2.634415079 | 1.10E-10 |
| GSDMD | 8.936119625 | 10.99795377 | 0.299514724 | 0.043022938 |
| GZMB | 3.964621333 | 9.239726996 | 1.220667147 | 0.013943599 |
| HMGB1 | 23.17294406 | 36.21017696 | 0.643953881 | 1.02E-09 |
| IL18 | 14.91720714 | 11.39745694 | -0.388265495 | 0.004963413 |
| IL1A | 2.709019876 | 1.192280838 | -1.18404688 | 0.000541965 |
| TP53 | 11.98833409 | 22.66621381 | 0.918912227 | 1.23E-06 |
| AIM2 | 1.243142709 | 2.825220199 | 1.184371393 | 0.001700083 |
| CASP6 | 9.423839781 | 12.28357769 | 0.382343902 | 0.002225796 |
| CASP8 | 3.361592688 | 6.841599751 | 1.025188777 | 1.23E-12 |
| CASP9 | 3.360819125 | 2.80616432 | -0.26021341 | 0.025019586 |
| GSDMB | 7.188958144 | 13.96096239 | 0.957543787 | 0.009635167 |
| GSDMC | 2.264864639 | 0.468969043 | -2.271860233 | 1.33E-07 |
| NLRC4 | 0.2847307 | 0.576368318 | 1.01739298 | 5.44E-08 |
| NOD1 | 1.462422306 | 2.198593371 | 0.588220822 | 2.39E-05 |
| PLCG1 | 4.909067638 | 8.071040261 | 0.717305587 | 4.19E-06 |
| PRKACA | 13.48365731 | 12.03603539 | -0.163851613 | 0.046869303 |
| PYCARD | 17.10002866 | 23.16898264 | 0.438195954 | 0.00908054 |
| SCAF11 | 5.815780156 | 9.208553805 | 0.663001866 | 4.07E-12 |
| TNF | 0.869099363 | 1.021774202 | 0.233483383 | 0.028338533 |

# Appendix 3

## **hub genes analysis**

**Table 3. Hub genes.**

| name | Betweenness | Closeness | Degree | Network |
| --- | --- | --- | --- | --- |
| TNF | 76.84126984 | 0.123893805 | 19 | 17.57178932 |
| CASP8 | 28.97460317 | 0.122807018 | 17 | 16.12895715 |
| IL18 | 16.95079365 | 0.12173913 | 16 | 14.79842657 |
| CASP3 | 16.00952381 | 0.121212121 | 14 | 12.12637363 |
| IL1A | 7.66031746 | 0.120171674 | 13 | 11.3290404 |
| CASP9 | 28.37936508 | 0.120689655 | 13 | 9.855880231 |
| PYCARD | 3.365079365 | 0.11965812 | 12 | 10.51616162 |
| HMGB1 | 6.302380952 | 0.11965812 | 12 | 9.660606061 |
| GSDMD | 12.69047619 | 0.11965812 | 12 | 9.941414141 |
| TP53 | 9.111904762 | 0.120171674 | 12 | 9.868506494 |
| NLRC4 | 2.324603175 | 0.119148936 | 11 | 9.763888889 |
| AIM2 | 0.757936508 | 0.118644068 | 10 | 9.430555556 |
| CASP5 | 8.055555556 | 0.118644068 | 10 | 8.486111111 |
| CYCS | 3.25952381 | 0.119148936 | 10 | 8.954365079 |
| NOD1 | 1.154761905 | 0.11814346 | 9 | 8 |
| GZMB | 2.685714286 | 0.11814346 | 9 | 7.832142857 |
| BAX | 40 | 0.11814346 | 8 | 6 |
| CASP6 | 0.571428571 | 0.11814346 | 8 | 7.714285714 |
| ELANE | 0.619047619 | 0.116182573 | 6 | 5.2 |
| CHMP2B | 0 | 0.04 | 4 | 4 |
| CHMP4C | 0 | 0.04 | 4 | 4 |
| CHMP7 | 0 | 0.04 | 4 | 4 |
| CHMP6 | 0 | 0.04 | 4 | 4 |
| CHMP4A | 0 | 0.04 | 4 | 4 |
| SCAF11 | 0.285714286 | 0.114285714 | 3 | 2 |
| PRKACA | 0 | 0.108949416 | 1 | 0 |
| GSDMB | 0 | 0.035714286 | 1 | 0 |
| GSDMC | 0 | 0.035714286 | 1 | 0 |
| PLCG1 | 0 | 0.113821138 | 1 | 0 |

# Appendix 4

# **The gene expression profile and clinical characteristics**

**Table 4. The gene expression profile and clinical characteristics.**

| gene | Mean1 | Mean2 | logFC | pValue | fdr |
| --- | --- | --- | --- | --- | --- |
| KCNJ2 | 1.138328997 | 1.83841181 | 0.691542389 | 7.17E-10 | 1.05E-07 |
| NKX6-3 | 4.451095347 | 2.678033264 | -0.732986525 | 0.000804591 | 0.010485492 |
| PLA2G10 | 7.665214715 | 4.957580401 | -0.628690035 | 5.99E-05 | 0.00149897 |
| SMIM32 | 3.050780805 | 1.649081032 | -0.887516237 | 2.83E-06 | 0.000130165 |
| CD274 | 1.790509795 | 4.157679377 | 1.215408097 | 4.59E-07 | 2.94E-05 |
| EGR3 | 2.611222786 | 4.233167844 | 0.69701214 | 1.15E-07 | 9.24E-06 |
| COL5A2 | 27.44656253 | 44.1897874 | 0.687087516 | 1.06E-07 | 8.59E-06 |
| IBSP | 0.697217198 | 1.267591779 | 0.862410146 | 0.000139362 | 0.00284304 |
| FAP | 1.911144698 | 3.218265076 | 0.751846148 | 4.90E-07 | 3.06E-05 |
| NR4A1 | 15.06543192 | 22.99334866 | 0.609974555 | 2.91E-06 | 0.000132582 |
| ALDH3A1 | 14.75961671 | 7.657713798 | -0.946669611 | 0.001614903 | 0.016925849 |
| CLEC5A | 0.63255546 | 1.463904774 | 1.210557831 | 2.63E-10 | 4.27E-08 |
| ADGRF5 | 4.835234229 | 7.34732525 | 0.603633362 | 1.43E-09 | 1.95E-07 |
| CALB2 | 1.896432799 | 3.097430385 | 0.707783608 | 0.000840928 | 0.010826264 |
| HNF1A-AS1 | 8.094712868 | 5.16275495 | -0.648838785 | 9.89E-07 | 5.56E-05 |
| C3AR1 | 6.892554877 | 10.56626483 | 0.616354721 | 4.37E-05 | 0.001180793 |
| MIR302CHG | 0.808885961 | 0.529827728 | -0.610412974 | 4.41E-06 | 0.000185785 |
| PRDM16-DT | 1.089801931 | 0.661155434 | -0.721004566 | 0.000816424 | 0.010589745 |
| ALPL | 3.15954684 | 6.103580371 | 0.949938125 | 8.60E-05 | 0.001941406 |
| LIF | 6.29883902 | 9.636350077 | 0.613400865 | 1.78E-07 | 1.36E-05 |
| FOXQ1 | 62.0152148 | 41.02800178 | -0.596013318 | 9.49E-07 | 5.39E-05 |
| SELE | 1.261557673 | 3.518609344 | 1.479799185 | 2.16E-16 | 1.24E-13 |
| WNT5A-AS1 | 1.746892975 | 2.686614091 | 0.620997883 | 4.73E-06 | 0.000195431 |
| CHI3L2 | 1.224199186 | 2.066290337 | 0.755204669 | 0.000109503 | 0.002337982 |
| VSIG4 | 7.034190539 | 10.78719275 | 0.616863148 | 0.000165962 | 0.003265812 |
| MIR6730 | 0.811829944 | 0.492492361 | -0.721076206 | 7.18E-05 | 0.001716945 |
| OXER1 | 2.210834977 | 1.398235656 | -0.660983812 | 0.001268611 | 0.014236057 |
| CCL13 | 1.93258816 | 3.414480634 | 0.821131924 | 2.76E-07 | 1.95E-05 |
| CTSK | 34.76968819 | 52.39149164 | 0.591502408 | 0.000263378 | 0.004694586 |
| HAS2-AS1 | 0.509206372 | 0.775080601 | 0.606095872 | 3.98E-06 | 0.00017064 |
| SOD2 | 15.8113659 | 26.9059681 | 0.766964214 | 1.52E-15 | 7.76E-13 |
| MMP10 | 1.79816051 | 5.043677358 | 1.487954183 | 4.27E-12 | 1.00E-09 |
| CCN2 | 82.21366294 | 132.5309873 | 0.688879641 | 3.87E-07 | 2.58E-05 |
| PILRA | 3.498124198 | 5.504302096 | 0.653978141 | 6.30E-06 | 0.000246516 |
| AREG | 14.75677077 | 32.8055671 | 1.152563611 | 1.31E-06 | 6.96E-05 |
| COL7A1 | 3.066187918 | 6.772089061 | 1.143154828 | 1.57E-05 | 0.000521553 |
| GAS1 | 4.794439919 | 7.985623968 | 0.736042848 | 2.33E-05 | 0.000708597 |
| TWIST2 | 1.744036763 | 3.223477569 | 0.886187493 | 1.04E-05 | 0.000373613 |
| TNF | 0.864948543 | 1.35274115 | 0.645199591 | 5.69E-07 | 3.46E-05 |
| SLC39A8 | 3.967129408 | 6.286407565 | 0.66414035 | 4.42E-07 | 2.88E-05 |
| COL15A1 | 24.5540525 | 38.04785315 | 0.631853899 | 2.95E-08 | 2.77E-06 |
| FOSL1 | 13.2802864 | 22.24613687 | 0.744268568 | 0.001570064 | 0.016594392 |
| ADGRE2 | 1.064206017 | 1.718648568 | 0.691497105 | 5.04E-08 | 4.46E-06 |
| NR4A2 | 7.181868796 | 13.09056652 | 0.866096332 | 1.28E-08 | 1.29E-06 |
| CAV1 | 19.05853085 | 31.339586 | 0.71754921 | 4.18E-05 | 0.001138573 |
| FOLH1 | 1.007906382 | 1.702074695 | 0.755932708 | 0.000312443 | 0.005269955 |
| ADAMTS4 | 2.84314966 | 5.820566435 | 1.033669515 | 9.74E-18 | 7.47E-15 |
| SERPINB4 | 1.180902794 | 2.120425854 | 0.844463822 | 3.90E-06 | 0.00016868 |
| SPACA4 | 2.169335584 | 1.374994626 | -0.657827267 | 3.00E-05 | 0.000871229 |
| CD300E | 0.364368702 | 1.698462833 | 2.220758701 | 5.57E-26 | 1.92E-22 |
| CHST1 | 1.838899062 | 2.767229129 | 0.589599814 | 2.82E-06 | 0.000130165 |
| PI15 | 0.701943363 | 4.194239329 | 2.578982651 | 1.40E-18 | 1.21E-15 |
| LILRB3 | 0.95245526 | 1.508691369 | 0.663574475 | 2.96E-07 | 2.07E-05 |
| PLAUR | 16.39036639 | 24.65973837 | 0.589309389 | 1.75E-09 | 2.32E-07 |
| ATF3 | 9.721994139 | 15.92406679 | 0.711884658 | 4.77E-07 | 3.01E-05 |
| STXBP1 | 6.513264202 | 10.42600093 | 0.678733239 | 0.000175051 | 0.003405859 |
| LYVE1 | 1.032672861 | 2.491409936 | 1.270579125 | 2.15E-10 | 3.63E-08 |
| CCL4 | 5.982283395 | 10.55204939 | 0.818755061 | 3.64E-10 | 5.77E-08 |
| PXDN | 7.379463812 | 11.88817515 | 0.687939376 | 1.61E-09 | 2.16E-07 |
| CYP2C9 | 1.709849392 | 1.099700271 | -0.636758891 | 0.000598384 | 0.008434767 |
| APLN | 2.327563217 | 5.697445607 | 1.291494894 | 5.62E-09 | 6.64E-07 |
| HR | 8.837662843 | 5.886241672 | -0.586318117 | 0.000419047 | 0.00652617 |
| CCN4 | 2.605362188 | 4.158854201 | 0.674702164 | 7.49E-07 | 4.44E-05 |
| BDKRB1 | 0.660767006 | 1.072447012 | 0.698692813 | 3.36E-07 | 2.30E-05 |
| PRR16 | 2.008537934 | 3.481461823 | 0.793547495 | 2.52E-11 | 5.12E-09 |
| COL5A3 | 3.984791691 | 6.493828735 | 0.704565028 | 4.89E-08 | 4.36E-06 |
| PAPPA | 1.085743321 | 1.749125497 | 0.687950726 | 3.39E-12 | 8.21E-10 |
| CCL11 | 10.3641418 | 18.36266937 | 0.825175138 | 6.37E-05 | 0.001559034 |
| CCL4L2 | 3.95507179 | 7.275890338 | 0.879419917 | 1.68E-05 | 0.000548696 |
| PBLD | 6.966936133 | 4.278575294 | -0.703393859 | 9.15E-07 | 5.27E-05 |
| MPO | 0.063904454 | 3.227826219 | 5.6585026 | 0.008016448 | 0.049307813 |
| CD300C | 0.907845854 | 1.388968442 | 0.613494557 | 1.50E-06 | 7.66E-05 |
| PTX3 | 0.433592065 | 0.912170057 | 1.07296446 | 2.58E-12 | 6.61E-10 |
| EREG | 1.209764669 | 3.914906655 | 1.694251477 | 4.63E-13 | 1.39E-10 |
| NAMPT | 14.48185923 | 22.70841476 | 0.648980165 | 1.47E-12 | 3.97E-10 |
| MOGAT2 | 4.157881768 | 2.226781135 | -0.900888969 | 0.004486867 | 0.033467378 |
| KIAA1549L | 0.49651723 | 0.897480416 | 0.854036674 | 0.005322727 | 0.037940226 |
| CXCL8 | 16.9687209 | 113.4058711 | 2.740545608 | 8.88E-27 | 6.13E-23 |
| PTGFR | 0.609414852 | 1.080505459 | 0.826209797 | 7.28E-06 | 0.000278627 |
| LINC01235 | 0.707253438 | 1.165783502 | 0.7210007 | 5.42E-05 | 0.001393785 |
| FHL5 | 0.964772229 | 1.454125819 | 0.59189182 | 0.000103228 | 0.002238606 |
| TRPC6 | 0.603382622 | 0.919007069 | 0.607002813 | 2.96E-10 | 4.76E-08 |
| G0S2 | 11.72466102 | 33.79514495 | 1.527269789 | 7.04E-20 | 7.48E-17 |
| SULF1 | 22.42711965 | 35.64112571 | 0.668298559 | 1.19E-05 | 0.000419919 |
| CCL26 | 3.094094987 | 5.317927304 | 0.781346568 | 0.007682241 | 0.048084514 |
| ADH1A | 0.73278963 | 0.446948538 | -0.71329036 | 0.000925338 | 0.011610948 |
| CD163 | 4.723968104 | 8.451158124 | 0.839149834 | 1.30E-06 | 6.91E-05 |
| KCNJ15 | 0.895574857 | 1.416011298 | 0.660946845 | 1.85E-07 | 1.38E-05 |
| INHBA | 7.16612304 | 13.0820922 | 0.868328569 | 5.79E-11 | 1.07E-08 |
| IL1A | 0.44068826 | 2.713690475 | 2.622425806 | 3.95E-24 | 1.09E-20 |
| FST | 2.596856099 | 5.12898372 | 0.981906921 | 1.48E-08 | 1.47E-06 |
| IGFBP2 | 45.85861016 | 30.44249072 | -0.591106231 | 0.000432233 | 0.006686306 |
| LINC00926 | 1.43262748 | 0.84185567 | -0.7670187 | 0.002347272 | 0.021516403 |
| RCAN1 | 5.130921692 | 7.957599718 | 0.633115323 | 1.18E-10 | 2.08E-08 |
| NSRP1P1 | 0.686244274 | 0.453547145 | -0.597469683 | 0.000162586 | 0.003213106 |
| CYP2B7P | 1.481829395 | 0.834976578 | -0.827571724 | 0.004145489 | 0.031534019 |
| GALNT15 | 0.72066641 | 1.280525482 | 0.829332455 | 2.17E-07 | 1.59E-05 |
| CCN1 | 33.23867229 | 70.09659662 | 1.076481644 | 6.23E-15 | 2.61E-12 |
| CCL3L1 | 1.399016672 | 4.559311461 | 1.704402812 | 1.27E-09 | 1.78E-07 |
| TLR4 | 2.657184749 | 4.120610706 | 0.632959632 | 8.24E-05 | 0.001872487 |
| FNDC1 | 9.253753855 | 16.57645179 | 0.841024599 | 0.000447531 | 0.006838705 |
| HSD11B1 | 1.988870276 | 3.769542337 | 0.922440196 | 5.42E-10 | 8.23E-08 |
| C11orf96 | 22.57046716 | 38.49173096 | 0.770112271 | 2.01E-08 | 1.96E-06 |
| IL1B | 4.988351434 | 22.89489503 | 2.198390937 | 1.06E-21 | 1.63E-18 |
| TRPC7-AS1 | 0.835493121 | 0.45871102 | -0.865042382 | 0.000113918 | 0.002413586 |
| CTSL | 27.59702324 | 44.08859506 | 0.675892845 | 4.02E-11 | 7.81E-09 |
| DUSP5 | 8.216481632 | 12.6693243 | 0.624746925 | 5.86E-06 | 0.00023052 |
| RABGAP1L-IT1 | 0.808977214 | 0.457818641 | -0.821322861 | 0.000407815 | 0.006423712 |
| SPIB | 5.080134955 | 1.26024383 | -2.011163932 | 8.94E-05 | 0.002007457 |
| SNHG25 | 26.42448797 | 16.70590483 | -0.661517393 | 0.005289112 | 0.037778591 |
| MTCO1P12 | 256.7517637 | 168.000609 | -0.611907724 | 0.001228307 | 0.013999867 |
| MAP3K4-AS1 | 1.140475935 | 1.781106031 | 0.6431374 | 0.000201086 | 0.003774196 |
| PDZK1 | 1.627790834 | 0.967154728 | -0.751096709 | 0.000556768 | 0.008011657 |
| MIR3945HG | 0.386705227 | 0.862351716 | 1.157042139 | 3.05E-13 | 9.37E-11 |
| MIR320D1 | 2.117382394 | 1.394592342 | -0.602438375 | 0.003078962 | 0.025871518 |
| EPB41L3 | 1.484741871 | 2.502844419 | 0.75335648 | 3.89E-07 | 2.58E-05 |
| ERFE | 0.56205837 | 1.213060069 | 1.109859124 | 6.90E-10 | 1.02E-07 |
| LINC01614 | 1.242255 | 2.217707536 | 0.836107772 | 5.77E-06 | 0.000228485 |
| CMTM2 | 0.463502202 | 0.818684758 | 0.820731843 | 0.000596123 | 0.008411491 |
| IER3 | 73.86332278 | 115.4220164 | 0.64398837 | 2.00E-08 | 1.96E-06 |
| RNY1P16 | 3.268052901 | 2.095112052 | -0.641403932 | 0.00183788 | 0.018332398 |
| FN1 | 81.29011932 | 129.3087521 | 0.669668015 | 6.64E-07 | 3.95E-05 |
| GFPT2 | 1.859736725 | 3.773272992 | 1.020718082 | 6.88E-09 | 7.67E-07 |
| KLHDC7A | 1.439583807 | 0.880235841 | -0.709689758 | 0.001850849 | 0.01842048 |
| IL11 | 1.063270097 | 6.475139263 | 2.606403098 | 6.77E-20 | 7.48E-17 |
| RNVU1-7 | 1.076618825 | 0.703687997 | -0.613499746 | 0.005780131 | 0.039883482 |
| NPTX2 | 1.343021334 | 2.882727268 | 1.101952129 | 0.000113249 | 0.002403096 |
| HSPA6 | 2.271356161 | 6.58779885 | 1.536242559 | 1.90E-12 | 5.04E-10 |
| CLEC4E | 0.975996562 | 2.417510356 | 1.308574098 | 6.84E-09 | 7.67E-07 |
| EN2 | 0.7240314 | 0.435990306 | -0.731756208 | 0.001985926 | 0.019415134 |
| NLRP3 | 0.547555137 | 1.123376923 | 1.03676592 | 1.68E-10 | 2.94E-08 |
| ESM1 | 2.52120563 | 4.532679826 | 0.846250468 | 5.48E-07 | 3.35E-05 |
| MCEMP1 | 0.424952928 | 1.154119374 | 1.441417506 | 1.25E-12 | 3.47E-10 |
| SNRPEP11 | 0.435592771 | 0.729070376 | 0.743078071 | 0.002233077 | 0.020956333 |
| FFAR2 | 0.918139358 | 1.557533819 | 0.762478438 | 5.42E-07 | 3.33E-05 |
| OLR1 | 2.780579066 | 4.232401763 | 0.606091222 | 2.88E-05 | 0.000843657 |
| BTNL8 | 4.506696525 | 2.75063418 | -0.712306023 | 0.003328677 | 0.027289225 |
| CASS4 | 0.515922977 | 0.780672816 | 0.597562334 | 5.14E-06 | 0.00020803 |
| LINC01978 | 1.541371713 | 0.953967267 | -0.692203151 | 2.95E-05 | 0.000857459 |
| TNFAIP6 | 2.907984814 | 10.16339282 | 1.805290453 | 4.85E-19 | 4.46E-16 |
| RNU6-877P | 1.035032349 | 0.646448578 | -0.679068338 | 0.000122316 | 0.002548523 |
| LILRB2 | 2.004239441 | 3.12270263 | 0.639740316 | 1.70E-07 | 1.32E-05 |
| ALOX5AP | 12.53806048 | 18.94171741 | 0.595252949 | 1.93E-05 | 0.000614258 |
| PLEK | 7.720009755 | 11.7576895 | 0.606930009 | 3.28E-06 | 0.000146078 |
| PDE10A | 0.641755263 | 1.107609746 | 0.787354527 | 0.000106771 | 0.002283178 |
| ADAMTS1 | 5.962760789 | 11.1561146 | 0.903782293 | 1.27E-08 | 1.29E-06 |
| ADH1C | 45.4467416 | 20.22273469 | -1.168198757 | 0.000184607 | 0.003571661 |
| MRC1 | 3.31737452 | 6.298360081 | 0.924934343 | 4.52E-07 | 2.92E-05 |
| MMP1 | 45.37840235 | 141.4650933 | 1.640368388 | 1.02E-13 | 3.29E-11 |
| POSTN | 42.20145785 | 66.34811865 | 0.65276272 | 8.50E-07 | 4.95E-05 |
| PTHLH | 0.815355442 | 1.421397549 | 0.801809093 | 7.86E-05 | 0.001804162 |
| LOX | 6.696954147 | 11.82070744 | 0.819739385 | 2.32E-09 | 3.00E-07 |
| RNU6-1093P | 0.663752481 | 0.406436887 | -0.707614005 | 0.001123209 | 0.013165867 |
| MTND1P23 | 307.4291192 | 59.6013767 | -2.366836261 | 2.12E-05 | 0.000665702 |
| RNU1-153P | 2.44550001 | 1.539106383 | -0.668036516 | 0.000105393 | 0.002272071 |
| GSTA1 | 32.34615233 | 12.317352 | -1.392901973 | 0.000489889 | 0.007316027 |
| LINC00839 | 0.429492107 | 0.658787656 | 0.617181907 | 9.98E-06 | 0.000360976 |
| SLC38A5 | 5.235086866 | 8.193139256 | 0.64620286 | 0.002679124 | 0.023542885 |
| NNMT | 16.30597876 | 24.47364777 | 0.585828109 | 2.00E-06 | 9.78E-05 |
| CCDC190 | 0.948278466 | 0.403718024 | -1.231962776 | 0.003343218 | 0.027392179 |
| MEDAG | 2.903899879 | 7.270909311 | 1.324144088 | 5.08E-11 | 9.61E-09 |
| CSF3R | 1.831898533 | 3.339696656 | 0.866377473 | 1.36E-06 | 7.18E-05 |
| ADGRG3 | 0.569038152 | 1.360799708 | 1.257857449 | 9.29E-10 | 1.32E-07 |
| NR4A3 | 1.866519685 | 4.631401946 | 1.311098246 | 9.91E-16 | 5.27E-13 |
| RGS16 | 5.619378355 | 10.10211563 | 0.846175014 | 6.87E-15 | 2.79E-12 |
| S100A12 | 1.715402794 | 8.688744449 | 2.340600343 | 5.02E-16 | 2.77E-13 |
| THBS1-IT1 | 0.538730795 | 1.141098708 | 1.082787154 | 2.81E-09 | 3.52E-07 |
| MMP13 | 0.406186575 | 1.618442616 | 1.994391752 | 3.06E-09 | 3.81E-07 |
| LGALS2 | 41.12130528 | 23.1423472 | -0.829350863 | 0.002307565 | 0.021322209 |
| MT-TA | 1.245543951 | 0.752638467 | -0.726746998 | 0.000190235 | 0.003644173 |
| FPR1 | 2.164990919 | 5.939416317 | 1.455960187 | 5.12E-12 | 1.18E-09 |
| NAMPTP1 | 1.313337404 | 2.643295284 | 1.009099998 | 2.07E-08 | 2.00E-06 |
| NCF2 | 4.088116288 | 6.320655676 | 0.62863799 | 3.10E-06 | 0.000140903 |
| LILRA6 | 0.416274759 | 0.75824208 | 0.86512244 | 6.02E-08 | 5.23E-06 |
| AOX1 | 1.062082697 | 1.922800099 | 0.85631268 | 0.00020941 | 0.003898647 |
| TFPI2 | 1.35273741 | 4.098331836 | 1.599154987 | 2.56E-08 | 2.44E-06 |
| CXCR1 | 0.401907945 | 1.439306254 | 1.840436599 | 3.83E-09 | 4.68E-07 |
| FCGR2A | 7.576164328 | 13.04059251 | 0.783469892 | 1.13E-11 | 2.40E-09 |
| LOXL2 | 11.2225794 | 16.89206926 | 0.589941765 | 4.02E-08 | 3.65E-06 |
| SIRPB2 | 0.478585915 | 0.771091653 | 0.688124413 | 4.47E-07 | 2.90E-05 |
| CCL7 | 0.529196238 | 1.290286837 | 1.285817109 | 1.05E-12 | 3.01E-10 |
| MIR3131 | 6.247386765 | 3.97057866 | -0.653903571 | 0.001095824 | 0.012971476 |
| NID2 | 4.872802734 | 7.888553089 | 0.695008888 | 2.41E-09 | 3.09E-07 |
| MT2P1 | 1.60927478 | 2.472362332 | 0.619479506 | 3.11E-06 | 0.000140903 |
| RPL29P19 | 0.423058801 | 0.738381231 | 0.803507682 | 0.000106321 | 0.002280637 |
| PCDH17 | 2.426427987 | 3.71948127 | 0.616267389 | 3.86E-08 | 3.53E-06 |
| MMP2 | 63.21194009 | 99.65737053 | 0.656779414 | 2.32E-05 | 0.000708491 |
| ADAMTS9 | 2.190729121 | 3.358772471 | 0.616522959 | 6.27E-08 | 5.41E-06 |
| F13A1 | 4.291543375 | 7.264222361 | 0.759311785 | 0.00138213 | 0.015189132 |
| GPX3 | 17.39749211 | 28.65557754 | 0.71993662 | 0.000628507 | 0.008725828 |
| FOSB | 10.65338127 | 20.29775789 | 0.930008975 | 3.03E-05 | 0.000873609 |
| ALDH1A1 | 111.1715031 | 67.96103904 | -0.71000721 | 0.006857276 | 0.044619132 |
| HAS2 | 1.188275399 | 2.535708015 | 1.093519391 | 2.22E-12 | 5.78E-10 |
| SRGN | 77.03439424 | 139.626374 | 0.85799685 | 1.32E-07 | 1.06E-05 |
| BCL2A1 | 5.742193826 | 12.02967212 | 1.066923387 | 2.44E-10 | 4.02E-08 |
| CLEC4A | 1.810409577 | 3.09025009 | 0.771407477 | 4.10E-07 | 2.71E-05 |
| GRAMD1B | 3.271315289 | 2.132440594 | -0.617365262 | 0.00209635 | 0.020054691 |
| CDH13 | 1.471772793 | 2.35262676 | 0.676717487 | 4.31E-10 | 6.77E-08 |
| PLIN2 | 8.129306468 | 12.83879107 | 0.659305179 | 1.48E-09 | 2.01E-07 |
| COL4A1 | 63.57026071 | 102.0043984 | 0.682207452 | 1.41E-09 | 1.95E-07 |
| PLAU | 20.99403359 | 39.21822576 | 0.901544891 | 1.09E-13 | 3.42E-11 |
| STC1 | 8.636123951 | 15.44783298 | 0.838948617 | 9.77E-14 | 3.21E-11 |
| TWIST1 | 1.830824777 | 2.759686756 | 0.592010799 | 5.11E-06 | 0.00020803 |
| TRIM31 | 22.44909832 | 14.72673175 | -0.608220206 | 4.30E-06 | 0.000181824 |
| FPR2 | 0.350262004 | 1.46090067 | 2.060351691 | 2.04E-15 | 9.73E-13 |
| RNU6-353P | 1.22678707 | 0.65651969 | -0.901974682 | 0.000119703 | 0.002509227 |
| CD36 | 1.393206914 | 2.580917763 | 0.889474635 | 0.001690286 | 0.017321666 |
| COL11A2 | 1.691661755 | 0.916718052 | -0.883891145 | 0.000483352 | 0.007249755 |
| STEAP4 | 1.669589178 | 2.542245253 | 0.606610061 | 0.000810399 | 0.010521481 |
| PGAP3 | 32.53438823 | 21.22458137 | -0.616229327 | 7.06E-05 | 0.001702329 |
| CXCL3 | 15.14366128 | 23.93899778 | 0.660648706 | 0.000278499 | 0.004869846 |
| SLC11A1 | 1.128873083 | 2.552806275 | 1.177200764 | 3.23E-14 | 1.17E-11 |
| PDGFRL | 1.689895092 | 2.760840757 | 0.708173989 | 0.002276243 | 0.021202981 |
| MARCO | 1.52901497 | 2.504628389 | 0.711994035 | 5.31E-06 | 0.000214633 |
| ENHO | 0.627013312 | 0.383877945 | -0.707848398 | 0.000184549 | 0.003571661 |
| SMPD3 | 8.624518096 | 5.301042788 | -0.702167661 | 2.59E-07 | 1.85E-05 |
| HILPDA | 5.146020074 | 9.409691285 | 0.870690307 | 8.01E-08 | 6.67E-06 |
| RASAL1 | 5.21168492 | 3.265338884 | -0.674517141 | 0.001032621 | 0.012512832 |
| C5AR1 | 6.803167847 | 12.44700856 | 0.871520466 | 1.17E-12 | 3.31E-10 |
| EGFLAM | 0.784017514 | 1.267269453 | 0.692765523 | 9.41E-10 | 1.33E-07 |
| HAS1 | 0.310657896 | 1.035576561 | 1.737035588 | 5.99E-15 | 2.58E-12 |
| CHSY3 | 0.825063018 | 1.264714605 | 0.616235642 | 1.27E-08 | 1.29E-06 |
| CLMP | 4.272930658 | 6.859810728 | 0.682942866 | 0.000589389 | 0.008359162 |
| SCGN | 2.897397847 | 1.88350103 | -0.621340975 | 0.004434621 | 0.033182454 |
| ADAM12 | 1.875555164 | 3.680410615 | 0.972549037 | 6.18E-10 | 9.28E-08 |
| RAB39A | 0.443106656 | 0.720222589 | 0.700788852 | 0.007181617 | 0.045886614 |
| MMP3 | 6.54401895 | 45.98281876 | 2.812846075 | 2.82E-17 | 1.95E-14 |
| SPOCK1 | 5.170481759 | 8.35183113 | 0.691793832 | 0.000172913 | 0.003378534 |
| PDE4B | 2.30605495 | 3.932899255 | 0.77016634 | 4.36E-11 | 8.37E-09 |
| BCAT1 | 2.227125399 | 4.57276641 | 1.037884432 | 5.16E-08 | 4.54E-06 |
| MS4A4A | 4.343248651 | 6.730197357 | 0.631874262 | 0.000548357 | 0.007915368 |
| AQP9 | 1.25894262 | 4.816596396 | 1.93580151 | 3.21E-14 | 1.17E-11 |
| GK-IT1 | 0.755174576 | 1.407635188 | 0.898391384 | 3.51E-06 | 0.000155102 |
| FGF7 | 2.560787183 | 4.950455974 | 0.950974053 | 1.65E-06 | 8.32E-05 |
| JCAD | 3.7532848 | 6.103977511 | 0.701595881 | 5.39E-06 | 0.000216531 |
| FTH1P23 | 2.446649028 | 3.740730129 | 0.612512726 | 3.22E-08 | 2.98E-06 |
| HCAR2 | 0.55758959 | 1.937263811 | 1.796744898 | 2.31E-17 | 1.68E-14 |
| RNU6-731P | 0.859649601 | 0.561869487 | -0.613513671 | 0.000373309 | 0.006024408 |
| TENM3 | 0.736017962 | 1.113608621 | 0.597429405 | 4.03E-05 | 0.001107615 |
| THBS1-AS1 | 0.480468693 | 1.121133091 | 1.222443217 | 8.33E-08 | 6.85E-06 |
| P4HA3 | 0.826032037 | 1.277539128 | 0.629097837 | 3.55E-06 | 0.000156155 |
| MST1L | 1.308178088 | 0.84104738 | -0.637299974 | 0.001063091 | 0.012714758 |
| GEM | 14.79484993 | 23.10143834 | 0.642887616 | 9.89E-06 | 0.000360395 |
| MNDA | 4.093836393 | 7.312475427 | 0.836906424 | 4.84E-06 | 0.000199087 |
| CD80 | 0.50409143 | 0.763961393 | 0.599814305 | 1.76E-06 | 8.76E-05 |
| IL6 | 0.943468909 | 10.1388187 | 3.425770783 | 4.26E-53 | 5.88E-49 |
| MIR223HG | 0.561177024 | 1.201917493 | 1.098810015 | 4.80E-06 | 0.000197735 |
| RNU6-1340P | 0.914146815 | 0.60398649 | -0.597909607 | 0.001587947 | 0.016732189 |
| GPR4 | 3.192118979 | 4.824422581 | 0.595841855 | 1.80E-10 | 3.10E-08 |
| MAP1LC3B2 | 0.652560918 | 1.000725124 | 0.616861261 | 0.004105339 | 0.031314828 |
| FCGR3B | 1.262771617 | 3.06409305 | 1.278866371 | 3.38E-07 | 2.30E-05 |
| GPR176 | 1.724887031 | 2.741084161 | 0.668244747 | 4.77E-07 | 3.01E-05 |
| CLEC12A | 0.461367404 | 0.705642705 | 0.613021793 | 0.002495455 | 0.022472108 |
| APOBEC3A | 0.545825989 | 1.353098817 | 1.309754209 | 5.70E-15 | 2.54E-12 |
| GDPD2 | 1.13931772 | 0.701850201 | -0.698935077 | 0.004281907 | 0.032340217 |
| RNA5SP207 | 0.819760067 | 1.44323564 | 0.816033253 | 0.000126089 | 0.00261913 |
| COL12A1 | 20.5877743 | 34.98083472 | 0.764776844 | 1.04E-07 | 8.45E-06 |
| CFAP73 | 0.651950667 | 0.304282939 | -1.099349357 | 6.36E-07 | 3.82E-05 |
| WNT5A | 4.390415418 | 6.935770569 | 0.659698723 | 4.02E-05 | 0.001107615 |
| HBA2 | 12.16673859 | 23.09924213 | 0.924903026 | 2.41E-06 | 0.000115037 |
| ACKR3 | 7.609945956 | 12.40488653 | 0.704950426 | 1.71E-11 | 3.58E-09 |
| NFIL3 | 10.11020824 | 15.5093327 | 0.617323902 | 1.94E-16 | 1.16E-13 |
| DUSP1 | 82.72264823 | 148.7244808 | 0.846287865 | 1.04E-10 | 1.87E-08 |
| MROH3P | 0.57977261 | 0.370701778 | -0.645228146 | 0.005123842 | 0.036923045 |
| CYP4F12 | 3.179162161 | 1.801797819 | -0.819209472 | 3.25E-06 | 0.00014557 |
| TMEM158 | 7.986901045 | 12.11062155 | 0.600565165 | 0.000199459 | 0.00375898 |
| PDPN | 7.574315306 | 13.28174127 | 0.810256916 | 1.51E-08 | 1.49E-06 |
| LAMC3 | 0.686581011 | 1.493628216 | 1.121319225 | 0.000959946 | 0.011893003 |
| RGS2 | 17.996518 | 27.00573358 | 0.585547939 | 9.84E-07 | 5.55E-05 |
| CYTL1 | 0.857311478 | 1.856825073 | 1.114946546 | 5.80E-09 | 6.73E-07 |
| ADM | 4.390627444 | 9.76172229 | 1.152708586 | 2.09E-14 | 8.01E-12 |
| SPP1 | 66.80741397 | 131.931126 | 0.981704854 | 5.14E-06 | 0.00020803 |
| FAM83E | 18.58158806 | 12.37899009 | -0.585980185 | 1.03E-06 | 5.77E-05 |
| FOS | 81.07319823 | 124.4933182 | 0.61877135 | 2.91E-06 | 0.000132582 |
| RETN | 0.430264365 | 0.957020078 | 1.153325834 | 4.99E-05 | 0.001319619 |
| CXCL1 | 42.90197698 | 103.0213908 | 1.263827886 | 6.18E-12 | 1.38E-09 |
| SNAI2 | 4.659666559 | 7.12073771 | 0.611799992 | 4.85E-09 | 5.77E-07 |
| RNU4-38P | 0.635977842 | 0.392954745 | -0.694613329 | 0.000383762 | 0.006135758 |
| TLR2 | 2.589045953 | 4.251086366 | 0.715410998 | 1.38E-07 | 1.09E-05 |
| ENTPD2 | 13.6433296 | 8.839444529 | -0.626168153 | 0.001793748 | 0.017994795 |
| COL9A1 | 1.012034703 | 0.654879637 | -0.627957084 | 0.001696216 | 0.017343843 |
| CHI3L1 | 10.22154588 | 21.02852254 | 1.040734088 | 5.42E-07 | 3.33E-05 |
| IL17REL | 1.107616248 | 0.634136729 | -0.804592278 | 0.006752121 | 0.044184674 |
| CCL8 | 1.641261489 | 4.23339731 | 1.367010783 | 8.14E-13 | 2.39E-10 |
| CSF3 | 1.607302735 | 8.391942068 | 2.384363033 | 4.94E-26 | 1.92E-22 |
| HBB | 15.88982648 | 41.20086953 | 1.374571415 | 8.62E-09 | 9.16E-07 |
| TNC | 21.56646346 | 32.50039055 | 0.591667436 | 4.09E-08 | 3.69E-06 |
| CCL3 | 2.139434818 | 6.092647894 | 1.509839641 | 9.98E-17 | 6.56E-14 |
| COL3A1 | 289.4798877 | 453.9559136 | 0.649089079 | 9.21E-05 | 0.002044841 |
| CCL18 | 20.48025747 | 46.60950732 | 1.186390411 | 1.55E-05 | 0.000518055 |
| ANGPTL4 | 3.492284018 | 7.503170944 | 1.103329533 | 7.73E-14 | 2.67E-11 |
| CLC | 1.119706653 | 2.980459893 | 1.412414143 | 4.03E-05 | 0.001107615 |
| SOCS3 | 30.4179495 | 53.25402323 | 0.807967619 | 1.59E-16 | 9.98E-14 |
| CEMIP | 7.991807607 | 17.40196057 | 1.122656097 | 1.42E-08 | 1.42E-06 |
| CCL2 | 14.59925877 | 25.81294752 | 0.822199766 | 3.41E-14 | 1.21E-11 |
| ADAMTS5 | 1.241637359 | 1.926245611 | 0.633547798 | 3.30E-09 | 4.07E-07 |
| THBS1 | 36.59393514 | 80.0734203 | 1.129718867 | 3.06E-12 | 7.54E-10 |
| LILRA5 | 0.943621163 | 1.74955967 | 0.89071219 | 2.80E-11 | 5.60E-09 |
| CXCR2 | 0.752462948 | 1.7856036 | 1.246719393 | 8.15E-08 | 6.74E-06 |
| AKAP12 | 6.674662257 | 10.0985817 | 0.597385945 | 0.002982849 | 0.025279185 |
| S100A3 | 1.688052077 | 3.118933364 | 0.885693317 | 8.16E-09 | 8.81E-07 |
| SLC2A3 | 5.380512779 | 11.7400264 | 1.125620075 | 2.49E-21 | 3.44E-18 |
| TUSC3 | 2.369701127 | 3.822578324 | 0.689840949 | 0.00243684 | 0.022117285 |
| AVPR1A | 0.781591825 | 1.29323893 | 0.72650156 | 2.25E-08 | 2.16E-06 |
| HBA1 | 2.166742332 | 3.360393271 | 0.633102484 | 0.005887459 | 0.040402088 |
| PRRX1 | 3.579517332 | 5.941824875 | 0.73114102 | 1.28E-06 | 6.83E-05 |
| SERPINE1 | 26.99549134 | 56.92158644 | 1.076257396 | 4.14E-19 | 4.09E-16 |
| MMP19 | 2.432065142 | 4.905231594 | 1.012139382 | 3.81E-12 | 9.06E-10 |
| STC2 | 1.499692938 | 2.250347585 | 0.585480716 | 4.26E-07 | 2.80E-05 |
| EXOC3L4 | 2.171303451 | 1.441900204 | -0.590590047 | 3.50E-06 | 0.000154833 |
| MTND4P20 | 1.542775299 | 0.965390043 | -0.676344101 | 0.003414926 | 0.027798344 |
| TREM1 | 0.685718579 | 2.201620317 | 1.682877172 | 7.93E-18 | 6.44E-15 |
| PALM3 | 4.066170973 | 2.494816776 | -0.704737013 | 0.001043952 | 0.012594888 |
| CXCL2 | 16.05721559 | 31.47085144 | 0.970794468 | 5.15E-11 | 9.61E-09 |
| ACAN | 0.979928257 | 1.907618858 | 0.961024916 | 4.59E-09 | 5.56E-07 |
| RNU6-1111P | 0.738895464 | 0.467168436 | -0.661427471 | 0.004973192 | 0.0361197 |
| ZFP36 | 144.4133136 | 225.9857656 | 0.646028151 | 5.62E-08 | 4.91E-06 |
| TLR8 | 0.921295631 | 1.505026615 | 0.708052923 | 0.000576133 | 0.008221796 |
| MMP12 | 21.74594269 | 56.59074605 | 1.379819905 | 2.79E-07 | 1.96E-05 |
| SNAI1 | 4.314718652 | 6.849994242 | 0.666836286 | 4.92E-15 | 2.27E-12 |
| SFRP5 | 2.913728109 | 1.364797362 | -1.094179497 | 0.002808256 | 0.024306545 |
| IZUMO4 | 0.626867058 | 0.353139265 | -0.827922279 | 0.000744019 | 0.009920736 |
| HIF1A-AS3 | 0.481741975 | 0.74772206 | 0.634241466 | 8.98E-05 | 0.002009484 |
| OSM | 1.00500922 | 4.235681545 | 2.075385389 | 2.21E-22 | 5.09E-19 |
| MMP9 | 16.91491443 | 29.75874131 | 0.815017628 | 1.22E-06 | 6.66E-05 |
| CYP26B1 | 0.897367306 | 1.708614263 | 0.929056203 | 2.03E-06 | 9.86E-05 |
| KCNE4 | 2.095314305 | 3.144990043 | 0.58588878 | 0.000344777 | 0.005643071 |
| CSF2 | 0.352512244 | 2.750184995 | 2.963783395 | 2.01E-20 | 2.52E-17 |
| RASD1 | 3.92853912 | 12.6424314 | 1.686209119 | 6.91E-08 | 5.89E-06 |
| CD93 | 11.17486617 | 18.68343848 | 0.741502437 | 2.88E-11 | 5.68E-09 |
| PROK2 | 0.205760887 | 1.414848491 | 2.781606895 | 6.15E-22 | 1.06E-18 |
| IL13RA2 | 0.554836843 | 1.670036563 | 1.589744193 | 2.39E-10 | 3.99E-08 |
| GK-AS1 | 0.514933444 | 1.136324096 | 1.141916492 | 2.14E-05 | 0.000670336 |
| FCN1 | 0.610446615 | 1.425894325 | 1.223930025 | 8.49E-11 | 1.54E-08 |
| SERPINB2 | 3.089010213 | 5.403108011 | 0.806644882 | 3.18E-07 | 2.20E-05 |
| CXCL6 | 2.39348491 | 7.599960823 | 1.666879271 | 8.60E-12 | 1.88E-09 |
| CH25H | 2.365956126 | 3.599794346 | 0.605491168 | 0.004052355 | 0.031013427 |
| EGFL6 | 1.432260518 | 2.964671985 | 1.049578559 | 5.69E-09 | 6.66E-07 |
| PLA2G1B | 26.34491121 | 0.966649275 | -4.768387969 | 0.00030873 | 0.005239818 |
| PTGS2 | 4.230708443 | 10.07488275 | 1.251791878 | 3.18E-22 | 6.27E-19 |
| GPR84 | 0.588324888 | 1.427793024 | 1.279101884 | 1.42E-14 | 5.61E-12 |
| IL24 | 1.56326135 | 6.388819892 | 2.030990477 | 1.90E-15 | 9.36E-13 |
| WNT2 | 2.088070223 | 3.23751958 | 0.632718687 | 0.000193862 | 0.003688711 |
| AKR7A3 | 33.04395828 | 21.37430753 | -0.628508834 | 0.000856608 | 0.0109668 |

# Appendix 5

**4 risk PRGs**

**Table 5. 4 risk PRGs.**

| id | GPX3 | PDGFRL | RGS2 | SERPINE1 | riskScore | risk |
| --- | --- | --- | --- | --- | --- | --- |
| TCGA-D7-A74A | 4.907775 | 3.763067 | 7.217543 | 4.930129 | 0.692161469 | low |
| TCGA-BR-7704 | 6.236945 | 4.2858 | 7.080429 | 7.099063 | 0.939513468 | high |
| TCGA-VQ-A91N | 5.533767 | 5.755545 | 8.093103 | 4.797128 | 0.749875074 | low |
| TCGA-CD-A4MH | 5.311407 | 4.331666 | 7.70723 | 4.802088 | 0.707330106 | low |
| TCGA-EQ-8122 | 6.449737 | 4.610825 | 8.099511 | 8.006471 | 1.037209107 | high |
| TCGA-VQ-A91Y | 6.655876 | 4.928568 | 9.260021 | 7.603821 | 1.016423418 | high |
| TCGA-CG-4438 | 7.032119 | 3.827407 | 9.163562 | 5.735198 | 0.830575835 | low |
| TCGA-BR-4366 | 7.37066 | 6.138452 | 9.981313 | 7.181746 | 1.030587505 | high |
| TCGA-VQ-AA64 | 7.413765 | 6.851519 | 6.902842 | 7.510689 | 1.075429932 | high |
| TCGA-HF-7132 | 7.11923 | 4.067091 | 7.457818 | 5.620691 | 0.826520451 | low |
| TCGA-CG-5717 | 9.199177 | 4.238474 | 8.040343 | 6.157344 | 0.940281707 | high |
| TCGA-BR-A4J8 | 6.757749 | 5.122593 | 8.657533 | 5.740006 | 0.855169929 | low |
| TCGA-CG-5732 | 7.412664 | 3.996349 | 8.288578 | 4.743803 | 0.755160084 | low |
| TCGA-CG-4477 | 8.15727 | 4.4605 | 9.678703 | 7.384183 | 1.028748361 | high |
| TCGA-HU-A4G3 | 6.45934 | 4.79911 | 7.666595 | 4.984335 | 0.768775696 | low |
| TCGA-IN-AB1X | 7.295052 | 3.648735 | 6.900973 | 6.772725 | 0.924293408 | high |
| TCGA-CD-5804 | 6.43625 | 5.690071 | 7.882777 | 6.961561 | 0.969489015 | high |
| TCGA-CG-5721 | 6.740811 | 4.505395 | 7.082061 | 5.991677 | 0.859657294 | low |
| TCGA-CD-A489 | 7.352629 | 4.850375 | 9.909424 | 6.532789 | 0.938768016 | high |
| TCGA-RD-A8N4 | 7.612557 | 5.385352 | 10.349972 | 5.800142 | 0.894266968 | low |
| TCGA-BR-6566 | 6.904532 | 5.288945 | 7.890646 | 5.663681 | 0.855700014 | low |
| TCGA-HU-8244 | 5.014345 | 3.787867 | 6.143625 | 3.491728 | 0.564538409 | low |
| TCGA-FP-A9TM | 5.08327 | 3.929469 | 7.606946 | 4.813732 | 0.691465895 | low |
| TCGA-MX-A5UG | 7.170851 | 4.971055 | 9.630288 | 7.832566 | 1.053553394 | high |
| TCGA-VQ-A94U | 6.728566 | 4.67459 | 6.794421 | 6.207101 | 0.882649418 | low |
| TCGA-D7-A4Z0 | 6.551234 | 3.856469 | 9.305884 | 7.717298 | 0.996569554 | high |
| TCGA-BR-8590 | 7.818239 | 6.642841 | 8.811849 | 6.49083 | 0.992336543 | high |
| TCGA-HU-8610 | 6.253272 | 3.675367 | 6.966092 | 7.633475 | 0.972637667 | high |
| TCGA-VQ-AA69 | 5.053535 | 3.651528 | 5.799505 | 6.922698 | 0.871566275 | low |
| TCGA-CG-5723 | 7.265606 | 5.840467 | 8.51556 | 6.482806 | 0.954914702 | high |
| TCGA-RD-A8N2 | 6.954825 | 5.215175 | 9.6676 | 4.667227 | 0.767708896 | low |
| TCGA-BR-7722 | 6.800534 | 4.032839 | 7.763278 | 5.790232 | 0.832154275 | low |
| TCGA-D7-8576 | 6.481392 | 4.10914 | 8.500693 | 5.459717 | 0.796002373 | low |
| TCGA-BR-8058 | 6.573068 | 4.460613 | 8.317332 | 6.444736 | 0.896248228 | high |
| TCGA-VQ-A91E | 6.01636 | 5.212374 | 7.415323 | 5.675732 | 0.828511304 | low |
| TCGA-VQ-A8PO | 5.793252 | 4.531302 | 8.80601 | 7.003226 | 0.926565211 | high |
| TCGA-HU-8249 | 5.577633 | 3.791358 | 7.242754 | 6.184911 | 0.825596519 | low |
| TCGA-CG-5725 | 7.190393 | 3.779675 | 7.17104 | 6.187847 | 0.872130745 | low |
| TCGA-HU-A4H4 | 6.733263 | 3.849724 | 7.08797 | 4.90073 | 0.744341619 | low |
| TCGA-VQ-A8PJ | 6.332999 | 4.260372 | 7.884626 | 5.663821 | 0.813135228 | low |
| TCGA-BR-6565 | 7.135417 | 5.355714 | 7.757023 | 6.007571 | 0.894942249 | high |
| TCGA-VQ-A923 | 6.214766 | 3.733588 | 7.337097 | 6.502216 | 0.871347683 | low |
| TCGA-D7-A4YU | 6.421493 | 4.140852 | 7.336365 | 4.037277 | 0.665057055 | low |
| TCGA-VQ-AA6K | 5.905224 | 4.13186 | 7.215595 | 8.365053 | 1.04051826 | high |
| TCGA-BR-8679 | 5.95735 | 5.286264 | 6.977761 | 5.398384 | 0.803039588 | low |
| TCGA-F1-A448 | 6.439242 | 4.195655 | 9.194076 | 8.402072 | 1.063596848 | high |
| TCGA-CD-8535 | 5.435711 | 4.826518 | 6.288534 | 6.031567 | 0.832555659 | low |
| TCGA-BR-6852 | 7.045434 | 5.614687 | 6.438674 | 7.162171 | 1.001371805 | high |
| TCGA-BR-6458 | 7.706971 | 7.171386 | 8.129041 | 7.657846 | 1.106952153 | high |
| TCGA-ZA-A8F6 | 7.451555 | 5.202757 | 8.77751 | 4.944236 | 0.805578234 | low |
| TCGA-BR-7196 | 7.739388 | 6.188653 | 9.197256 | 7.974723 | 1.113072213 | high |
| TCGA-IN-A6RI | 5.453501 | 4.784209 | 8.207747 | 4.685001 | 0.712998079 | low |
| TCGA-HJ-7597 | 5.430731 | 4.254041 | 6.427545 | 7.127334 | 0.917055183 | high |
| TCGA-HU-A4GQ | 6.296534 | 5.319687 | 8.453381 | 5.99977 | 0.869994734 | low |
| TCGA-B7-5818 | 7.006372 | 4.171456 | 6.932627 | 6.20169 | 0.8776507 | low |
| TCGA-BR-8588 | 7.385385 | 5.243423 | 9.304594 | 7.376096 | 1.024996225 | high |
| TCGA-VQ-A8DU | 6.451493 | 4.590191 | 7.133716 | 5.240399 | 0.785663648 | low |
| TCGA-HU-A4G9 | 4.282911 | 3.429169 | 5.977909 | 3.321376 | 0.518691216 | low |
| TCGA-CD-A4MG | 6.053289 | 5.903613 | 7.388416 | 6.523673 | 0.923606017 | high |
| TCGA-HU-8602 | 5.938724 | 3.784766 | 7.613151 | 8.186532 | 1.017119091 | high |
| TCGA-VQ-A91S | 6.038852 | 3.799672 | 8.037293 | 6.993731 | 0.91325848 | high |
| TCGA-HU-A4GY | 7.218634 | 4.899723 | 7.056556 | 5.735035 | 0.860253861 | low |
| TCGA-BR-8365 | 7.897868 | 5.830301 | 9.722782 | 6.891859 | 1.01151662 | high |
| TCGA-D7-5578 | 6.967779 | 4.362848 | 8.311446 | 5.565257 | 0.825770854 | low |
| TCGA-BR-8682 | 7.379331 | 5.486436 | 8.489189 | 4.904513 | 0.806692453 | low |
| TCGA-D7-8570 | 7.529907 | 4.386855 | 8.164186 | 6.531892 | 0.929728689 | high |
| TCGA-BR-6453 | 8.455176 | 5.146272 | 9.010433 | 6.650138 | 0.987539825 | high |
| TCGA-HU-A4GP | 5.565557 | 3.845319 | 8.059116 | 6.930692 | 0.895057245 | high |
| TCGA-HU-A4H8 | 4.805591 | 3.537771 | 6.827953 | 4.262238 | 0.622667742 | low |
| TCGA-VQ-A8E3 | 5.216167 | 3.984874 | 6.317413 | 6.557788 | 0.852457172 | low |
| TCGA-KB-A93H | 5.13202 | 4.297226 | 7.079381 | 4.837684 | 0.703635029 | low |
| TCGA-CG-4476 | 8.29227 | 5.918597 | 8.88444 | 5.409668 | 0.890192265 | low |
| TCGA-BR-8297 | 6.826949 | 5.18072 | 9.216606 | 5.97788 | 0.880877431 | low |
| TCGA-HU-A4GF | 6.777603 | 4.118356 | 8.001281 | 6.152524 | 0.866690711 | low |
| TCGA-HU-A4GT | 5.354824 | 3.668079 | 8.856259 | 6.045699 | 0.805637218 | low |
| TCGA-VQ-A91A | 6.597507 | 5.604283 | 8.567777 | 8.116807 | 1.077224184 | high |
| TCGA-VQ-A8PX | 5.40784 | 3.995677 | 7.850645 | 4.965797 | 0.716592424 | low |
| TCGA-IN-A7NR | 8.125614 | 3.825608 | 7.625219 | 5.892247 | 0.874272854 | low |
| TCGA-HU-A4G8 | 5.691097 | 3.824116 | 7.29182 | 4.81034 | 0.705653867 | low |
| TCGA-CG-5724 | 6.826513 | 5.572914 | 7.135506 | 7.142406 | 0.993136391 | high |
| TCGA-BR-4201 | 8.018707 | 7.994783 | 9.424253 | 7.841917 | 1.155181296 | high |
| TCGA-VQ-A91K | 5.717564 | 4.582124 | 6.898578 | 4.832867 | 0.727108216 | low |
| TCGA-IN-A6RL | 7.662349 | 4.406713 | 8.417647 | 6.260588 | 0.909907165 | high |
| TCGA-MX-A5UJ | 7.315879 | 4.972057 | 9.742614 | 6.265935 | 0.916463012 | high |
| TCGA-BR-8286 | 6.375428 | 5.196668 | 6.997526 | 5.404342 | 0.813432732 | low |
| TCGA-R5-A7ZF | 5.285987 | 3.77061 | 6.114346 | 7.254055 | 0.911651534 | high |
| TCGA-D7-6815 | 5.712057 | 4.289821 | 7.756813 | 5.051821 | 0.740480201 | low |
| TCGA-CD-8525 | 6.089277 | 5.975077 | 6.970928 | 5.196441 | 0.806046273 | low |
| TCGA-CG-5719 | 7.883092 | 7.000655 | 8.083776 | 7.057882 | 1.053490042 | high |
| TCGA-RD-A8NB | 6.276443 | 4.671302 | 8.05689 | 6.041772 | 0.856260269 | low |
| TCGA-BR-A4IV | 8.708856 | 7.700728 | 9.593502 | 7.663423 | 1.151817488 | high |
| TCGA-HF-A5NB | 5.077411 | 3.445043 | 7.496061 | 5.260343 | 0.719213857 | low |
| TCGA-FP-8099 | 5.970656 | 4.84227 | 6.854432 | 5.265066 | 0.77998224 | low |
| TCGA-VQ-A94T | 5.574791 | 4.491476 | 8.339497 | 5.332964 | 0.767785746 | low |
| TCGA-CD-8533 | 5.426157 | 4.514464 | 9.374805 | 5.194524 | 0.752963816 | low |
| TCGA-CD-8530 | 6.823064 | 6.130473 | 7.79186 | 7.931419 | 1.079280605 | high |
| TCGA-VQ-AA68 | 5.03524 | 3.617948 | 7.253419 | 7.226599 | 0.899591157 | high |
| TCGA-BR-8677 | 10.057817 | 6.113685 | 7.584114 | 7.938205 | 1.1727784 | high |
| TCGA-HU-A4HB | 7.039716 | 4.03367 | 9.739509 | 5.221698 | 0.790425642 | low |
| TCGA-BR-7707 | 5.832808 | 5.452002 | 6.939281 | 6.368905 | 0.891215112 | low |
| TCGA-VQ-A8P8 | 6.41457 | 4.082509 | 7.994205 | 5.834657 | 0.82656761 | low |
| TCGA-BR-8384 | 7.314992 | 6.184668 | 9.746661 | 6.903821 | 1.004733839 | high |
| TCGA-RD-A8N1 | 6.816821 | 4.935516 | 7.990701 | 7.298106 | 0.991937105 | high |
| TCGA-VQ-A8DT | 5.553256 | 3.717886 | 6.770049 | 4.992017 | 0.714677807 | low |
| TCGA-BR-8487 | 5.761931 | 4.851805 | 7.784894 | 5.678397 | 0.812763432 | low |
| TCGA-CD-8526 | 6.766264 | 5.696563 | 7.829967 | 6.195434 | 0.909950636 | high |
| TCGA-HU-A4H6 | 6.762517 | 4.158614 | 7.952821 | 6.223991 | 0.87366107 | low |
| TCGA-BR-7957 | 7.631616 | 6.887206 | 9.535337 | 7.248985 | 1.062558266 | high |
| TCGA-BR-6452 | 6.418898 | 4.199201 | 6.693138 | 6.204329 | 0.861270711 | low |
| TCGA-BR-8592 | 7.646095 | 5.844604 | 9.162088 | 6.380069 | 0.957623769 | high |
| TCGA-D7-8573 | 4.362904 | 3.748032 | 5.976902 | 4.360584 | 0.622912981 | low |
| TCGA-D7-A6F0 | 6.985096 | 6.015715 | 7.669359 | 4.501892 | 0.771219811 | low |
| TCGA-FP-A8CX | 8.508047 | 4.177396 | 7.93279 | 6.606518 | 0.959153788 | high |
| TCGA-ZQ-A9CR | 6.975414 | 4.786689 | 9.413333 | 9.848719 | 1.22499399 | high |
| TCGA-HU-8238 | 6.080408 | 4.055842 | 8.498878 | 6.617366 | 0.887582594 | low |
| TCGA-VQ-AA6A | 7.041081 | 3.91161 | 6.503765 | 5.852469 | 0.839965412 | low |
| TCGA-BR-8081 | 8.189983 | 4.993277 | 8.46055 | 7.058764 | 1.012150555 | high |
| TCGA-VQ-AA6G | 5.390511 | 3.608135 | 8.283232 | 7.126306 | 0.901955572 | high |
| TCGA-BR-8686 | 7.481007 | 4.598009 | 9.63751 | 7.068372 | 0.984089906 | high |
| TCGA-VQ-A94O | 8.571929 | 3.883102 | 6.81222 | 6.289424 | 0.923407998 | high |
| TCGA-FP-8211 | 5.62547 | 3.846746 | 7.463136 | 5.143634 | 0.734654842 | low |
| TCGA-BR-7197 | 5.562497 | 4.100115 | 6.558732 | 5.081569 | 0.732420539 | low |
| TCGA-FP-7735 | 7.278819 | 4.198082 | 6.804474 | 7.172346 | 0.973682427 | high |
| TCGA-D7-A6EY | 6.715585 | 4.447819 | 7.43489 | 5.020067 | 0.770212133 | low |
| TCGA-BR-8678 | 5.949467 | 4.272716 | 6.791011 | 6.12399 | 0.842427114 | low |
| TCGA-BR-8680 | 4.483375 | 4.478144 | 6.350624 | 4.441497 | 0.652688463 | low |
| TCGA-CG-4462 | 7.821097 | 7.241442 | 10.02659 | 7.802427 | 1.127643945 | high |
| TCGA-RD-A8N5 | 6.291285 | 5.518074 | 9.283596 | 8.690899 | 1.118987875 | high |
| TCGA-BR-8295 | 6.566105 | 4.990451 | 7.661045 | 5.780215 | 0.848566328 | low |
| TCGA-D7-8578 | 7.559121 | 5.803341 | 8.987612 | 8.056121 | 1.105171396 | high |
| TCGA-R5-A7ZI | 7.070919 | 3.676507 | 6.182827 | 4.83336 | 0.742421665 | low |
| TCGA-FP-8209 | 7.884959 | 5.698144 | 10.279356 | 6.802533 | 1.000483546 | high |
| TCGA-D7-8574 | 6.496238 | 5.683459 | 6.987391 | 5.387908 | 0.827751187 | low |
| TCGA-D7-5577 | 6.985443 | 3.608925 | 6.932189 | 7.138895 | 0.947434471 | high |
| TCGA-R5-A7O7 | 5.96759 | 4.382574 | 6.831607 | 7.485625 | 0.968739498 | high |
| TCGA-RD-A7BS | 9.306358 | 4.628016 | 9.265159 | 7.997785 | 1.121083241 | high |
| TCGA-BR-A4J5 | 6.634956 | 6.699939 | 9.079018 | 6.161586 | 0.930176646 | high |
| TCGA-BR-6709 | 9.265111 | 6.101045 | 11.182201 | 11.117826 | 1.441493005 | high |
| TCGA-MX-A666 | 8.095162 | 3.873956 | 7.697162 | 5.013362 | 0.795351163 | low |
| TCGA-BR-A44T | 6.808051 | 5.167294 | 9.157761 | 6.740407 | 0.948765972 | high |
| TCGA-CD-5813 | 9.730095 | 8.14153 | 7.78341 | 7.058169 | 1.135425072 | high |
| TCGA-CG-4444 | 7.023084 | 5.165271 | 8.409707 | 5.925453 | 0.880337937 | low |
| TCGA-CD-5798 | 7.628951 | 5.82338 | 8.390344 | 6.81264 | 0.99460909 | high |
| TCGA-BR-7851 | 6.554602 | 4.679142 | 7.677928 | 6.544095 | 0.909353773 | high |
| TCGA-BR-8591 | 6.103102 | 4.551342 | 7.710352 | 6.716201 | 0.908640569 | high |
| TCGA-D7-A6EV | 5.635972 | 3.917229 | 7.243202 | 5.760165 | 0.792117184 | low |
| TCGA-R5-A805 | 7.421041 | 4.130831 | 8.565867 | 6.84369 | 0.94879579 | high |
| TCGA-VQ-AA6F | 5.467294 | 4.204118 | 7.057036 | 4.352383 | 0.66712706 | low |
| TCGA-BR-4256 | 8.215126 | 7.24703 | 10.452143 | 7.214578 | 1.086678639 | high |
| TCGA-BR-A4J6 | 6.451568 | 5.798302 | 7.031836 | 4.873246 | 0.782952562 | low |
| TCGA-CG-4443 | 6.04205 | 3.639381 | 5.716072 | 4.521666 | 0.682938644 | low |
| TCGA-CG-5718 | 6.582038 | 3.894332 | 7.414571 | 6.460425 | 0.88237326 | low |
| TCGA-BR-6455 | 6.516761 | 4.695525 | 7.614159 | 6.962931 | 0.946407501 | high |
| TCGA-BR-4363 | 7.220887 | 6.700105 | 8.160384 | 7.662907 | 1.08145954 | high |
| TCGA-VQ-A8P5 | 5.485183 | 5.59803 | 7.685505 | 6.781317 | 0.923100522 | high |
| TCGA-BR-A4PF | 5.787796 | 4.336473 | 7.923152 | 4.675555 | 0.710100819 | low |
| TCGA-VQ-A94R | 6.284197 | 4.981112 | 6.723791 | 4.749856 | 0.745869252 | low |
| TCGA-BR-4369 | 7.558149 | 4.328954 | 7.804669 | 5.497194 | 0.835164036 | low |
| TCGA-D7-6519 | 7.041381 | 4.97046 | 6.925382 | 6.121463 | 0.891632361 | low |
| TCGA-VQ-A91U | 5.835443 | 3.619294 | 7.430957 | 5.657764 | 0.781354515 | low |
| TCGA-VQ-A925 | 6.612823 | 4.544625 | 6.963698 | 5.278851 | 0.792420733 | low |
| TCGA-BR-8372 | 6.293899 | 4.283133 | 9.75209 | 5.358223 | 0.787505919 | low |
| TCGA-F1-6875 | 6.208553 | 4.154905 | 6.707865 | 7.364657 | 0.958858723 | high |
| TCGA-FP-7916 | 6.401959 | 5.260982 | 7.919258 | 5.009091 | 0.781381986 | low |
| TCGA-VQ-AA6D | 6.29148 | 6.399836 | 7.040513 | 5.266863 | 0.829100135 | low |
| TCGA-BR-8589 | 5.923436 | 3.701242 | 8.059155 | 4.741682 | 0.704102213 | low |
| TCGA-3M-AB46 | 4.730424 | 3.983493 | 7.712426 | 5.709927 | 0.763691302 | low |
| TCGA-CG-5734 | 7.719205 | 3.816054 | 9.164113 | 7.52474 | 1.011760342 | high |
| TCGA-BR-4294 | 7.616526 | 6.761724 | 9.090522 | 5.66727 | 0.915519819 | high |
| TCGA-VQ-A924 | 5.855888 | 4.283359 | 7.869382 | 6.254258 | 0.853207508 | low |
| TCGA-BR-8683 | 6.353321 | 4.241674 | 9.354382 | 7.074475 | 0.942612879 | high |
| TCGA-VQ-A8PK | 6.555835 | 4.468561 | 6.953687 | 7.949101 | 1.029951067 | high |
| TCGA-BR-A4J4 | 5.504198 | 4.475552 | 9.893696 | 7.439487 | 0.957652646 | high |
| TCGA-R5-A7ZE | 10.101396 | 3.755943 | 6.697058 | 6.761975 | 1.006956201 | high |
| TCGA-D7-6522 | 9.749005 | 5.743808 | 7.551486 | 6.391163 | 1.014743375 | high |
| TCGA-D7-6822 | 7.157069 | 4.248627 | 6.839978 | 5.015719 | 0.776748286 | low |
| TCGA-BR-6803 | 8.62305 | 6.04856 | 8.974277 | 5.328984 | 0.895887692 | high |
| TCGA-BR-8483 | 6.143634 | 4.841548 | 7.493419 | 4.9493 | 0.757318217 | low |
| TCGA-BR-6563 | 7.677903 | 7.289159 | 8.194115 | 5.996205 | 0.959137196 | high |
| TCGA-VQ-A8E7 | 6.273779 | 3.901833 | 7.911811 | 7.567467 | 0.974279026 | high |
| TCGA-CG-4436 | 6.105843 | 3.617157 | 7.897507 | 6.219719 | 0.840496934 | low |
| TCGA-D7-6520 | 6.201791 | 5.959757 | 7.448405 | 5.575936 | 0.843825954 | low |
| TCGA-BR-4368 | 7.112014 | 5.309719 | 9.037683 | 6.700399 | 0.957390532 | high |
| TCGA-BR-8676 | 5.29959 | 5.271421 | 7.163249 | 5.701434 | 0.811248232 | low |
| TCGA-BR-6457 | 8.657049 | 7.382659 | 8.471563 | 7.148099 | 1.094221155 | high |
| TCGA-D7-A747 | 6.806573 | 5.357862 | 9.137113 | 4.06135 | 0.711605523 | low |
| TCGA-BR-A44U | 6.305298 | 5.080452 | 9.034798 | 5.696929 | 0.837632153 | low |
| TCGA-KB-A93G | 7.368141 | 6.2222 | 9.432974 | 10.212843 | 1.305595704 | high |
| TCGA-BR-8367 | 7.100152 | 5.802058 | 8.801762 | 5.945042 | 0.900982906 | high |
| TCGA-D7-A6F2 | 5.642154 | 4.1502 | 7.73663 | 5.395678 | 0.765946202 | low |
| TCGA-D7-6521 | 7.551438 | 4.580251 | 7.488316 | 6.869306 | 0.964801974 | high |
| TCGA-BR-6454 | 6.897538 | 6.432363 | 6.846567 | 5.893754 | 0.903803455 | high |
| TCGA-CG-4466 | 6.397035 | 5.289199 | 7.941902 | 6.192007 | 0.888799042 | low |
| TCGA-CD-8524 | 7.627189 | 6.106707 | 7.088561 | 7.203771 | 1.035290271 | high |
| TCGA-BR-7723 | 7.925044 | 4.965428 | 7.580308 | 6.735415 | 0.973394394 | high |
| TCGA-CD-A48C | 7.456936 | 6.710302 | 8.082806 | 7.430483 | 1.067455073 | high |
| TCGA-HU-A4GX | 6.235925 | 3.775329 | 7.967857 | 5.934756 | 0.822627324 | low |
| TCGA-BR-8382 | 6.133135 | 5.391379 | 7.51084 | 5.613516 | 0.830931519 | low |
| TCGA-IN-AB1V | 8.333351 | 3.70858 | 8.623196 | 3.523911 | 0.664812686 | low |
| TCGA-CD-8532 | 6.150869 | 4.356604 | 8.394053 | 6.412114 | 0.878558228 | low |
| TCGA-IN-8663 | 5.070771 | 3.521536 | 6.615949 | 9.390981 | 1.092757564 | high |
| TCGA-VQ-A91D | 6.442163 | 4.410026 | 7.623422 | 8.369423 | 1.064038888 | high |
| TCGA-KB-A6F7 | 7.396723 | 6.187243 | 7.852893 | 6.053941 | 0.927865143 | high |
| TCGA-VQ-A8PQ | 7.105093 | 5.540569 | 8.342299 | 6.133929 | 0.910943457 | high |
| TCGA-B7-A5TN | 6.190949 | 5.348142 | 7.682966 | 7.656022 | 1.016176159 | high |
| TCGA-VQ-A8E0 | 6.585783 | 4.261696 | 7.07029 | 5.704304 | 0.823038807 | low |
| TCGA-CD-5803 | 7.403693 | 6.80516 | 8.436607 | 7.988271 | 1.119160096 | high |
| TCGA-BR-8371 | 7.698535 | 5.585838 | 10.079698 | 4.559611 | 0.789446757 | low |
| TCGA-VQ-A8PU | 5.472171 | 5.819393 | 5.902832 | 4.650028 | 0.733469662 | low |
| TCGA-HU-A4G2 | 6.246141 | 4.451034 | 6.635423 | 6.069813 | 0.850421876 | low |
| TCGA-B7-A5TK | 6.774214 | 5.061255 | 8.437907 | 6.811369 | 0.950539167 | high |
| TCGA-SW-A7EB | 7.597716 | 4.550129 | 8.461478 | 5.894179 | 0.87864024 | low |
| TCGA-BR-8690 | 7.337204 | 5.648163 | 8.556909 | 5.54489 | 0.867483007 | low |
| TCGA-BR-4357 | 7.54729 | 5.893828 | 9.058822 | 6.896425 | 1.002497382 | high |
| TCGA-CG-4440 | 7.851919 | 4.581076 | 8.795935 | 8.234574 | 1.098562416 | high |
| TCGA-BR-6802 | 6.379736 | 4.554102 | 7.311658 | 6.797469 | 0.923514377 | high |
| TCGA-BR-4370 | 7.545988 | 6.696638 | 8.668495 | 7.035267 | 1.034789168 | high |
| TCGA-VQ-A8E2 | 7.541314 | 4.910969 | 8.26975 | 7.773752 | 1.055603884 | high |
| TCGA-D7-6818 | 8.588958 | 6.649034 | 8.095507 | 6.813164 | 1.042931465 | high |
| TCGA-RD-A8N9 | 7.70383 | 5.444159 | 7.463244 | 5.942722 | 0.907374289 | high |
| TCGA-D7-6525 | 6.586804 | 6.153475 | 7.746043 | 9.11922 | 1.180221174 | high |
| TCGA-HU-A4GU | 4.741803 | 3.846496 | 5.623742 | 5.277484 | 0.718685116 | low |
| TCGA-BR-8059 | 7.642404 | 5.207927 | 8.183017 | 7.013852 | 0.997310315 | high |
| TCGA-F1-6177 | 5.851641 | 3.712149 | 7.412229 | 6.823922 | 0.889449625 | low |
| TCGA-CD-8528 | 5.194533 | 5.064171 | 8.320923 | 5.068605 | 0.747382666 | low |
| TCGA-BR-6710 | 9.296682 | 4.22769 | 7.677899 | 6.047654 | 0.93243585 | high |
| TCGA-BR-8291 | 7.393048 | 6.350941 | 7.97427 | 8.220665 | 1.127716397 | high |
| TCGA-D7-6526 | 5.402011 | 3.819615 | 8.241481 | 6.468479 | 0.848184151 | low |
| TCGA-VQ-A91X | 7.221058 | 4.420665 | 5.977446 | 3.49431 | 0.644409733 | low |
| TCGA-BR-6801 | 6.033231 | 4.351183 | 7.307435 | 5.310464 | 0.774074065 | low |
| TCGA-VQ-A91V | 6.823563 | 5.196518 | 6.653165 | 5.011358 | 0.790444286 | low |
| TCGA-VQ-A8PM | 8.056967 | 4.051087 | 8.44343 | 7.605367 | 1.033789096 | high |
| TCGA-CD-8529 | 7.240721 | 5.161931 | 8.640063 | 7.553666 | 1.033885325 | high |
| TCGA-CG-4460 | 7.526203 | 6.911037 | 7.866429 | 5.340193 | 0.885499002 | low |
| TCGA-BR-A4J9 | 6.616649 | 5.189977 | 9.680801 | 5.834845 | 0.862737987 | low |
| TCGA-B7-A5TJ | 5.489659 | 4.070333 | 7.224822 | 5.954772 | 0.809306805 | low |
| TCGA-IN-A6RS | 6.748164 | 3.961043 | 6.856188 | 7.548016 | 0.986321632 | high |
| TCGA-D7-A6EZ | 6.626148 | 5.135961 | 6.559507 | 6.194998 | 0.889953556 | low |
| TCGA-BR-7717 | 6.422899 | 4.893043 | 8.124544 | 7.3875 | 0.987717606 | high |
| TCGA-CD-8527 | 7.092135 | 4.764559 | 6.941871 | 4.825376 | 0.77087782 | low |
| TCGA-IN-A6RR | 6.733094 | 4.241629 | 8.310174 | 6.724457 | 0.920584264 | high |
| TCGA-BR-7959 | 7.145266 | 6.502975 | 8.720587 | 8.533103 | 1.153615241 | high |
| TCGA-RD-A8MV | 6.262048 | 3.696786 | 9.244072 | 6.460144 | 0.870559265 | low |
| TCGA-BR-8484 | 6.736247 | 4.307437 | 8.722967 | 6.268925 | 0.881764724 | low |
| TCGA-HU-A4GC | 7.491052 | 4.842565 | 8.414982 | 6.00315 | 0.892732823 | low |
| TCGA-CD-5799 | 5.950016 | 5.206712 | 6.767001 | 7.608262 | 1.00007669 | high |
| TCGA-HU-A4H0 | 5.666794 | 3.417398 | 8.165722 | 5.079252 | 0.720118427 | low |
| TCGA-FP-A4BF | 7.523628 | 6.156236 | 10.02513 | 7.671785 | 1.079772024 | high |
| TCGA-BR-8380 | 7.27334 | 5.687665 | 9.457701 | 7.692355 | 1.061762432 | high |
| TCGA-VQ-A91Q | 5.461036 | 4.740586 | 7.934444 | 8.205027 | 1.029593885 | high |
| TCGA-FP-7829 | 6.818875 | 4.470225 | 6.504216 | 5.154362 | 0.784638013 | low |
| TCGA-HF-7134 | 5.693096 | 3.527801 | 7.140532 | 7.395522 | 0.931444132 | high |
| TCGA-CG-4441 | 6.26633 | 5.320836 | 7.016773 | 6.293122 | 0.893699838 | low |
| TCGA-FP-8631 | 7.326656 | 4.751625 | 8.321338 | 7.116831 | 0.986111299 | high |
| TCGA-BR-4279 | 9.556784 | 8.464797 | 9.58494 | 7.890929 | 1.21622079 | high |
| TCGA-IN-A6RJ | 8.225982 | 3.489469 | 6.465593 | 3.919767 | 0.688993077 | low |
| TCGA-VQ-AA6J | 6.574235 | 3.760151 | 6.447384 | 5.832949 | 0.820786107 | low |
| TCGA-CD-5800 | 7.841533 | 4.228565 | 6.92211 | 5.956777 | 0.881130337 | low |
| TCGA-CG-4442 | 6.234386 | 5.220915 | 9.534252 | 6.407341 | 0.903958453 | high |
| TCGA-CG-4465 | 6.852009 | 5.13084 | 9.134642 | 5.418894 | 0.829754145 | low |
| TCGA-CG-5720 | 7.568226 | 4.305135 | 9.305272 | 7.585483 | 1.025446284 | high |
| TCGA-D7-6524 | 6.743518 | 4.460068 | 8.531582 | 7.573449 | 1.003375587 | high |
| TCGA-CD-A486 | 6.82238 | 6.147331 | 7.423417 | 7.615101 | 1.050627327 | high |
| TCGA-CG-5716 | 7.604487 | 3.764816 | 6.933616 | 4.303559 | 0.713267901 | low |
| TCGA-RD-A7BT | 5.426858 | 3.604588 | 7.16509 | 6.150893 | 0.813330432 | low |
| TCGA-IN-A7NT | 7.471908 | 5.281314 | 8.16311 | 9.236771 | 1.194932778 | high |
| TCGA-BR-8296 | 6.095805 | 4.15139 | 8.221286 | 7.283957 | 0.950263442 | high |
| TCGA-BR-4187 | 8.48323 | 7.206466 | 10.141708 | 6.636375 | 1.04077943 | high |
| TCGA-SW-A7EA | 5.373026 | 5.026266 | 6.713364 | 5.282331 | 0.768716682 | low |
| TCGA-BR-6705 | 10.226554 | 7.988087 | 7.912813 | 7.902555 | 1.222326266 | high |
| TCGA-BR-A4QL | 4.891334 | 3.772596 | 7.698436 | 3.941853 | 0.603336874 | low |
| TCGA-BR-7716 | 7.598416 | 4.346687 | 7.881416 | 6.272008 | 0.906845534 | high |
| TCGA-HU-8608 | 6.595204 | 3.53774 | 7.081639 | 6.210372 | 0.850700169 | low |
| TCGA-F1-A72C | 6.37733 | 5.572302 | 8.452108 | 6.26946 | 0.903076805 | high |
| TCGA-VQ-A8P3 | 6.258188 | 5.937002 | 7.403319 | 5.066876 | 0.798853859 | low |
| TCGA-IP-7968 | 7.089836 | 4.35244 | 8.165608 | 7.463372 | 1.000237738 | high |
| TCGA-BR-4280 | 5.222436 | 3.642443 | 7.737248 | 5.434805 | 0.744485144 | low |
| TCGA-IN-7808 | 7.035361 | 3.898488 | 8.810375 | 5.156663 | 0.779751495 | low |
| TCGA-D7-8572 | 6.691331 | 6.016457 | 7.917146 | 6.384455 | 0.933065028 | high |
| TCGA-BR-8366 | 7.804049 | 4.785557 | 7.695101 | 7.231255 | 1.010269362 | high |
| TCGA-CD-A48A | 6.556004 | 4.289221 | 7.322338 | 4.459983 | 0.710854575 | low |
| TCGA-VQ-A8PC | 7.094348 | 4.130571 | 10.133371 | 5.941095 | 0.859950516 | low |
| TCGA-D7-6528 | 5.349969 | 4.198038 | 5.989457 | 6.409299 | 0.847872716 | low |
| TCGA-BR-6707 | 6.589693 | 4.581709 | 6.993482 | 5.2 | 0.785610243 | low |
| TCGA-BR-8368 | 5.37493 | 4.216399 | 5.748194 | 5.554371 | 0.771535799 | low |
| TCGA-VQ-A92D | 5.169908 | 4.145303 | 7.541448 | 5.929801 | 0.800125578 | low |
| TCGA-HU-A4H2 | 5.762728 | 3.957113 | 7.036189 | 5.793151 | 0.79949319 | low |
| TCGA-BR-8361 | 5.382397 | 3.67727 | 7.320901 | 6.347267 | 0.831826107 | low |
| TCGA-VQ-A91Z | 8.006704 | 3.529465 | 5.900254 | 4.920184 | 0.773233471 | low |
| TCGA-D7-6527 | 7.523933 | 5.684417 | 7.01434 | 4.840756 | 0.808139217 | low |
| TCGA-RD-A8MW | 7.607888 | 5.294118 | 8.618179 | 8.462183 | 1.129861746 | high |
| TCGA-D7-A6EX | 5.537795 | 5.186478 | 8.170927 | 5.874373 | 0.832966444 | low |
| TCGA-VQ-A927 | 6.553385 | 3.809833 | 8.502625 | 6.30709 | 0.867028418 | low |
| TCGA-BR-4257 | 7.542558 | 6.785306 | 8.460531 | 6.786441 | 1.014184957 | high |
| TCGA-CG-4475 | 6.648659 | 5.935116 | 8.90003 | 8.253256 | 1.099847096 | high |
| TCGA-CD-5801 | 6.511127 | 3.709296 | 8.581676 | 4.41799 | 0.692786199 | low |
| TCGA-VQ-A8PP | 6.112415 | 5.192375 | 7.300422 | 7.104707 | 0.959662551 | high |
| TCGA-RD-A7C1 | 6.819666 | 4.714561 | 7.090473 | 6.383042 | 0.902583572 | high |
| TCGA-HU-8604 | 6.34329 | 5.269359 | 7.74954 | 8.096795 | 1.058480521 | high |
| TCGA-BR-6456 | 7.556699 | 5.320216 | 8.505463 | 8.492777 | 1.131651676 | high |
| TCGA-BR-A4CR | 5.091184 | 5.029661 | 9.574565 | 6.429769 | 0.868121416 | low |
| TCGA-BR-7901 | 7.691507 | 5.352574 | 8.717673 | 7.870781 | 1.080492566 | high |
| TCGA-BR-4371 | 4.81375 | 3.987022 | 7.224608 | 4.124988 | 0.622416415 | low |
| TCGA-MX-A663 | 8.535705 | 6.171685 | 8.851751 | 8.323525 | 1.166712113 | high |
| TCGA-VQ-A8PF | 6.881189 | 3.945319 | 7.217985 | 7.969611 | 1.028329921 | high |
| TCGA-BR-7715 | 6.734956 | 5.481892 | 7.651285 | 6.269803 | 0.910085642 | high |
| TCGA-BR-4367 | 7.116069 | 5.085127 | 8.036559 | 6.221964 | 0.907270428 | high |
| TCGA-HF-7133 | 7.039557 | 5.450931 | 7.712656 | 4.79557 | 0.785077724 | low |
| TCGA-D7-8575 | 6.808834 | 3.701217 | 8.086157 | 6.556387 | 0.893619016 | low |
| TCGA-BR-8077 | 5.636381 | 4.489475 | 8.528071 | 6.706889 | 0.893834093 | low |
| TCGA-CD-8531 | 5.920689 | 4.307481 | 9.805982 | 6.161836 | 0.849961447 | low |
| TCGA-IN-7806 | 7.362183 | 3.620334 | 9.450484 | 4.694992 | 0.741343382 | low |
| TCGA-BR-8485 | 6.892233 | 4.432335 | 7.396847 | 7.255532 | 0.976737497 | high |
| TCGA-BR-4361 | 7.046916 | 5.675789 | 9.684123 | 7.40692 | 1.029442234 | high |
| TCGA-IN-A7NU | 6.951854 | 4.612524 | 7.503908 | 7.815638 | 1.03374398 | high |
| TCGA-HU-A4H5 | 6.271208 | 3.903737 | 6.354188 | 5.339047 | 0.770928782 | low |
| TCGA-RD-A7BW | 7.979034 | 6.051302 | 9.070969 | 8.092311 | 1.126976951 | high |
| TCGA-D7-8579 | 6.320755 | 4.95918 | 9.288226 | 5.477183 | 0.815509449 | low |
| TCGA-BR-8486 | 6.223699 | 4.136688 | 8.803729 | 6.841434 | 0.91441897 | high |
| TCGA-RD-A8N6 | 7.15708 | 5.157596 | 9.167562 | 5.892138 | 0.882035245 | low |
| TCGA-D7-A4YX | 5.437823 | 3.782279 | 6.783112 | 4.479344 | 0.666692162 | low |
| TCGA-BR-A4CS | 5.795609 | 6.173164 | 7.982108 | 6.61093 | 0.931653473 | high |
| TCGA-BR-4191 | 7.361073 | 5.842624 | 8.78758 | 6.847233 | 0.991005573 | high |
| TCGA-B7-A5TI | 6.814133 | 4.917953 | 8.726331 | 7.349294 | 0.997029941 | high |
| TCGA-CG-5722 | 7.422031 | 6.110406 | 8.175831 | 6.577389 | 0.974354761 | high |
| TCGA-CG-5726 | 5.462875 | 3.596675 | 6.071068 | 4.526222 | 0.665991191 | low |
| TCGA-D7-A748 | 9.549194 | 7.27403 | 8.315766 | 6.566207 | 1.064530072 | high |
| TCGA-BR-8373 | 6.020863 | 4.640079 | 7.058433 | 6.615885 | 0.898568821 | high |
| TCGA-BR-A4J7 | 6.839574 | 6.396533 | 8.965311 | 4.133242 | 0.745108978 | low |
| TCGA-HU-A4GH | 6.205771 | 4.096573 | 7.672083 | 4.966948 | 0.742095371 | low |
| TCGA-CG-4437 | 6.954451 | 5.004323 | 7.503939 | 6.738865 | 0.946505332 | high |
| TCGA-CG-4469 | 6.44474 | 4.01335 | 7.361501 | 5.672472 | 0.810191103 | low |
| TCGA-VQ-A8PE | 7.259026 | 4.694698 | 7.241228 | 7.086434 | 0.97851102 | high |
| TCGA-CD-8534 | 6.427219 | 5.030836 | 7.591258 | 3.953232 | 0.680503269 | low |
| TCGA-VQ-A94P | 7.962987 | 6.479242 | 10.472671 | 5.982075 | 0.948686108 | high |
| TCGA-BR-8364 | 6.822311 | 7.414992 | 8.716774 | 8.576192 | 1.171240185 | high |
| TCGA-KB-A93J | 7.006449 | 3.785589 | 7.894958 | 5.020985 | 0.762570434 | low |
| TCGA-BR-8080 | 7.251454 | 6.740951 | 8.164423 | 7.313365 | 1.051820302 | high |
| TCGA-IN-8462 | 10.029445 | 5.011587 | 9.705272 | 7.30029 | 1.08932413 | high |
| TCGA-F1-6874 | 6.588635 | 3.800391 | 7.480231 | 8.823639 | 1.093668265 | high |
| TCGA-CD-A487 | 7.257237 | 5.197327 | 8.368205 | 4.948325 | 0.799635387 | low |
| TCGA-CG-4301 | 7.448579 | 4.94065 | 9.52088 | 6.750341 | 0.962949001 | high |
| TCGA-BR-8060 | 7.759422 | 9.075333 | 9.589052 | 8.108594 | 1.199331086 | high |
| TCGA-HU-A4HD | 6.596119 | 4.857946 | 9.048432 | 6.266839 | 0.891894818 | low |
| TCGA-BR-8369 | 9.117862 | 5.42578 | 7.926097 | 6.852184 | 1.030568209 | high |
| TCGA-BR-4267 | 5.824322 | 4.756316 | 7.821251 | 6.43922 | 0.880901504 | low |
| TCGA-BR-8284 | 7.057528 | 4.535419 | 8.38829 | 6.508111 | 0.917976749 | high |
| TCGA-VQ-A8PD | 6.723025 | 3.910057 | 7.48143 | 7.634524 | 0.992959277 | high |
| TCGA-VQ-A922 | 6.570129 | 4.748222 | 7.875564 | 7.039106 | 0.956516637 | high |
| TCGA-VQ-A8PB | 4.942125 | 4.167536 | 7.364576 | 6.117595 | 0.810816557 | low |
| TCGA-FP-8210 | 7.634284 | 6.420605 | 8.528289 | 5.808071 | 0.919354976 | high |
| TCGA-RD-A8N0 | 7.175067 | 5.550211 | 7.529697 | 6.005491 | 0.900517503 | high |
| TCGA-BR-8289 | 7.256419 | 6.054805 | 7.040583 | 5.719145 | 0.889125917 | low |
| TCGA-HU-A4GJ | 7.023838 | 3.885965 | 7.558923 | 5.662066 | 0.823048728 | low |
| TCGA-HU-A4H3 | 5.590804 | 4.366742 | 7.542986 | 6.532932 | 0.872371418 | low |
| TCGA-VQ-A8P2 | 5.013295 | 3.684512 | 6.118456 | 3.78523 | 0.588360734 | low |
| TCGA-BR-8687 | 7.016599 | 5.039719 | 7.804783 | 6.21791 | 0.902564283 | high |
| TCGA-BR-4253 | 7.267092 | 4.01648 | 8.385981 | 6.199357 | 0.883022684 | low |
| TCGA-BR-8381 | 6.601762 | 5.081436 | 7.229809 | 6.736336 | 0.937654412 | high |
| TCGA-FP-7998 | 6.963762 | 4.337262 | 8.339296 | 5.339562 | 0.804664865 | low |
| TCGA-VQ-A8DZ | 6.94487 | 4.205819 | 9.069436 | 5.176683 | 0.78706936 | low |
| TCGA-HU-A4GD | 4.785831 | 4.376369 | 6.959386 | 5.288123 | 0.736133646 | low |
| TCGA-VQ-A8DV | 9.454793 | 4.916653 | 5.832479 | 6.395294 | 0.983348329 | high |
| TCGA-CG-4306 | 6.800393 | 4.60684 | 9.202867 | 6.821564 | 0.941748453 | high |
| TCGA-VQ-A8PH | 5.71119 | 3.810107 | 7.510667 | 4.906781 | 0.714884462 | low |
| TCGA-R5-A7ZR | 5.323443 | 5.770132 | 6.354345 | 4.192243 | 0.687192202 | low |
| TCGA-BR-6564 | 7.947316 | 5.97208 | 8.799767 | 5.875289 | 0.923495737 | high |
| TCGA-IN-A6RN | 7.07259 | 4.810355 | 9.913813 | 6.640434 | 0.93937884 | high |
| TCGA-VQ-A928 | 6.088828 | 5.540948 | 7.272149 | 6.914159 | 0.950558287 | high |
| TCGA-BR-7958 | 6.746549 | 4.335611 | 8.335938 | 7.672818 | 1.00902199 | high |
| TCGA-CG-4305 | 7.32818 | 5.888987 | 7.730284 | 6.957428 | 0.999750561 | high |

# Appendix 6

**Clinical features for the TCGA cohort**

**Table 6. Clinical features for the TCGA cohort.**

| gene | lowMean | highMean | logFC | pValue | fdr |
| --- | --- | --- | --- | --- | --- |
| DPYSL3 | 7.411103213 | 8.476181119 | 1.065077906 | 1.67E-14 | 5.94E-13 |
| TAGLN | 8.444850684 | 9.543477811 | 1.098627126 | 3.31E-14 | 1.08E-12 |
| FAP | 4.728041069 | 5.750164314 | 1.022123245 | 8.21E-23 | 5.08E-20 |
| COL14A1 | 4.796776716 | 5.800591892 | 1.003815176 | 1.97E-17 | 1.49E-15 |
| PDLIM3 | 5.983867794 | 7.117728103 | 1.133860309 | 9.02E-18 | 7.47E-16 |
| COL1A1 | 9.847368112 | 11.0654518 | 1.218083688 | 8.65E-18 | 7.21E-16 |
| TIMP3 | 5.394423629 | 6.426390854 | 1.031967225 | 4.34E-20 | 8.06E-18 |
| GAS1 | 4.82045583 | 5.993159946 | 1.172704116 | 1.54E-19 | 2.30E-17 |
| VCAN | 6.626911193 | 7.68723747 | 1.060326277 | 1.38E-21 | 4.26E-19 |
| MFAP4 | 7.695767134 | 8.747198805 | 1.051431671 | 1.38E-10 | 2.01E-09 |
| COL6A3 | 8.265256042 | 9.284657297 | 1.019401255 | 4.46E-20 | 8.16E-18 |
| COL1A2 | 9.304848612 | 10.47173406 | 1.166885448 | 4.58E-20 | 8.27E-18 |
| PODN | 5.950192619 | 7.057544308 | 1.107351689 | 1.31E-15 | 6.15E-14 |
| CYP1B1 | 5.040938777 | 6.161359249 | 1.120420472 | 3.65E-14 | 1.18E-12 |
| HSPB6 | 5.690555757 | 6.729830119 | 1.039274362 | 4.36E-08 | 3.87E-07 |
| THBS4 | 5.233657958 | 6.734260351 | 1.500602393 | 3.60E-13 | 9.39E-12 |
| COL8A1 | 5.554902125 | 6.772158276 | 1.217256151 | 1.76E-20 | 3.52E-18 |
| AEBP1 | 7.471364273 | 8.681978946 | 1.210614673 | 9.25E-22 | 3.54E-19 |
| COL6A2 | 7.166663722 | 8.203708962 | 1.037045241 | 3.69E-19 | 4.65E-17 |
| CRISPLD2 | 6.581011935 | 7.603605432 | 1.022593497 | 7.95E-24 | 7.38E-21 |
| MGP | 8.091193873 | 9.474139351 | 1.382945479 | 8.00E-15 | 3.08E-13 |
| CRYAB | 5.116245492 | 6.132545238 | 1.016299746 | 9.66E-17 | 5.95E-15 |
| SULF1 | 7.238794007 | 8.444390184 | 1.205596177 | 8.51E-18 | 7.18E-16 |
| APOD | 6.737851626 | 7.805438827 | 1.067587201 | 1.06E-06 | 6.96E-06 |
| LUM | 8.842415827 | 9.959336514 | 1.116920687 | 1.37E-17 | 1.10E-15 |
| FNDC1 | 5.330305954 | 6.73842173 | 1.408115775 | 2.22E-18 | 2.22E-16 |
| CHRDL2 | 4.713330876 | 5.760308103 | 1.046977227 | 2.78E-11 | 4.69E-10 |
| CNN1 | 6.172515692 | 7.380624254 | 1.208108562 | 4.25E-10 | 5.62E-09 |
| TIMP2 | 7.585309023 | 8.606151281 | 1.020842258 | 3.89E-22 | 1.74E-19 |
| IGFBP4 | 8.631440687 | 9.63378227 | 1.002341583 | 8.83E-17 | 5.55E-15 |
| PTGIS | 4.650525746 | 5.695644205 | 1.045118459 | 2.01E-13 | 5.55E-12 |
| COL5A1 | 7.571782953 | 8.574068573 | 1.00228562 | 1.09E-19 | 1.71E-17 |
| POSTN | 6.571284533 | 7.609750124 | 1.038465592 | 9.39E-15 | 3.52E-13 |
| LOX | 4.800522082 | 5.824134508 | 1.023612426 | 1.96E-25 | 3.64E-22 |
| NNMT | 6.183167695 | 7.197468557 | 1.014300862 | 4.60E-23 | 3.32E-20 |
| LRRC32 | 6.323917883 | 7.325069849 | 1.001151966 | 6.97E-23 | 4.77E-20 |
| SRPX | 4.937151885 | 6.00111933 | 1.063967445 | 7.88E-19 | 8.83E-17 |
| CTHRC1 | 7.009917521 | 8.064897616 | 1.054980095 | 2.85E-15 | 1.22E-13 |
| MFAP5 | 5.337116248 | 6.547048703 | 1.209932454 | 2.57E-18 | 2.51E-16 |
| MYH11 | 7.609018594 | 8.709526578 | 1.100507985 | 2.16E-07 | 1.63E-06 |
| COL10A1 | 5.115410696 | 6.346019768 | 1.230609072 | 1.18E-12 | 2.74E-11 |
| THBS2 | 7.146400836 | 8.475852011 | 1.329451175 | 4.71E-19 | 5.57E-17 |
| MMP2 | 5.997938014 | 7.011478616 | 1.013540602 | 1.06E-22 | 6.01E-20 |
| EFEMP1 | 5.786255399 | 6.941424724 | 1.155169325 | 2.17E-17 | 1.61E-15 |
| FOSB | 5.993620579 | 7.284993357 | 1.291372778 | 1.64E-17 | 1.27E-15 |
| SFRP2 | 6.407648711 | 8.275258292 | 1.867609581 | 1.10E-17 | 8.92E-16 |
| DCN | 7.747065006 | 8.830838703 | 1.083773696 | 2.34E-18 | 2.30E-16 |
| SPON1 | 6.318389193 | 7.421618319 | 1.103229126 | 1.43E-12 | 3.25E-11 |
| BGN | 10.05107667 | 11.13662968 | 1.085553005 | 2.10E-22 | 1.01E-19 |
| ANGPTL2 | 6.712112813 | 7.831016162 | 1.118903349 | 7.59E-22 | 3.14E-19 |
| SPARC | 8.919929474 | 10.07716981 | 1.157240331 | 3.09E-25 | 5.03E-22 |
| SPOCK1 | 5.04888137 | 6.089783227 | 1.040901857 | 9.66E-17 | 5.95E-15 |
| IL6 | 4.186706835 | 5.206038962 | 1.019332127 | 3.21E-19 | 4.08E-17 |
| C1S | 7.159969006 | 8.197544324 | 1.037575318 | 3.96E-19 | 4.85E-17 |
| DUSP1 | 8.740983398 | 9.817277849 | 1.076294451 | 1.47E-22 | 7.98E-20 |
| FBLN2 | 5.64712638 | 6.760405632 | 1.113279253 | 8.60E-19 | 9.39E-17 |
| MYL9 | 7.193388888 | 8.293696378 | 1.100307491 | 1.78E-14 | 6.24E-13 |
| SFRP4 | 5.542507894 | 6.877478114 | 1.334970219 | 6.05E-13 | 1.51E-11 |
| CPE | 5.472158083 | 6.486031881 | 1.013873798 | 7.57E-16 | 3.80E-14 |
| SERPINF1 | 6.152139093 | 7.247799286 | 1.095660193 | 9.63E-20 | 1.54E-17 |
| SPARCL1 | 8.313919099 | 9.364051422 | 1.050132322 | 4.66E-14 | 1.49E-12 |
| FBLN1 | 6.747287641 | 7.891950924 | 1.144663283 | 2.57E-13 | 6.96E-12 |
| DES | 6.367282274 | 7.48985773 | 1.122575456 | 2.43E-05 | 0.000113068 |
| TNC | 6.067411434 | 7.112963627 | 1.045552193 | 3.41E-15 | 1.42E-13 |
| COL3A1 | 10.00304261 | 11.12341782 | 1.12037521 | 2.17E-20 | 4.20E-18 |
| SFRP1 | 4.236003125 | 5.276029341 | 1.040026216 | 7.65E-14 | 2.36E-12 |
| THBS1 | 7.239800854 | 8.797572524 | 1.557771671 | 7.35E-36 | 4.78E-32 |
| SERPINE1 | 5.400454038 | 7.218126238 | 1.8176722 | 5.71E-54 | 7.43E-50 |
| GREM1 | 6.49171786 | 7.832525243 | 1.340807384 | 4.70E-16 | 2.47E-14 |

# Appendix 7

## **GO and KEGG enrichment analysis**

**Table 7a. GO enrichment analysis.**

| ONTOLOGY | Description | BgRatio | pvalue | qvalue |
| --- | --- | --- | --- | --- |
| BP | extracellular matrix organization | 393/18862 | 2.42E-42 | 1.62E-39 |
| BP | extracellular structure organization | 394/18862 | 2.68E-42 | 1.62E-39 |
| BP | external encapsulating structure organization | 396/18862 | 3.28E-42 | 1.62E-39 |
| BP | collagen fibril organization | 54/18862 | 5.84E-14 | 2.16E-11 |
| BP | regulation of cell-substrate adhesion | 218/18862 | 1.51E-12 | 4.47E-10 |
| BP | regulation of angiogenesis | 335/18862 | 3.12E-12 | 7.70E-10 |
| BP | regulation of vasculature development | 341/18862 | 4.09E-12 | 8.64E-10 |
| BP | negative regulation of cell motility | 345/18862 | 4.87E-12 | 9.01E-10 |
| BP | negative regulation of cellular component movement | 352/18862 | 6.60E-12 | 1.08E-09 |
| BP | cell-substrate adhesion | 359/18862 | 8.88E-12 | 1.31E-09 |
| BP | negative regulation of locomotion | 377/18862 | 1.85E-11 | 2.49E-09 |
| BP | negative regulation of cell migration | 330/18862 | 3.21E-11 | 3.96E-09 |
| BP | skeletal system development | 486/18862 | 7.77E-10 | 8.83E-08 |
| BP | transmembrane receptor protein serine/threonine kinase signaling pathway | 358/18862 | 1.11E-08 | 1.17E-06 |
| BP | negative regulation of cell adhesion | 295/18862 | 1.22E-08 | 1.20E-06 |
| BP | connective tissue development | 243/18862 | 1.73E-08 | 1.60E-06 |
| BP | negative regulation of cell-substrate adhesion | 67/18862 | 2.62E-08 | 2.17E-06 |
| BP | negative regulation of angiogenesis | 146/18862 | 2.65E-08 | 2.17E-06 |
| BP | negative regulation of blood vessel morphogenesis | 148/18862 | 2.98E-08 | 2.32E-06 |
| BP | negative regulation of vasculature development | 149/18862 | 3.16E-08 | 2.33E-06 |
| BP | ossification | 401/18862 | 4.23E-08 | 2.98E-06 |
| BP | regulation of leukocyte chemotaxis | 119/18862 | 8.35E-08 | 5.48E-06 |
| BP | muscle contraction | 352/18862 | 8.52E-08 | 5.48E-06 |
| BP | regulation of peptidase activity | 455/18862 | 1.83E-07 | 1.13E-05 |
| BP | cartilage development | 185/18862 | 2.03E-07 | 1.20E-05 |
| BP | cellular response to transforming growth factor beta stimulus | 251/18862 | 2.64E-07 | 1.45E-05 |
| BP | regulation of transmembrane receptor protein serine/threonine kinase signaling pathway | 251/18862 | 2.64E-07 | 1.45E-05 |
| BP | response to ketone | 193/18862 | 2.90E-07 | 1.53E-05 |
| BP | response to transforming growth factor beta | 257/18862 | 3.29E-07 | 1.68E-05 |
| BP | regulation of leukocyte migration | 205/18862 | 4.83E-07 | 2.38E-05 |
| BP | blood coagulation | 342/18862 | 5.47E-07 | 2.61E-05 |
| BP | peptide cross-linking | 35/18862 | 5.73E-07 | 2.65E-05 |
| BP | hemostasis | 346/18862 | 6.13E-07 | 2.74E-05 |
| BP | coagulation | 347/18862 | 6.31E-07 | 2.74E-05 |
| BP | epithelial cell proliferation | 428/18862 | 6.91E-07 | 2.92E-05 |
| BP | monocyte chemotaxis | 68/18862 | 7.53E-07 | 3.06E-05 |
| BP | collagen metabolic process | 109/18862 | 7.65E-07 | 3.06E-05 |
| BP | osteoblast differentiation | 223/18862 | 9.77E-07 | 3.80E-05 |
| BP | regulation of cellular response to growth factor stimulus | 296/18862 | 1.19E-06 | 4.53E-05 |
| BP | muscle system process | 453/18862 | 1.25E-06 | 4.55E-05 |
| BP | cell-matrix adhesion | 230/18862 | 1.26E-06 | 4.55E-05 |
| BP | regulation of cell-matrix adhesion | 125/18862 | 1.93E-06 | 6.79E-05 |
| BP | extracellular matrix assembly | 45/18862 | 2.08E-06 | 7.15E-05 |
| BP | negative regulation of endopeptidase activity | 245/18862 | 2.13E-06 | 7.15E-05 |
| BP | positive regulation of extrinsic apoptotic signaling pathway | 48/18862 | 2.88E-06 | 9.47E-05 |
| BP | mononuclear cell migration | 190/18862 | 2.95E-06 | 9.50E-05 |
| BP | trabecula formation | 22/18862 | 3.03E-06 | 9.55E-05 |
| BP | negative regulation of peptidase activity | 257/18862 | 3.15E-06 | 9.55E-05 |
| BP | response to steroid hormone | 330/18862 | 3.16E-06 | 9.55E-05 |
| BP | positive regulation of receptor-mediated endocytosis | 50/18862 | 3.54E-06 | 0.000104673 |
| BP | positive regulation of leukocyte chemotaxis | 91/18862 | 4.21E-06 | 0.000119736 |
| BP | regulation of BMP signaling pathway | 91/18862 | 4.21E-06 | 0.000119736 |
| BP | negative regulation of proteolysis | 346/18862 | 4.81E-06 | 0.000134233 |
| BP | sulfur compound catabolic process | 56/18862 | 6.24E-06 | 0.000170933 |
| BP | smooth muscle contraction | 100/18862 | 7.28E-06 | 0.000195779 |
| BP | tissue migration | 365/18862 | 7.69E-06 | 0.000199765 |
| BP | regulation of extrinsic apoptotic signaling pathway | 154/18862 | 7.70E-06 | 0.000199765 |
| BP | regulation of extrinsic apoptotic signaling pathway via death domain receptors | 59/18862 | 8.09E-06 | 0.000204064 |
| BP | myeloid leukocyte migration | 218/18862 | 8.14E-06 | 0.000204064 |
| BP | negative regulation of epithelial cell proliferation | 160/18862 | 9.89E-06 | 0.000240608 |
| BP | regulation of chemotaxis | 224/18862 | 9.92E-06 | 0.000240608 |
| BP | glycosaminoglycan catabolic process | 62/18862 | 1.03E-05 | 0.000246725 |
| BP | leukocyte chemotaxis | 226/18862 | 1.06E-05 | 0.000248539 |
| BP | response to prostaglandin | 30/18862 | 1.10E-05 | 0.000251374 |
| BP | cellular response to vitamin | 30/18862 | 1.10E-05 | 0.000251374 |
| BP | elastic fiber assembly | 10/18862 | 1.15E-05 | 0.000257696 |
| BP | negative regulation of cellular response to growth factor stimulus | 110/18862 | 1.26E-05 | 0.000278218 |
| BP | cell chemotaxis | 306/18862 | 1.29E-05 | 0.000279854 |
| BP | regulation of mononuclear cell migration | 111/18862 | 1.33E-05 | 0.000284531 |
| BP | regulation of dendritic cell antigen processing and presentation | 11/18862 | 1.58E-05 | 0.000332957 |
| BP | aminoglycan catabolic process | 68/18862 | 1.63E-05 | 0.000339528 |
| BP | positive regulation of angiogenesis | 175/18862 | 1.77E-05 | 0.000353857 |
| BP | tissue remodeling | 175/18862 | 1.77E-05 | 0.000353857 |
| BP | positive regulation of vasculature development | 175/18862 | 1.77E-05 | 0.000353857 |
| BP | negative regulation of canonical Wnt signaling pathway | 179/18862 | 2.05E-05 | 0.000397065 |
| BP | dermatan sulfate biosynthetic process | 12/18862 | 2.09E-05 | 0.000397065 |
| BP | bone trabecula morphogenesis | 12/18862 | 2.09E-05 | 0.000397065 |
| BP | establishment of planar polarity involved in neural tube closure | 12/18862 | 2.09E-05 | 0.000397065 |
| BP | positive regulation of cell-substrate adhesion | 121/18862 | 2.17E-05 | 0.000406424 |
| BP | dermatan sulfate metabolic process | 13/18862 | 2.71E-05 | 0.000495389 |
| BP | positive regulation of extrinsic apoptotic signaling pathway via death domain receptors | 13/18862 | 2.71E-05 | 0.000495389 |
| BP | positive regulation of cell adhesion | 425/18862 | 2.86E-05 | 0.000515478 |
| BP | bone development | 193/18862 | 3.32E-05 | 0.000591835 |
| BP | chondroitin sulfate catabolic process | 14/18862 | 3.44E-05 | 0.000591835 |
| BP | establishment of planar polarity of embryonic epithelium | 14/18862 | 3.44E-05 | 0.000591835 |
| BP | regulation of fibrinolysis | 14/18862 | 3.44E-05 | 0.000591835 |
| BP | regulation of transforming growth factor beta production | 40/18862 | 3.56E-05 | 0.000604407 |
| BP | positive regulation of leukocyte migration | 133/18862 | 3.70E-05 | 0.00062251 |
| BP | negative regulation of cell-matrix adhesion | 41/18862 | 3.93E-05 | 0.000645126 |
| BP | prostate gland development | 41/18862 | 3.93E-05 | 0.000645126 |
| BP | dendritic cell antigen processing and presentation | 15/18862 | 4.29E-05 | 0.000680269 |
| BP | dermatan sulfate proteoglycan biosynthetic process | 15/18862 | 4.29E-05 | 0.000680269 |
| BP | cellular response to nutrient | 42/18862 | 4.32E-05 | 0.000680269 |
| BP | transforming growth factor beta production | 42/18862 | 4.32E-05 | 0.000680269 |
| BP | response to mechanical stimulus | 202/18862 | 4.44E-05 | 0.000691258 |
| BP | extrinsic apoptotic signaling pathway via death domain receptors | 84/18862 | 4.55E-05 | 0.000701403 |
| BP | endothelial cell migration | 278/18862 | 4.67E-05 | 0.000709632 |
| BP | positive regulation of chemotaxis | 139/18862 | 4.74E-05 | 0.000709632 |
| BP | trabecula morphogenesis | 43/18862 | 4.75E-05 | 0.000709632 |
| BP | regulation of Wnt signaling pathway | 365/18862 | 5.13E-05 | 0.000758593 |
| BP | dermatan sulfate proteoglycan metabolic process | 16/18862 | 5.26E-05 | 0.000770069 |
| BP | mesenchyme development | 287/18862 | 5.84E-05 | 0.000838991 |
| BP | regulation of canonical Wnt signaling pathway | 287/18862 | 5.84E-05 | 0.000838991 |
| BP | response to vitamin | 89/18862 | 6.01E-05 | 0.000849072 |
| BP | negative regulation of Wnt signaling pathway | 212/18862 | 6.03E-05 | 0.000849072 |
| BP | negative regulation of leukocyte migration | 46/18862 | 6.21E-05 | 0.000867084 |
| BP | cell growth | 470/18862 | 6.66E-05 | 0.000918389 |
| BP | response to fibroblast growth factor | 148/18862 | 6.73E-05 | 0.000918389 |
| BP | regulation of extracellular matrix organization | 47/18862 | 6.77E-05 | 0.000918389 |
| BP | extrinsic apoptotic signaling pathway | 217/18862 | 6.98E-05 | 0.000938584 |
| BP | regulation of cell adhesion mediated by integrin | 48/18862 | 7.36E-05 | 0.000980337 |
| BP | negative regulation of bone remodeling | 18/18862 | 7.61E-05 | 0.001005082 |
| BP | response to corticosteroid | 152/18862 | 7.80E-05 | 0.001021234 |
| BP | regulation of granulocyte chemotaxis | 49/18862 | 7.98E-05 | 0.001035701 |
| BP | BMP signaling pathway | 154/18862 | 8.39E-05 | 0.00107873 |
| BP | response to reactive oxygen species | 224/18862 | 8.52E-05 | 0.001086079 |
| BP | positive regulation of endocytosis | 97/18862 | 9.06E-05 | 0.001145645 |
| BP | platelet activation | 157/18862 | 9.33E-05 | 0.001169476 |
| BP | regulation of smooth muscle cell proliferation | 160/18862 | 0.000103562 | 0.001269978 |
| BP | cell-substrate junction assembly | 100/18862 | 0.000104725 | 0.001269978 |
| BP | regulation of epithelial to mesenchymal transition | 100/18862 | 0.000104725 | 0.001269978 |
| BP | regulation of antigen processing and presentation | 20/18862 | 0.000105621 | 0.001269978 |
| BP | negative regulation of tissue remodeling | 20/18862 | 0.000105621 | 0.001269978 |
| BP | smooth muscle cell proliferation | 162/18862 | 0.000110882 | 0.001322487 |
| BP | muscle organ development | 317/18862 | 0.000116677 | 0.001380469 |
| BP | response to nutrient | 165/18862 | 0.000122627 | 0.001439352 |
| BP | urogenital system development | 320/18862 | 0.000124497 | 0.001449804 |
| BP | response to BMP | 167/18862 | 0.00013099 | 0.001501766 |
| BP | cellular response to BMP stimulus | 167/18862 | 0.00013099 | 0.001501766 |
| BP | regulation of receptor-mediated endocytosis | 106/18862 | 0.000137911 | 0.001556967 |
| BP | cell-substrate junction organization | 106/18862 | 0.000137911 | 0.001556967 |
| BP | glycosaminoglycan biosynthetic process | 107/18862 | 0.000144144 | 0.001615009 |
| BP | response to prostaglandin E | 23/18862 | 0.00016243 | 0.001792034 |
| BP | negative regulation of mononuclear cell migration | 23/18862 | 0.00016243 | 0.001792034 |
| BP | canonical Wnt signaling pathway | 333/18862 | 0.000163579 | 0.001792034 |
| BP | regulation of endopeptidase activity | 426/18862 | 0.000165988 | 0.00180505 |
| BP | gland morphogenesis | 111/18862 | 0.00017126 | 0.001848791 |
| BP | positive regulation of neutrophil chemotaxis | 24/18862 | 0.000185009 | 0.001982741 |
| BP | regulation of protein processing | 61/18862 | 0.000188431 | 0.002004888 |
| BP | positive regulation of Wnt signaling pathway | 179/18862 | 0.000191157 | 0.002019365 |
| BP | aminoglycan biosynthetic process | 114/18862 | 0.00019403 | 0.002035178 |
| BP | mucopolysaccharide metabolic process | 115/18862 | 0.000202111 | 0.002093803 |
| BP | negative regulation of MAPK cascade | 181/18862 | 0.000203017 | 0.002093803 |
| BP | chondroitin sulfate biosynthetic process | 25/18862 | 0.000209529 | 0.002093803 |
| BP | fibrinolysis | 25/18862 | 0.000209529 | 0.002093803 |
| BP | prostate gland epithelium morphogenesis | 25/18862 | 0.000209529 | 0.002093803 |
| BP | regulation of metallopeptidase activity | 25/18862 | 0.000209529 | 0.002093803 |
| BP | regulation of non-canonical Wnt signaling pathway | 25/18862 | 0.000209529 | 0.002093803 |
| BP | regulation of protein maturation | 63/18862 | 0.000213562 | 0.002119775 |
| BP | regulation of focal adhesion assembly | 64/18862 | 0.000226993 | 0.002223245 |
| BP | regulation of cell-substrate junction assembly | 64/18862 | 0.000226993 | 0.002223245 |
| BP | regulation of blood coagulation | 66/18862 | 0.000255663 | 0.002446509 |
| BP | epithelial cell migration | 357/18862 | 0.000262246 | 0.002446509 |
| BP | axis elongation | 27/18862 | 0.000264676 | 0.002446509 |
| BP | negative regulation of protein processing | 27/18862 | 0.000264676 | 0.002446509 |
| BP | regulation of osteoblast proliferation | 27/18862 | 0.000264676 | 0.002446509 |
| BP | prostate gland morphogenesis | 27/18862 | 0.000264676 | 0.002446509 |
| BP | positive regulation of granulocyte chemotaxis | 27/18862 | 0.000264676 | 0.002446509 |
| BP | regulation of monocyte chemotaxis | 27/18862 | 0.000264676 | 0.002446509 |
| BP | negative regulation of protein maturation | 27/18862 | 0.000264676 | 0.002446509 |
| BP | regulation of hemostasis | 67/18862 | 0.000270933 | 0.002485845 |
| BP | response to drug | 359/18862 | 0.000272293 | 0.002485845 |
| BP | negative regulation of hydrolase activity | 456/18862 | 0.000274929 | 0.002494514 |
| BP | epithelium migration | 360/18862 | 0.000277434 | 0.002501888 |
| BP | positive regulation of vascular endothelial growth factor production | 28/18862 | 0.000295439 | 0.002648116 |
| BP | response to estradiol | 125/18862 | 0.00029779 | 0.002653107 |
| BP | regulation of cell-substrate junction organization | 69/18862 | 0.000303422 | 0.002687097 |
| BP | positive regulation of apoptotic signaling pathway | 126/18862 | 0.000308967 | 0.002719916 |
| BP | positive regulation of neutrophil migration | 29/18862 | 0.00032842 | 0.002873765 |
| BP | platelet degranulation | 128/18862 | 0.000332273 | 0.002873765 |
| BP | regulation of osteoblast differentiation | 128/18862 | 0.000332273 | 0.002873765 |
| BP | kidney development | 280/18862 | 0.000335634 | 0.00288596 |
| BP | regulation of coagulation | 71/18862 | 0.000338615 | 0.002894763 |
| BP | cell adhesion mediated by integrin | 72/18862 | 0.000357265 | 0.00302025 |
| BP | regulation of epithelial cell proliferation | 374/18862 | 0.000358158 | 0.00302025 |
| BP | ameboidal-type cell migration | 473/18862 | 0.00035942 | 0.00302025 |
| BP | chondroitin sulfate proteoglycan biosynthetic process | 30/18862 | 0.000363683 | 0.003038804 |
| BP | transforming growth factor beta receptor signaling pathway | 202/18862 | 0.000366072 | 0.003041578 |
| BP | regulation of wound healing | 131/18862 | 0.000369699 | 0.003054555 |
| BP | renal system development | 288/18862 | 0.000397571 | 0.003266593 |
| BP | regulation of endocytosis | 206/18862 | 0.000406314 | 0.00331998 |
| BP | negative regulation of transmembrane receptor protein serine/threonine kinase signaling pathway | 134/18862 | 0.00041023 | 0.003333567 |
| BP | negative regulation of endothelial cell proliferation | 75/18862 | 0.000417624 | 0.003375104 |
| BP | response to hydrogen peroxide | 135/18862 | 0.000424459 | 0.003393259 |
| BP | response to glucocorticoid | 135/18862 | 0.000424459 | 0.003393259 |
| BP | osteoblast proliferation | 32/18862 | 0.000441319 | 0.00349031 |
| BP | regulation of neutrophil chemotaxis | 32/18862 | 0.000441319 | 0.00349031 |
| BP | negative regulation of MAP kinase activity | 77/18862 | 0.000461698 | 0.003632061 |
| BP | skeletal system morphogenesis | 213/18862 | 0.000484987 | 0.003795084 |
| BP | morphogenesis of embryonic epithelium | 140/18862 | 0.000501271 | 0.00390186 |
| BP | cellular response to fibroblast growth factor stimulus | 142/18862 | 0.000534758 | 0.004140731 |
| BP | extracellular matrix disassembly | 81/18862 | 0.000559638 | 0.004310808 |
| BP | negative regulation of immune system process | 403/18862 | 0.000586752 | 0.004483423 |
| BP | response to calcium ion | 145/18862 | 0.00058811 | 0.004483423 |
| BP | muscle cell proliferation | 222/18862 | 0.000603054 | 0.004573772 |
| BP | regulation of cell growth | 406/18862 | 0.000615942 | 0.004647686 |
| BP | positive regulation of canonical Wnt signaling pathway | 147/18862 | 0.000625835 | 0.004698359 |
| BP | protein processing | 224/18862 | 0.000632087 | 0.004721334 |
| BP | developmental growth involved in morphogenesis | 225/18862 | 0.000647005 | 0.004808475 |
| BP | focal adhesion assembly | 85/18862 | 0.000671511 | 0.004965647 |
| BP | regulation of tissue remodeling | 86/18862 | 0.000701781 | 0.005163664 |
| BP | proteoglycan metabolic process | 87/18862 | 0.000733004 | 0.005359117 |
| BP | chondroitin sulfate metabolic process | 38/18862 | 0.000735591 | 0.005359117 |
| BP | mesenchymal cell differentiation | 231/18862 | 0.000742341 | 0.005381781 |
| BP | epithelial to mesenchymal transition | 153/18862 | 0.000749946 | 0.005410395 |
| BP | response to alcohol | 233/18862 | 0.000776423 | 0.005574219 |
| BP | bone remodeling | 89/18862 | 0.000798378 | 0.005704152 |
| BP | positive regulation of epithelial cell apoptotic process | 40/18862 | 0.000855727 | 0.006074325 |
| BP | receptor-mediated endocytosis | 328/18862 | 0.000858404 | 0.006074325 |
| BP | glycosaminoglycan metabolic process | 158/18862 | 0.000866714 | 0.006103924 |
| BP | biomineral tissue development | 160/18862 | 0.000917026 | 0.006427647 |
| BP | response to cAMP | 93/18862 | 0.000941307 | 0.006501461 |
| BP | negative regulation of fibrinolysis | 10/18862 | 0.000945141 | 0.006501461 |
| BP | planar cell polarity pathway involved in neural tube closure | 10/18862 | 0.000945141 | 0.006501461 |
| BP | regulation of dopaminergic neuron differentiation | 10/18862 | 0.000945141 | 0.006501461 |
| BP | biomineralization | 162/18862 | 0.000969483 | 0.00663803 |
| BP | regulation of epithelial cell apoptotic process | 94/18862 | 0.000979674 | 0.006676897 |
| BP | regulation of neutrophil migration | 42/18862 | 0.000987643 | 0.00670033 |
| BP | positive regulation of cytokine production | 437/18862 | 0.000992273 | 0.006700999 |
| BP | regulation of response to wounding | 164/18862 | 0.001024141 | 0.006884777 |
| BP | chondroitin sulfate proteoglycan metabolic process | 43/18862 | 0.001058154 | 0.007081237 |
| BP | response to oxidative stress | 444/18862 | 0.001098606 | 0.007271717 |
| BP | response to chemokine | 97/18862 | 0.001101368 | 0.007271717 |
| BP | cellular response to chemokine | 97/18862 | 0.001101368 | 0.007271717 |
| BP | skin morphogenesis | 11/18862 | 0.001151671 | 0.00747044 |
| BP | regulation of establishment of planar polarity involved in neural tube closure | 11/18862 | 0.001151671 | 0.00747044 |
| BP | dendritic cell apoptotic process | 11/18862 | 0.001151671 | 0.00747044 |
| BP | regulation of dendritic cell apoptotic process | 11/18862 | 0.001151671 | 0.00747044 |
| BP | response to progesterone | 45/18862 | 0.001208543 | 0.007780335 |
| BP | regulation of apoptotic signaling pathway | 348/18862 | 0.001209967 | 0.007780335 |
| BP | aminoglycan metabolic process | 172/18862 | 0.001265934 | 0.00810498 |
| BP | muscle cell migration | 101/18862 | 0.001279631 | 0.008157355 |
| BP | morphogenesis of a branching epithelium | 173/18862 | 0.001298884 | 0.008244554 |
| BP | regulation of collagen metabolic process | 47/18862 | 0.001371763 | 0.008351304 |
| BP | collagen catabolic process | 47/18862 | 0.001371763 | 0.008351304 |
| BP | chondrocyte differentiation | 103/18862 | 0.001375922 | 0.008351304 |
| BP | response to retinoic acid | 103/18862 | 0.001375922 | 0.008351304 |
| BP | striated muscle cell development | 103/18862 | 0.001375922 | 0.008351304 |
| BP | establishment of T cell polarity | 12/18862 | 0.001377817 | 0.008351304 |
| BP | regulation of transforming growth factor beta1 production | 12/18862 | 0.001377817 | 0.008351304 |
| BP | negative regulation of cell adhesion mediated by integrin | 12/18862 | 0.001377817 | 0.008351304 |
| BP | keratan sulfate catabolic process | 12/18862 | 0.001377817 | 0.008351304 |
| BP | behavioral response to pain | 12/18862 | 0.001377817 | 0.008351304 |
| BP | cellular response to X-ray | 12/18862 | 0.001377817 | 0.008351304 |
| BP | stress-activated MAPK cascade | 261/18862 | 0.001392777 | 0.008407529 |
| BP | regulation of animal organ morphogenesis | 176/18862 | 0.001401543 | 0.008426049 |
| BP | regulation of peptidyl-tyrosine phosphorylation | 262/18862 | 0.001420163 | 0.008503426 |
| BP | regulation of endothelial cell proliferation | 177/18862 | 0.001437057 | 0.008569886 |
| BP | negative regulation of blood coagulation | 48/18862 | 0.001458309 | 0.008627047 |
| BP | regulation of morphogenesis of a branching structure | 48/18862 | 0.001458309 | 0.008627047 |
| BP | post-translational protein modification | 361/18862 | 0.001493232 | 0.008798454 |
| BP | gastrulation | 179/18862 | 0.001510072 | 0.008862371 |
| BP | cellular response to interleukin-1 | 180/18862 | 0.001547588 | 0.008944218 |
| BP | negative regulation of stress-activated MAPK cascade | 49/18862 | 0.001548209 | 0.008944218 |
| BP | negative regulation of stress-activated protein kinase signaling cascade | 49/18862 | 0.001548209 | 0.008944218 |
| BP | negative regulation of hemostasis | 49/18862 | 0.001548209 | 0.008944218 |
| BP | regulation of stress-activated MAPK cascade | 181/18862 | 0.001585786 | 0.009125659 |
| BP | establishment of lymphocyte polarity | 13/18862 | 0.001623396 | 0.00912896 |
| BP | immunological synapse formation | 13/18862 | 0.001623396 | 0.00912896 |
| BP | regulation of plasminogen activation | 13/18862 | 0.001623396 | 0.00912896 |
| BP | transforming growth factor beta1 production | 13/18862 | 0.001623396 | 0.00912896 |
| BP | negative regulation of osteoblast proliferation | 13/18862 | 0.001623396 | 0.00912896 |
| BP | negative regulation of extracellular matrix organization | 13/18862 | 0.001623396 | 0.00912896 |
| BP | substrate adhesion-dependent cell spreading | 108/18862 | 0.001638604 | 0.009179581 |
| BP | peptidyl-tyrosine phosphorylation | 369/18862 | 0.001691755 | 0.009441573 |
| BP | regulation of stress-activated protein kinase signaling cascade | 184/18862 | 0.001704549 | 0.00947721 |
| BP | negative regulation of BMP signaling pathway | 51/18862 | 0.001738263 | 0.009592535 |
| BP | regulation of bone remodeling | 51/18862 | 0.001738263 | 0.009592535 |
| BP | negative regulation of cell growth | 185/18862 | 0.001745551 | 0.009596943 |
| BP | peptidyl-tyrosine modification | 372/18862 | 0.001771288 | 0.009702378 |
| BP | morphogenesis of a branching structure | 186/18862 | 0.001787273 | 0.009753808 |
| BP | negative regulation of coagulation | 52/18862 | 0.001838508 | 0.009996531 |
| BP | stress-activated protein kinase signaling cascade | 276/18862 | 0.001848436 | 0.0100137 |
| BP | cardiac left ventricle morphogenesis | 14/18862 | 0.001888226 | 0.010081539 |
| BP | negative regulation of cell morphogenesis involved in differentiation | 14/18862 | 0.001888226 | 0.010081539 |
| BP | negative regulation of substrate adhesion-dependent cell spreading | 14/18862 | 0.001888226 | 0.010081539 |
| BP | negative regulation of metallopeptidase activity | 14/18862 | 0.001888226 | 0.010081539 |
| BP | bone mineralization | 113/18862 | 0.001934303 | 0.010259097 |
| BP | endoderm formation | 53/18862 | 0.001942292 | 0.010259097 |
| BP | muscle fiber development | 53/18862 | 0.001942292 | 0.010259097 |
| BP | embryonic epithelial tube formation | 114/18862 | 0.001997585 | 0.010513606 |
| BP | endothelial cell proliferation | 191/18862 | 0.002006946 | 0.010525418 |
| BP | reactive oxygen species metabolic process | 281/18862 | 0.002023003 | 0.010572136 |
| BP | regulation of reactive oxygen species metabolic process | 192/18862 | 0.002053147 | 0.010691889 |
| BP | formation of primary germ layer | 115/18862 | 0.002062287 | 0.010701803 |
| BP | sulfur compound biosynthetic process | 193/18862 | 0.002100121 | 0.010860031 |
| BP | epithelial cell apoptotic process | 116/18862 | 0.002128424 | 0.010968039 |
| BP | response to oxygen levels | 385/18862 | 0.002150163 | 0.011041588 |
| BP | negative regulation of JUN kinase activity | 15/18862 | 0.002172126 | 0.011077448 |
| BP | regulation of Wnt signaling pathway, planar cell polarity pathway | 15/18862 | 0.002172126 | 0.011077448 |
| BP | regulation of cell junction assembly | 195/18862 | 0.00219642 | 0.011162851 |
| BP | carbohydrate derivative catabolic process | 198/18862 | 0.002346859 | 0.01188658 |
| BP | T cell costimulation | 57/18862 | 0.002393679 | 0.01204124 |
| BP | negative regulation of reproductive process | 57/18862 | 0.002393679 | 0.01204124 |
| BP | negative regulation of response to external stimulus | 394/18862 | 0.002447253 | 0.012247463 |
| BP | positive regulation of peptidase activity | 200/18862 | 0.002451236 | 0.012247463 |
| BP | convergent extension | 16/18862 | 0.002474917 | 0.012282792 |
| BP | midbrain dopaminergic neuron differentiation | 16/18862 | 0.002474917 | 0.012282792 |
| BP | protein maturation | 293/18862 | 0.002492837 | 0.012320304 |
| BP | regulation of vascular endothelial growth factor production | 58/18862 | 0.002515797 | 0.012320304 |
| BP | zymogen activation | 58/18862 | 0.002515797 | 0.012320304 |
| BP | negative regulation of peptidyl-tyrosine phosphorylation | 58/18862 | 0.002515797 | 0.012320304 |
| BP | cellular response to tumor necrosis factor | 296/18862 | 0.002622168 | 0.01276779 |
| BP | odontogenesis | 123/18862 | 0.002632932 | 0.01276779 |
| BP | lymphocyte costimulation | 59/18862 | 0.002641706 | 0.01276779 |
| BP | positive regulation of wound healing | 59/18862 | 0.002641706 | 0.01276779 |
| BP | granulocyte chemotaxis | 124/18862 | 0.002711133 | 0.01306066 |
| BP | response to interleukin-1 | 206/18862 | 0.002784649 | 0.013255635 |
| BP | epithelial tube formation | 125/18862 | 0.002790913 | 0.013255635 |
| BP | regulation of extracellular matrix disassembly | 17/18862 | 0.00279642 | 0.013255635 |
| BP | mitotic cell cycle arrest | 17/18862 | 0.00279642 | 0.013255635 |
| BP | epithelial cell-cell adhesion | 17/18862 | 0.00279642 | 0.013255635 |
| BP | regulation of ERK1 and ERK2 cascade | 301/18862 | 0.002848859 | 0.01343463 |
| BP | reproductive structure development | 405/18862 | 0.002852349 | 0.01343463 |
| BP | regulation of morphogenesis of an epithelium | 61/18862 | 0.002905047 | 0.013639402 |
| BP | reproductive system development | 408/18862 | 0.002971298 | 0.01390631 |
| BP | aging | 304/18862 | 0.002991753 | 0.013957872 |
| BP | vascular endothelial growth factor production | 62/18862 | 0.003042555 | 0.014105892 |
| BP | proteoglycan biosynthetic process | 62/18862 | 0.003042555 | 0.014105892 |
| BP | regulation of JNK cascade | 129/18862 | 0.003126142 | 0.014288698 |
| BP | negative regulation of nitric oxide biosynthetic process | 18/18862 | 0.003136458 | 0.014288698 |
| BP | negative regulation of focal adhesion assembly | 18/18862 | 0.003136458 | 0.014288698 |
| BP | negative regulation of cell-substrate junction organization | 18/18862 | 0.003136458 | 0.014288698 |
| BP | negative regulation of nitric oxide metabolic process | 18/18862 | 0.003136458 | 0.014288698 |
| BP | regulation of MAP kinase activity | 307/18862 | 0.003139954 | 0.014288698 |
| BP | negative regulation of smooth muscle cell proliferation | 63/18862 | 0.003184005 | 0.014400538 |
| BP | negative regulation of reactive oxygen species metabolic process | 63/18862 | 0.003184005 | 0.014400538 |
| BP | response to organophosphorus | 130/18862 | 0.003214055 | 0.01449213 |
| BP | negative regulation of chemotaxis | 64/18862 | 0.003329432 | 0.014921378 |
| BP | cellular response to retinoic acid | 64/18862 | 0.003329432 | 0.014921378 |
| BP | visual perception | 215/18862 | 0.003344771 | 0.014944835 |
| BP | cellular response to ionizing radiation | 65/18862 | 0.003478871 | 0.015497191 |
| BP | negative regulation of leukocyte chemotaxis | 19/18862 | 0.003494856 | 0.015521644 |
| BP | sensory perception of light stimulus | 219/18862 | 0.003618103 | 0.016020908 |
| BP | cell junction assembly | 425/18862 | 0.003718975 | 0.016418414 |
| BP | positive regulation of osteoblast differentiation | 67/18862 | 0.003789924 | 0.01668184 |
| BP | response to tumor necrosis factor | 320/18862 | 0.003846457 | 0.016830498 |
| BP | ERK1 and ERK2 cascade | 320/18862 | 0.003846457 | 0.016830498 |
| BP | type B pancreatic cell proliferation | 20/18862 | 0.003871437 | 0.016840153 |
| BP | cellular response to prostaglandin stimulus | 20/18862 | 0.003871437 | 0.016840153 |
| BP | tube formation | 140/18862 | 0.004187718 | 0.018162507 |
| BP | positive regulation of cell adhesion mediated by integrin | 21/18862 | 0.00426603 | 0.018340795 |
| BP | peptidyl-tyrosine autophosphorylation | 21/18862 | 0.00426603 | 0.018340795 |
| BP | positive regulation of transforming growth factor beta production | 21/18862 | 0.00426603 | 0.018340795 |
| BP | cellular response to abiotic stimulus | 330/18862 | 0.00446556 | 0.019087653 |
| BP | cellular response to environmental stimulus | 330/18862 | 0.00446556 | 0.019087653 |
| BP | response to purine-containing compound | 144/18862 | 0.004627407 | 0.019663217 |
| BP | negative regulation of cysteine-type endopeptidase activity involved in apoptotic process | 72/18862 | 0.004640099 | 0.019663217 |
| BP | positive regulation of response to wounding | 72/18862 | 0.004640099 | 0.019663217 |
| BP | response to hyperoxia | 22/18862 | 0.004678461 | 0.019712812 |
| BP | cellular response to vitamin D | 22/18862 | 0.004678461 | 0.019712812 |
| BP | regulation of DNA-binding transcription factor activity | 444/18862 | 0.004715893 | 0.019814083 |
| BP | negative regulation of protein serine/threonine kinase activity | 145/18862 | 0.004741997 | 0.019867322 |
| BP | nitric oxide biosynthetic process | 73/18862 | 0.004822819 | 0.020148857 |
| BP | cellular response to extracellular stimulus | 235/18862 | 0.004873461 | 0.020303078 |
| BP | negative regulation of wound healing | 74/18862 | 0.005009836 | 0.020812594 |
| BP | granulocyte migration | 148/18862 | 0.005097199 | 0.02081347 |
| BP | regulation of platelet-derived growth factor receptor signaling pathway | 23/18862 | 0.005108559 | 0.02081347 |
| BP | positive regulation of blood coagulation | 23/18862 | 0.005108559 | 0.02081347 |
| BP | plasminogen activation | 23/18862 | 0.005108559 | 0.02081347 |
| BP | negative regulation of chondrocyte differentiation | 23/18862 | 0.005108559 | 0.02081347 |
| BP | regulation of membrane protein ectodomain proteolysis | 23/18862 | 0.005108559 | 0.02081347 |
| BP | positive regulation of hemostasis | 23/18862 | 0.005108559 | 0.02081347 |
| BP | endoderm development | 76/18862 | 0.005396873 | 0.021927723 |
| BP | dendritic cell chemotaxis | 24/18862 | 0.005556154 | 0.022268997 |
| BP | positive regulation of glycoprotein biosynthetic process | 24/18862 | 0.005556154 | 0.022268997 |
| BP | negative regulation of fibroblast growth factor receptor signaling pathway | 24/18862 | 0.005556154 | 0.022268997 |
| BP | regulation of insulin-like growth factor receptor signaling pathway | 24/18862 | 0.005556154 | 0.022268997 |
| BP | positive regulation of coagulation | 24/18862 | 0.005556154 | 0.022268997 |
| BP | nitric oxide metabolic process | 77/18862 | 0.005596948 | 0.022311566 |
| BP | response to axon injury | 77/18862 | 0.005596948 | 0.022311566 |
| BP | non-canonical Wnt signaling pathway | 153/18862 | 0.005728148 | 0.022773196 |
| BP | response to hypoxia | 348/18862 | 0.005761514 | 0.022819167 |
| BP | negative regulation of growth | 245/18862 | 0.005799673 | 0.022819167 |
| BP | negative regulation of ERK1 and ERK2 cascade | 78/18862 | 0.005801428 | 0.022819167 |
| BP | reactive nitrogen species metabolic process | 78/18862 | 0.005801428 | 0.022819167 |
| BP | positive regulation of receptor internalization | 25/18862 | 0.006021078 | 0.023557825 |
| BP | negative regulation of chemokine production | 25/18862 | 0.006021078 | 0.023557825 |
| BP | response to metal ion | 352/18862 | 0.006083374 | 0.023738758 |
| BP | regulation of epithelial cell differentiation | 156/18862 | 0.00613069 | 0.023860443 |
| BP | negative regulation of cysteine-type endopeptidase activity | 80/18862 | 0.006223708 | 0.02415889 |
| BP | regulation of smooth muscle cell migration | 81/18862 | 0.006441557 | 0.024939068 |
| BP | inactivation of MAPK activity | 26/18862 | 0.006503163 | 0.025111843 |
| BP | regulation of reproductive process | 159/18862 | 0.006551657 | 0.025233218 |
| BP | neural tube closure | 82/18862 | 0.00666391 | 0.02558648 |
| BP | eye development | 359/18862 | 0.00667798 | 0.02558648 |
| BP | response to decreased oxygen levels | 360/18862 | 0.006766255 | 0.025857714 |
| BP | regulation of muscle contraction | 161/18862 | 0.006842712 | 0.025997747 |
| BP | positive regulation of protein binding | 83/18862 | 0.006890791 | 0.025997747 |
| BP | regulation of JUN kinase activity | 83/18862 | 0.006890791 | 0.025997747 |
| BP | regulation of megakaryocyte differentiation | 83/18862 | 0.006890791 | 0.025997747 |
| BP | tube closure | 83/18862 | 0.006890791 | 0.025997747 |
| BP | negative regulation of protein phosphorylation | 362/18862 | 0.006945349 | 0.026136911 |
| BP | response to pain | 27/18862 | 0.007002242 | 0.026217588 |
| BP | positive regulation of glycoprotein metabolic process | 27/18862 | 0.007002242 | 0.026217588 |
| BP | visual system development | 363/18862 | 0.007036178 | 0.026278123 |
| BP | lung development | 163/18862 | 0.007142215 | 0.026606953 |
| BP | positive regulation of proteolysis | 367/18862 | 0.00740814 | 0.027528266 |
| BP | keratan sulfate biosynthetic process | 28/18862 | 0.00751815 | 0.027659074 |
| BP | negative regulation of cartilage development | 28/18862 | 0.00751815 | 0.027659074 |
| BP | protein activation cascade | 28/18862 | 0.00751815 | 0.027659074 |
| BP | blood coagulation, fibrin clot formation | 28/18862 | 0.00751815 | 0.027659074 |
| BP | sensory system development | 369/18862 | 0.007599375 | 0.027888525 |
| BP | respiratory tube development | 167/18862 | 0.007766973 | 0.02843303 |
| BP | muscle tissue development | 371/18862 | 0.00779416 | 0.028462106 |
| BP | positive regulation of tumor necrosis factor production | 87/18862 | 0.007844024 | 0.028503437 |
| BP | membrane depolarization | 87/18862 | 0.007844024 | 0.028503437 |
| BP | muscle cell development | 168/18862 | 0.007928599 | 0.02874015 |
| BP | chondrocyte development | 29/18862 | 0.008050722 | 0.028775001 |
| BP | response to increased oxygen levels | 29/18862 | 0.008050722 | 0.028775001 |
| BP | dendritic cell migration | 29/18862 | 0.008050722 | 0.028775001 |
| BP | face morphogenesis | 29/18862 | 0.008050722 | 0.028775001 |
| BP | regulation of execution phase of apoptosis | 29/18862 | 0.008050722 | 0.028775001 |
| BP | production of molecular mediator involved in inflammatory response | 88/18862 | 0.008093865 | 0.028775001 |
| BP | primary neural tube formation | 88/18862 | 0.008093865 | 0.028775001 |
| BP | smooth muscle cell migration | 88/18862 | 0.008093865 | 0.028775001 |
| BP | regulation of chemokine production | 89/18862 | 0.008348359 | 0.029608593 |
| BP | striated muscle cell differentiation | 269/18862 | 0.008517996 | 0.030094741 |
| BP | negative regulation of cardiac muscle cell apoptotic process | 30/18862 | 0.008599797 | 0.030094741 |
| BP | positive regulation of filopodium assembly | 30/18862 | 0.008599797 | 0.030094741 |
| BP | bone morphogenesis | 90/18862 | 0.008607524 | 0.030094741 |
| BP | negative regulation of response to wounding | 90/18862 | 0.008607524 | 0.030094741 |
| BP | positive regulation of tumor necrosis factor superfamily cytokine production | 90/18862 | 0.008607524 | 0.030094741 |
| BP | cellular response to ketone | 92/18862 | 0.009139943 | 0.031880882 |
| BP | negative regulation of DNA-binding transcription factor activity | 177/18862 | 0.009483622 | 0.03300183 |
| BP | complement activation | 178/18862 | 0.009667769 | 0.033445497 |
| BP | execution phase of apoptosis | 94/18862 | 0.00969126 | 0.033445497 |
| BP | response to X-ray | 32/18862 | 0.009746803 | 0.033445497 |
| BP | negative regulation of striated muscle cell apoptotic process | 32/18862 | 0.009746803 | 0.033445497 |
| BP | embryonic eye morphogenesis | 32/18862 | 0.009746803 | 0.033445497 |
| BP | negative regulation of cell junction assembly | 32/18862 | 0.009746803 | 0.033445497 |
| BP | chemokine production | 95/18862 | 0.009974047 | 0.034146042 |
| BP | neural tube formation | 96/18862 | 0.010261607 | 0.034968609 |
| BP | positive regulation of smooth muscle cell proliferation | 96/18862 | 0.010261607 | 0.034968609 |
| BP | keratan sulfate metabolic process | 33/18862 | 0.010344415 | 0.0350088 |
| BP | positive regulation of phosphatidylinositol 3-kinase activity | 33/18862 | 0.010344415 | 0.0350088 |
| BP | head morphogenesis | 33/18862 | 0.010344415 | 0.0350088 |
| BP | negative regulation of endothelial cell migration | 97/18862 | 0.010553955 | 0.035636401 |
| BP | response to vitamin D | 34/18862 | 0.010957888 | 0.036916034 |
| BP | regulation of plasma membrane bounded cell projection assembly | 186/18862 | 0.011225182 | 0.037730576 |
| BP | megakaryocyte differentiation | 100/18862 | 0.01145987 | 0.038432072 |
| BP | regulation of fibroblast growth factor receptor signaling pathway | 35/18862 | 0.011587063 | 0.038592972 |
| BP | insulin-like growth factor receptor signaling pathway | 35/18862 | 0.011587063 | 0.038592972 |
| BP | negative regulation of reactive oxygen species biosynthetic process | 35/18862 | 0.011587063 | 0.038592972 |
| BP | regulation of cell projection assembly | 188/18862 | 0.011638322 | 0.038592972 |
| BP | respiratory system development | 188/18862 | 0.011638322 | 0.038592972 |
| BP | regulation of receptor signaling pathway via JAK-STAT | 101/18862 | 0.01177151 | 0.0389473 |
| BP | JNK cascade | 189/18862 | 0.011848516 | 0.03911458 |
| BP | negative regulation of phosphorylation | 407/18862 | 0.01194843 | 0.039356569 |
| BP | leukocyte apoptotic process | 102/18862 | 0.012088005 | 0.039727829 |
| BP | actomyosin structure organization | 191/18862 | 0.012276203 | 0.040167828 |
| BP | cellular response to radiation | 191/18862 | 0.012276203 | 0.040167828 |
| BP | neutrophil chemotaxis | 103/18862 | 0.012409367 | 0.040424672 |
| BP | negative regulation of extrinsic apoptotic signaling pathway | 103/18862 | 0.012409367 | 0.040424672 |
| BP | regeneration | 192/18862 | 0.012493715 | 0.040609993 |
| BP | gland development | 413/18862 | 0.012768331 | 0.041411599 |
| BP | negative regulation of JNK cascade | 37/18862 | 0.012891898 | 0.041448781 |
| BP | dopaminergic neuron differentiation | 37/18862 | 0.012891898 | 0.041448781 |
| BP | positive regulation of lipid kinase activity | 37/18862 | 0.012891898 | 0.041448781 |
| BP | regulation of leukocyte adhesion to vascular endothelial cell | 37/18862 | 0.012891898 | 0.041448781 |
| BP | response to monosaccharide | 196/18862 | 0.01338844 | 0.042719808 |
| BP | regulation of protein binding | 196/18862 | 0.01338844 | 0.042719808 |
| BP | integrin-mediated signaling pathway | 106/18862 | 0.01340277 | 0.042719808 |
| BP | positive regulation of plasma membrane bounded cell projection assembly | 106/18862 | 0.01340277 | 0.042719808 |
| BP | detection of biotic stimulus | 38/18862 | 0.013567249 | 0.04305847 |
| BP | negative regulation of ossification | 38/18862 | 0.013567249 | 0.04305847 |
| BP | cellular response to external stimulus | 303/18862 | 0.013724533 | 0.043432103 |
| BP | positive regulation of peptidyl-serine phosphorylation | 107/18862 | 0.01374371 | 0.043432103 |
| BP | glycoprotein metabolic process | 421/18862 | 0.013922197 | 0.043902338 |
| BP | Wnt signaling pathway, planar cell polarity pathway | 108/18862 | 0.014089571 | 0.044335603 |
| BP | regulation of receptor signaling pathway via STAT | 110/18862 | 0.014796085 | 0.04645277 |
| BP | epithelial tube morphogenesis | 309/18862 | 0.014825211 | 0.04645277 |
| BP | negative regulation of epithelial to mesenchymal transition | 40/18862 | 0.014963049 | 0.046747984 |
| BP | regulation of neuron projection development | 428/18862 | 0.014990214 | 0.046747984 |
| BP | camera-type eye development | 310/18862 | 0.015014254 | 0.046747984 |
| BP | receptor internalization | 111/18862 | 0.015156756 | 0.04709253 |
| BP | regulation of ossification | 112/18862 | 0.01552238 | 0.048026742 |
| BP | regulation of establishment of planar polarity | 112/18862 | 0.01552238 | 0.048026742 |
| BP | response to testosterone | 41/18862 | 0.015683196 | 0.048322128 |
| BP | negative regulation of extrinsic apoptotic signaling pathway via death domain receptors | 41/18862 | 0.015683196 | 0.048322128 |
| CC | collagen-containing extracellular matrix | 423/19520 | 5.28E-51 | 4.90E-49 |
| CC | collagen trimer | 87/19520 | 1.34E-16 | 6.21E-15 |
| CC | endoplasmic reticulum lumen | 306/19520 | 2.72E-15 | 8.39E-14 |
| CC | basement membrane | 94/19520 | 4.83E-10 | 1.12E-08 |
| CC | complex of collagen trimers | 21/19520 | 3.57E-08 | 6.62E-07 |
| CC | fibrillar collagen trimer | 12/19520 | 2.03E-07 | 2.69E-06 |
| CC | banded collagen fibril | 12/19520 | 2.03E-07 | 2.69E-06 |
| CC | lysosomal lumen | 96/19520 | 5.39E-06 | 6.24E-05 |
| CC | contractile fiber | 231/19520 | 1.15E-05 | 0.000118044 |
| CC | sarcolemma | 134/19520 | 3.63E-05 | 0.000336312 |
| CC | platelet alpha granule | 91/19520 | 6.34E-05 | 0.000533787 |
| CC | Golgi lumen | 102/19520 | 0.000109104 | 0.000842204 |
| CC | vacuolar lumen | 173/19520 | 0.000149486 | 0.001065164 |
| CC | Z disc | 122/19520 | 0.000252609 | 0.001602195 |
| CC | platelet alpha granule lumen | 67/19520 | 0.000259446 | 0.001602195 |
| CC | lamellipodium | 196/19520 | 0.000293563 | 0.001699573 |
| CC | sarcomere | 203/19520 | 0.000354124 | 0.001856511 |
| CC | actin filament bundle | 73/19520 | 0.000360754 | 0.001856511 |
| CC | I band | 134/19520 | 0.00038962 | 0.001899533 |
| CC | sarcoplasm | 79/19520 | 0.000487635 | 0.002258521 |
| CC | myofibril | 224/19520 | 0.000596066 | 0.002629262 |
| CC | secretory granule lumen | 322/19520 | 0.000721792 | 0.003039123 |
| CC | cytoplasmic vesicle lumen | 326/19520 | 0.000776004 | 0.003104294 |
| CC | vesicle lumen | 328/19520 | 0.000804294 | 0.003104294 |
| CC | microfibril | 12/19520 | 0.001346435 | 0.004988897 |
| CC | muscle myosin complex | 15/19520 | 0.00212287 | 0.007563263 |
| CC | dynactin complex | 16/19520 | 0.002418877 | 0.008298683 |
| CC | stress fiber | 64/19520 | 0.003223177 | 0.010295447 |
| CC | contractile actin filament bundle | 64/19520 | 0.003223177 | 0.010295447 |
| CC | myosin II complex | 19/19520 | 0.00341607 | 0.010547864 |
| CC | neuromuscular junction | 69/19520 | 0.003986756 | 0.011912887 |
| CC | lamellipodium membrane | 22/19520 | 0.004573457 | 0.0127134 |
| CC | myosin filament | 22/19520 | 0.004573457 | 0.0127134 |
| CC | blood microparticle | 146/19520 | 0.004666396 | 0.0127134 |
| CC | actomyosin | 74/19520 | 0.004851723 | 0.012840649 |
| CC | filamentous actin | 32/19520 | 0.009531263 | 0.024524888 |
| CC | cell leading edge | 411/19520 | 0.011862893 | 0.029326088 |
| CC | filopodium | 103/19520 | 0.012030361 | 0.029326088 |
| CC | focal adhesion | 416/19520 | 0.01253203 | 0.029765686 |
| CC | cell-substrate junction | 423/19520 | 0.013512972 | 0.031293198 |
| CC | actin filament | 113/19520 | 0.015413109 | 0.034822942 |
| MF | extracellular matrix structural constituent | 170/18337 | 1.08E-43 | 1.33E-41 |
| MF | collagen binding | 68/18337 | 5.25E-18 | 3.24E-16 |
| MF | glycosaminoglycan binding | 228/18337 | 1.65E-13 | 6.77E-12 |
| MF | extracellular matrix structural constituent conferring tensile strength | 41/18337 | 2.21E-13 | 6.82E-12 |
| MF | extracellular matrix structural constituent conferring compression resistance | 22/18337 | 6.26E-10 | 1.54E-08 |
| MF | heparin binding | 164/18337 | 4.91E-09 | 1.01E-07 |
| MF | sulfur compound binding | 260/18337 | 3.61E-08 | 6.35E-07 |
| MF | peptidase regulator activity | 230/18337 | 1.22E-07 | 1.88E-06 |
| MF | protease binding | 131/18337 | 1.82E-07 | 2.24E-06 |
| MF | fibronectin binding | 28/18337 | 1.82E-07 | 2.24E-06 |
| MF | extracellular matrix binding | 55/18337 | 2.15E-07 | 2.41E-06 |
| MF | growth factor binding | 137/18337 | 3.66E-06 | 3.76E-05 |
| MF | integrin binding | 142/18337 | 4.64E-06 | 4.40E-05 |
| MF | Wnt-protein binding | 30/18337 | 1.13E-05 | 9.90E-05 |
| MF | platelet-derived growth factor binding | 11/18337 | 1.60E-05 | 0.000131319 |
| MF | proteoglycan binding | 36/18337 | 2.37E-05 | 0.000182332 |
| MF | endopeptidase regulator activity | 192/18337 | 3.31E-05 | 0.00023953 |
| MF | peptidase activator activity | 43/18337 | 4.84E-05 | 0.00033117 |
| MF | metalloendopeptidase inhibitor activity | 16/18337 | 5.34E-05 | 0.000345938 |
| MF | endopeptidase inhibitor activity | 180/18337 | 0.000201995 | 0.001243863 |
| MF | peptidase inhibitor activity | 187/18337 | 0.00024821 | 0.001455666 |
| MF | actin binding | 439/18337 | 0.001051749 | 0.005887779 |
| MF | serine-type endopeptidase inhibitor activity | 98/18337 | 0.001164896 | 0.006237661 |
| MF | receptor ligand activity | 486/18337 | 0.001999483 | 0.010260507 |
| MF | signaling receptor activator activity | 492/18337 | 0.002157856 | 0.010630279 |
| MF | structural constituent of eye lens | 23/18337 | 0.005159445 | 0.024439479 |
| MF | transforming growth factor beta binding | 24/18337 | 0.00561142 | 0.025595949 |
| MF | growth factor activity | 162/18337 | 0.007111196 | 0.031278569 |
| MF | enzyme inhibitor activity | 385/18337 | 0.009466752 | 0.040203629 |

**Table 7b. KEGG enrichment analysis.**

| ID | Description | BgRatio | pvalue | qvalue |
| --- | --- | --- | --- | --- |
| hsa04510 | Focal adhesion | 201/8098 | 1.10E-10 | 1.11E-08 |
| hsa04512 | ECM-receptor interaction | 88/8098 | 2.01E-09 | 1.01E-07 |
| hsa04974 | Protein digestion and absorption | 103/8098 | 8.24E-09 | 2.78E-07 |
| hsa05205 | Proteoglycans in cancer | 205/8098 | 3.06E-06 | 7.73E-05 |
| hsa04933 | AGE-RAGE signaling pathway in diabetic complications | 100/8098 | 2.78E-05 | 0.000560948 |
| hsa04151 | PI3K-Akt signaling pathway | 354/8098 | 4.01E-05 | 0.00067522 |
| hsa05165 | Human papillomavirus infection | 331/8098 | 0.000138021 | 0.001992485 |
| hsa05144 | Malaria | 50/8098 | 0.000222085 | 0.002805286 |
| hsa05146 | Amoebiasis | 102/8098 | 0.000352966 | 0.003963128 |
| hsa04270 | Vascular smooth muscle contraction | 134/8098 | 0.001224138 | 0.01237024 |
| hsa04610 | Complement and coagulation cascades | 85/8098 | 0.001672009 | 0.015360082 |

**Appendix 8**

**gene set enrichment analyses (GSEA)**

**Table 8a. GSEA of high rish.**

| NAME | SIZE | ES | NES | NOM p-val | FDR q-val |
| --- | --- | --- | --- | --- | --- |
| KEGG_FOCAL_ADHESION | 199 | 0.7061547 | 2.4490328 | 0 | 0 |
| KEGG_ECM_RECEPTOR_INTERACTION | 84 | 0.80678 | 2.3951943 | 0 | 0 |
| KEGG_CYTOKINE_CYTOKINE_RECEPTOR_INTERACTION | 264 | 0.6230982 | 2.3471797 | 0 | 0 |
| KEGG_HYPERTROPHIC_CARDIOMYOPATHY_HCM | 83 | 0.66134614 | 2.251496 | 0 | 2.37E-04 |
| KEGG_DILATED_CARDIOMYOPATHY | 90 | 0.6594351 | 2.2321093 | 0 | 3.93E-04 |
| KEGG_HEMATOPOIETIC_CELL_LINEAGE | 85 | 0.70773923 | 2.216785 | 0 | 4.71E-04 |
| KEGG_HEDGEHOG_SIGNALING_PATHWAY | 56 | 0.65542346 | 2.1968927 | 0 | 5.29E-04 |
| KEGG_CELL_ADHESION_MOLECULES_CAMS | 131 | 0.66586655 | 2.183457 | 0 | 5.48E-04 |
| KEGG_AXON_GUIDANCE | 129 | 0.583707 | 2.1769176 | 0 | 4.87E-04 |
| KEGG_REGULATION_OF_ACTIN_CYTOSKELETON | 213 | 0.5433051 | 2.1474996 | 0 | 6.70E-04 |
| KEGG_GAP_JUNCTION | 90 | 0.5800237 | 2.1457765 | 0 | 6.09E-04 |
| KEGG_NEUROACTIVE_LIGAND_RECEPTOR_INTERACTION | 270 | 0.5310138 | 2.1283348 | 0 | 0.001024115 |
| KEGG_ARRHYTHMOGENIC_RIGHT_VENTRICULAR_CARDIOMYOPATHY_ARVC | 74 | 0.62648726 | 2.1210926 | 0.001960784 | 0.001229865 |
| KEGG_COMPLEMENT_AND_COAGULATION_CASCADES | 69 | 0.7129374 | 2.1206038 | 0 | 0.001281881 |
| KEGG_JAK_STAT_SIGNALING_PATHWAY | 155 | 0.5488147 | 2.116113 | 0.002024292 | 0.001339544 |
| KEGG_GLYCOSPHINGOLIPID_BIOSYNTHESIS_GANGLIO_SERIES | 15 | 0.80045176 | 2.0998213 | 0 | 0.001625066 |
| KEGG_MAPK_SIGNALING_PATHWAY | 267 | 0.5178693 | 2.0830264 | 0 | 0.002041171 |
| KEGG_MELANOMA | 71 | 0.5803696 | 2.0763352 | 0 | 0.002013029 |
| KEGG_GLYCOSAMINOGLYCAN_BIOSYNTHESIS_CHONDROITIN_SULFATE | 22 | 0.8028214 | 2.065554 | 0 | 0.002316919 |
| KEGG_PATHWAYS_IN_CANCER | 325 | 0.5199089 | 2.0295463 | 0 | 0.00438782 |
| KEGG_LEUKOCYTE_TRANSENDOTHELIAL_MIGRATION | 116 | 0.5610776 | 2.0208325 | 0 | 0.004646785 |
| KEGG_CALCIUM_SIGNALING_PATHWAY | 178 | 0.51228416 | 2.0098903 | 0 | 0.004793943 |
| KEGG_TGF_BETA_SIGNALING_PATHWAY | 86 | 0.5520385 | 1.955871 | 0 | 0.00932048 |
| KEGG_VASCULAR_SMOOTH_MUSCLE_CONTRACTION | 115 | 0.54259557 | 1.9235748 | 0.002053388 | 0.012654777 |
| KEGG_PRION_DISEASES | 35 | 0.6120119 | 1.9167709 | 0.002 | 0.013094145 |
| KEGG_RENAL_CELL_CARCINOMA | 70 | 0.5580325 | 1.9080502 | 0.002008032 | 0.013665187 |
| KEGG_CHEMOKINE_SIGNALING_PATHWAY | 188 | 0.5365064 | 1.8918451 | 0.006198347 | 0.015757311 |
| KEGG_LEISHMANIA_INFECTION | 70 | 0.6470737 | 1.8806663 | 0.01183432 | 0.017253848 |
| KEGG_BLADDER_CANCER | 42 | 0.58172935 | 1.8728518 | 0.001930502 | 0.017770903 |
| KEGG_GLYCOSAMINOGLYCAN_BIOSYNTHESIS_HEPARAN_SULFATE | 26 | 0.6250282 | 1.8616705 | 0 | 0.018672291 |
| KEGG_TOLL_LIKE_RECEPTOR_SIGNALING_PATHWAY | 102 | 0.51953495 | 1.846702 | 0.00203252 | 0.020791959 |
| KEGG_BASAL_CELL_CARCINOMA | 55 | 0.5564341 | 1.831107 | 0.005905512 | 0.023000833 |
| KEGG_VIRAL_MYOCARDITIS | 68 | 0.553187 | 1.8228316 | 0.021868788 | 0.02408626 |
| KEGG_DORSO_VENTRAL_AXIS_FORMATION | 24 | 0.5742867 | 1.7859985 | 0.01629328 | 0.0317038 |
| KEGG_MELANOGENESIS | 101 | 0.47316417 | 1.7848837 | 0.003968254 | 0.031062586 |
| KEGG_GLYCOSAMINOGLYCAN_BIOSYNTHESIS_KERATAN_SULFATE | 15 | 0.67925686 | 1.769739 | 0.007936508 | 0.034122072 |
| KEGG_NATURAL_KILLER_CELL_MEDIATED_CYTOTOXICITY | 132 | 0.48915642 | 1.758336 | 0.015936255 | 0.036804706 |
| KEGG_ASTHMA | 28 | 0.6815653 | 1.7572609 | 0.01996008 | 0.036134984 |
| KEGG_GLYCOSAMINOGLYCAN_DEGRADATION | 21 | 0.6247602 | 1.7452478 | 0.006085193 | 0.038327422 |
| KEGG_ADIPOCYTOKINE_SIGNALING_PATHWAY | 66 | 0.48775923 | 1.7386612 | 0.011605416 | 0.03946906 |
| KEGG_PROSTATE_CANCER | 89 | 0.4900992 | 1.7337586 | 0.022680413 | 0.039963394 |
| KEGG_MTOR_SIGNALING_PATHWAY | 52 | 0.51102924 | 1.7010332 | 0.023206752 | 0.050004583 |
| KEGG_SMALL_CELL_LUNG_CANCER | 84 | 0.49353468 | 1.6967748 | 0.022312373 | 0.049709942 |
| KEGG_LONG_TERM_DEPRESSION | 70 | 0.45096594 | 1.6904734 | 0.007736944 | 0.050606295 |
| KEGG_ACUTE_MYELOID_LEUKEMIA | 57 | 0.5272058 | 1.6842268 | 0.020491803 | 0.051528532 |
| KEGG_NEUROTROPHIN_SIGNALING_PATHWAY | 126 | 0.46907726 | 1.6682804 | 0.028688524 | 0.05642186 |
| KEGG_NOD_LIKE_RECEPTOR_SIGNALING_PATHWAY | 62 | 0.51137686 | 1.6623105 | 0.045454547 | 0.057439785 |
| KEGG_GRAFT_VERSUS_HOST_DISEASE | 37 | 0.68452084 | 1.6601276 | 0.06883365 | 0.05677476 |
| KEGG_PANCREATIC_CANCER | 70 | 0.494446 | 1.6560514 | 0.03245436 | 0.05689256 |
| KEGG_VEGF_SIGNALING_PATHWAY | 76 | 0.44303307 | 1.6116436 | 0.02 | 0.07330488 |
| KEGG_ALDOSTERONE_REGULATED_SODIUM_REABSORPTION | 42 | 0.46520865 | 1.5984914 | 0.026369167 | 0.07767257 |
| KEGG_ERBB_SIGNALING_PATHWAY | 87 | 0.45655686 | 1.597842 | 0.0375 | 0.076531745 |
| KEGG_WNT_SIGNALING_PATHWAY | 151 | 0.41240948 | 1.5921495 | 0.043650795 | 0.077037625 |
| KEGG_AUTOIMMUNE_THYROID_DISEASE | 50 | 0.56005734 | 1.5900565 | 0.045908183 | 0.076687 |
| KEGG_T_CELL_RECEPTOR_SIGNALING_PATHWAY | 108 | 0.4692321 | 1.5627654 | 0.08097166 | 0.087619536 |
| KEGG_CHRONIC_MYELOID_LEUKEMIA | 73 | 0.48074296 | 1.5473862 | 0.058091287 | 0.093991466 |
| KEGG_GLIOMA | 65 | 0.45807475 | 1.5452774 | 0.04918033 | 0.09328835 |
| KEGG_ENDOCYTOSIS | 181 | 0.39857268 | 1.5095693 | 0.0499002 | 0.109961644 |
| KEGG_ABC_TRANSPORTERS | 44 | 0.4331225 | 1.4886916 | 0.04752066 | 0.12057222 |
| KEGG_INTESTINAL_IMMUNE_NETWORK_FOR_IGA_PRODUCTION | 46 | 0.55130273 | 1.4847925 | 0.1515748 | 0.12068375 |
| KEGG_APOPTOSIS | 87 | 0.43093166 | 1.4767017 | 0.10612245 | 0.12348268 |
| KEGG_TIGHT_JUNCTION | 132 | 0.36458787 | 1.4745233 | 0.039447732 | 0.12317874 |
| KEGG_ALLOGRAFT_REJECTION | 35 | 0.59855783 | 1.4669682 | 0.16216215 | 0.12589563 |
| KEGG_FC_GAMMA_R_MEDIATED_PHAGOCYTOSIS | 96 | 0.423076 | 1.4624156 | 0.08349515 | 0.12679094 |
| KEGG_NOTCH_SIGNALING_PATHWAY | 47 | 0.4549506 | 1.4537147 | 0.096 | 0.1302423 |
| KEGG_LYSOSOME | 121 | 0.45470297 | 1.4534953 | 0.12301587 | 0.12837374 |
| KEGG_EPITHELIAL_CELL_SIGNALING_IN_HELICOBACTER_PYLORI_INFECTION | 68 | 0.40859097 | 1.4466448 | 0.07602339 | 0.13040745 |
| KEGG_ADHERENS_JUNCTION | 73 | 0.41446573 | 1.4376445 | 0.08016032 | 0.13360828 |
| KEGG_TYPE_I_DIABETES_MELLITUS | 41 | 0.53015244 | 1.4279134 | 0.1743295 | 0.13837577 |
| KEGG_TYPE_II_DIABETES_MELLITUS | 47 | 0.42481756 | 1.4205761 | 0.083333336 | 0.14134994 |
| KEGG_COLORECTAL_CANCER | 62 | 0.4265769 | 1.40995 | 0.12916666 | 0.14677247 |
| KEGG_O_GLYCAN_BIOSYNTHESIS | 30 | 0.44906634 | 1.3822508 | 0.13279678 | 0.16368712 |
| KEGG_INSULIN_SIGNALING_PATHWAY | 136 | 0.35864228 | 1.3670248 | 0.10123967 | 0.17359415 |
| KEGG_GNRH_SIGNALING_PATHWAY | 101 | 0.35592335 | 1.365398 | 0.08943089 | 0.1723815 |
| KEGG_B_CELL_RECEPTOR_SIGNALING_PATHWAY | 75 | 0.43434787 | 1.3512684 | 0.188 | 0.18032105 |
| KEGG_ARACHIDONIC_ACID_METABOLISM | 58 | 0.36610505 | 1.325268 | 0.10958904 | 0.19821085 |
| KEGG_VASOPRESSIN_REGULATED_WATER_REABSORPTION | 44 | 0.37518072 | 1.3202157 | 0.14052953 | 0.19937798 |
| KEGG_PATHOGENIC_ESCHERICHIA_COLI_INFECTION | 56 | 0.37840828 | 1.3054018 | 0.13671875 | 0.20891136 |
| KEGG_FC_EPSILON_RI_SIGNALING_PATHWAY | 79 | 0.36127728 | 1.3027216 | 0.16221766 | 0.2080669 |
| KEGG_ANTIGEN_PROCESSING_AND_PRESENTATION | 81 | 0.42736682 | 1.3016549 | 0.22626263 | 0.20625153 |
| KEGG_REGULATION_OF_AUTOPHAGY | 35 | 0.39665356 | 1.2839727 | 0.18164062 | 0.21826167 |
| KEGG_RENIN_ANGIOTENSIN_SYSTEM | 17 | 0.44192877 | 1.2590301 | 0.20039682 | 0.23681772 |
| KEGG_PROGESTERONE_MEDIATED_OOCYTE_MATURATION | 85 | 0.34241772 | 1.2168362 | 0.222 | 0.27132887 |
| KEGG_OLFACTORY_TRANSDUCTION | 386 | 0.43780488 | 1.1966333 | 0.35412475 | 0.28854063 |
| KEGG_AMYOTROPHIC_LATERAL_SCLEROSIS_ALS | 53 | 0.32325855 | 1.1858255 | 0.21774194 | 0.29533827 |
| KEGG_PPAR_SIGNALING_PATHWAY | 69 | 0.34201014 | 1.1738602 | 0.24375 | 0.30441576 |
| KEGG_ETHER_LIPID_METABOLISM | 33 | 0.35038888 | 1.1697649 | 0.2208589 | 0.3050761 |
| KEGG_PRIMARY_BILE_ACID_BIOSYNTHESIS | 16 | 0.3989548 | 1.1244172 | 0.32238194 | 0.34973982 |
| KEGG_SYSTEMIC_LUPUS_ERYTHEMATOSUS | 135 | 0.3350144 | 1.1205229 | 0.32239383 | 0.3500025 |
| KEGG_PHOSPHATIDYLINOSITOL_SIGNALING_SYSTEM | 76 | 0.32361177 | 1.108825 | 0.3292683 | 0.3599605 |
| KEGG_RIG_I_LIKE_RECEPTOR_SIGNALING_PATHWAY | 71 | 0.30653107 | 1.1022737 | 0.33669356 | 0.36333472 |
| KEGG_GLYCEROLIPID_METABOLISM | 49 | 0.31384495 | 1.0849398 | 0.3188119 | 0.3784565 |
| KEGG_LONG_TERM_POTENTIATION | 70 | 0.28365967 | 1.0711746 | 0.33677685 | 0.39056945 |
| KEGG_SPHINGOLIPID_METABOLISM | 39 | 0.33527508 | 1.0700191 | 0.3783231 | 0.3880202 |
| KEGG_NICOTINATE_AND_NICOTINAMIDE_METABOLISM | 24 | 0.33305287 | 1.0428032 | 0.39105058 | 0.41563568 |
| KEGG_GLYCEROPHOSPHOLIPID_METABOLISM | 77 | 0.26541564 | 1.0101999 | 0.44970414 | 0.4510274 |
| KEGG_INOSITOL_PHOSPHATE_METABOLISM | 54 | 0.2941569 | 0.97693074 | 0.44624746 | 0.48915118 |
| KEGG_ENDOMETRIAL_CANCER | 52 | 0.2854182 | 0.9145393 | 0.5397149 | 0.5701573 |
| KEGG_STEROID_HORMONE_BIOSYNTHESIS | 53 | 0.26967752 | 0.9042215 | 0.58158994 | 0.57992625 |
| KEGG_GALACTOSE_METABOLISM | 25 | 0.29608357 | 0.8761149 | 0.627451 | 0.6157598 |
| KEGG_GLYCOSPHINGOLIPID_BIOSYNTHESIS_LACTO_AND_NEOLACTO_SERIES | 26 | 0.2678135 | 0.8751968 | 0.60940695 | 0.61071867 |
| KEGG_PHENYLALANINE_METABOLISM | 18 | 0.29803023 | 0.871911 | 0.6347656 | 0.6097594 |
| KEGG_CYTOSOLIC_DNA_SENSING_PATHWAY | 55 | 0.24919268 | 0.84516346 | 0.65360826 | 0.6425793 |
| KEGG_NON_SMALL_CELL_LUNG_CANCER | 54 | 0.2512241 | 0.8301071 | 0.61538464 | 0.6586101 |
| KEGG_OTHER_GLYCAN_DEGRADATION | 16 | 0.30926952 | 0.80232674 | 0.6720648 | 0.6918142 |
| KEGG_TASTE_TRANSDUCTION | 51 | 0.2367763 | 0.7666605 | 0.756238 | 0.737771 |
| KEGG_SNARE_INTERACTIONS_IN_VESICULAR_TRANSPORT | 38 | 0.22625887 | 0.7252052 | 0.8148148 | 0.7886365 |
| KEGG_VIBRIO_CHOLERAE_INFECTION | 54 | 0.19324216 | 0.7048532 | 0.8792079 | 0.80837953 |
| KEGG_STARCH_AND_SUCROSE_METABOLISM | 49 | 0.20288041 | 0.6944857 | 0.8730159 | 0.8150044 |
| KEGG_PRIMARY_IMMUNODEFICIENCY | 35 | 0.251122 | 0.6385775 | 0.77649325 | 0.8714427 |

**Table 8b. GSEA of low rish.**

| NAME | SIZE | ES | NES | NOM p-val | FDR q-val |
| --- | --- | --- | --- | --- | --- |
| KEGG_SPLICEOSOME | 127 | -0.7165126 | -2.0490358 | 0 | 0.028701525 |
| KEGG_RNA_DEGRADATION | 59 | -0.6769281 | -2.045521 | 0.003929273 | 0.01497992 |
| KEGG_HUNTINGTONS_DISEASE | 182 | -0.5533627 | -2.0019102 | 0.00589391 | 0.01911673 |
| KEGG_BASE_EXCISION_REPAIR | 35 | -0.73359126 | -1.9941732 | 0 | 0.01661119 |
| KEGG_OXIDATIVE_PHOSPHORYLATION | 132 | -0.6683302 | -1.9850789 | 0.009861933 | 0.015123852 |
| KEGG_PEROXISOME | 78 | -0.59177583 | -1.9445782 | 0 | 0.020308543 |
| KEGG_DNA_REPLICATION | 36 | -0.8058326 | -1.9437612 | 0.00204499 | 0.017614892 |
| KEGG_PARKINSONS_DISEASE | 130 | -0.6436859 | -1.9406464 | 0.015841585 | 0.015872749 |
| KEGG_HOMOLOGOUS_RECOMBINATION | 28 | -0.7278022 | -1.9395673 | 0.006024096 | 0.01434214 |
| KEGG_NUCLEOTIDE_EXCISION_REPAIR | 44 | -0.6683529 | -1.9106421 | 0.012448133 | 0.017912766 |
| KEGG_CITRATE_CYCLE_TCA_CYCLE | 31 | -0.74940526 | -1.8972495 | 0.002040816 | 0.018980432 |
| KEGG_MISMATCH_REPAIR | 23 | -0.775163 | -1.8947821 | 0.00203666 | 0.017618475 |
| KEGG_PROPANOATE_METABOLISM | 33 | -0.62867224 | -1.8392985 | 0.021611001 | 0.029330565 |
| KEGG_ALZHEIMERS_DISEASE | 166 | -0.5121526 | -1.832968 | 0.01775148 | 0.02931397 |
| KEGG_PYRIMIDINE_METABOLISM | 98 | -0.5459749 | -1.7955981 | 0.020325202 | 0.039442077 |
| KEGG_AMINOACYL_TRNA_BIOSYNTHESIS | 41 | -0.6938084 | -1.7899411 | 0.016032064 | 0.038207605 |
| KEGG_VALINE_LEUCINE_AND_ISOLEUCINE_DEGRADATION | 44 | -0.632431 | -1.7770942 | 0.013944224 | 0.039679743 |
| KEGG_RNA_POLYMERASE | 29 | -0.64199984 | -1.7660762 | 0.026748972 | 0.041519444 |
| KEGG_RIBOSOME | 88 | -0.7748569 | -1.7630869 | 0.044806518 | 0.040501885 |
| KEGG_PROTEASOME | 46 | -0.67918044 | -1.7613875 | 0.027139874 | 0.03903632 |
| KEGG_GLYOXYLATE_AND_DICARBOXYLATE_METABOLISM | 16 | -0.7206906 | -1.7594451 | 0.005988024 | 0.03778781 |
| KEGG_BUTANOATE_METABOLISM | 34 | -0.57726306 | -1.7455356 | 0.010080645 | 0.040511765 |
| KEGG_ONE_CARBON_POOL_BY_FOLATE | 17 | -0.6979182 | -1.7277964 | 0.018 | 0.04496913 |
| KEGG_PYRUVATE_METABOLISM | 40 | -0.5230633 | -1.694932 | 0.023809524 | 0.055000763 |
| KEGG_TERPENOID_BACKBONE_BIOSYNTHESIS | 15 | -0.69421214 | -1.6879195 | 0.030800821 | 0.055258494 |
| KEGG_GLYCOSYLPHOSPHATIDYLINOSITOL_GPI_ANCHOR_BIOSYNTHESIS | 25 | -0.5905111 | -1.6719267 | 0.02972399 | 0.059373856 |
| KEGG_CELL_CYCLE | 125 | -0.53258497 | -1.6391736 | 0.048484847 | 0.07013358 |
| KEGG_BASAL_TRANSCRIPTION_FACTORS | 35 | -0.4913711 | -1.4882816 | 0.07024793 | 0.15822075 |
| KEGG_RIBOFLAVIN_METABOLISM | 16 | -0.51364404 | -1.4503937 | 0.06944445 | 0.18389456 |
| KEGG_GLUTATHIONE_METABOLISM | 49 | -0.42500523 | -1.4158864 | 0.108510636 | 0.20919906 |
| KEGG_LYSINE_DEGRADATION | 44 | -0.45242986 | -1.3658887 | 0.16767676 | 0.25267228 |
| KEGG_CARDIAC_MUSCLE_CONTRACTION | 79 | -0.36745784 | -1.364227 | 0.11561866 | 0.24636297 |
| KEGG_FATTY_ACID_METABOLISM | 42 | -0.462859 | -1.3583394 | 0.15810277 | 0.24494503 |
| KEGG_SELENOAMINO_ACID_METABOLISM | 26 | -0.4590369 | -1.3468702 | 0.13360325 | 0.2504591 |
| KEGG_PURINE_METABOLISM | 159 | -0.33313492 | -1.344284 | 0.11683168 | 0.24595791 |
| KEGG_MATURITY_ONSET_DIABETES_OF_THE_YOUNG | 25 | -0.4982229 | -1.3435539 | 0.15820312 | 0.23992582 |
| KEGG_PROTEIN_EXPORT | 24 | -0.48561105 | -1.3001158 | 0.20990099 | 0.27854094 |
| KEGG_BIOSYNTHESIS_OF_UNSATURATED_FATTY_ACIDS | 22 | -0.4475931 | -1.2951314 | 0.17303823 | 0.276816 |
| KEGG_STEROID_BIOSYNTHESIS | 17 | -0.52615994 | -1.2915467 | 0.20824742 | 0.27295917 |
| KEGG_DRUG_METABOLISM_OTHER_ENZYMES | 49 | -0.37092873 | -1.2815989 | 0.16895874 | 0.27683517 |
| KEGG_ARGININE_AND_PROLINE_METABOLISM | 54 | -0.36995444 | -1.278977 | 0.16293278 | 0.27249843 |
| KEGG_NITROGEN_METABOLISM | 23 | -0.42021942 | -1.2693206 | 0.18019801 | 0.27575633 |
| KEGG_OOCYTE_MEIOSIS | 113 | -0.3372458 | -1.2589709 | 0.19329388 | 0.2802408 |
| KEGG_PENTOSE_PHOSPHATE_PATHWAY | 27 | -0.4084568 | -1.2419878 | 0.2314225 | 0.2913925 |
| KEGG_CYSTEINE_AND_METHIONINE_METABOLISM | 34 | -0.38129932 | -1.2140723 | 0.21696253 | 0.315363 |
| KEGG_HISTIDINE_METABOLISM | 29 | -0.3612682 | -1.2102879 | 0.22334003 | 0.31282088 |
| KEGG_BETA_ALANINE_METABOLISM | 22 | -0.4103538 | -1.2012608 | 0.23780487 | 0.31626552 |
| KEGG_N_GLYCAN_BIOSYNTHESIS | 46 | -0.40167263 | -1.1769862 | 0.2965932 | 0.33519387 |
| KEGG_UBIQUITIN_MEDIATED_PROTEOLYSIS | 135 | -0.31470233 | -1.0833796 | 0.3647541 | 0.44307354 |
| KEGG_PROXIMAL_TUBULE_BICARBONATE_RECLAMATION | 23 | -0.34098876 | -1.0702064 | 0.36234817 | 0.4524975 |
| KEGG_TRYPTOPHAN_METABOLISM | 40 | -0.3059986 | -1.0690228 | 0.36883628 | 0.44518602 |
| KEGG_ALANINE_ASPARTATE_AND_GLUTAMATE_METABOLISM | 32 | -0.33708906 | -1.0681201 | 0.36491936 | 0.43774235 |
| KEGG_LINOLEIC_ACID_METABOLISM | 29 | -0.329717 | -1.0489124 | 0.3809524 | 0.45585495 |
| KEGG_TYROSINE_METABOLISM | 42 | -0.2919847 | -1.0455055 | 0.38675213 | 0.45185795 |
| KEGG_P53_SIGNALING_PATHWAY | 68 | -0.30844876 | -1.0375959 | 0.412 | 0.45375463 |
| KEGG_GLYCOLYSIS_GLUCONEOGENESIS | 61 | -0.30223894 | -1.0313691 | 0.39553753 | 0.453334 |
| KEGG_FRUCTOSE_AND_MANNOSE_METABOLISM | 34 | -0.3020182 | -0.92025197 | 0.54247105 | 0.60287595 |
| KEGG_GLYCINE_SERINE_AND_THREONINE_METABOLISM | 31 | -0.29029322 | -0.91105384 | 0.56573707 | 0.60620135 |
| KEGG_PORPHYRIN_AND_CHLOROPHYLL_METABOLISM | 39 | -0.28859907 | -0.9094372 | 0.576 | 0.59804225 |
| KEGG_ALPHA_LINOLENIC_ACID_METABOLISM | 19 | -0.3058923 | -0.90858024 | 0.60348165 | 0.5894904 |
| KEGG_PANTOTHENATE_AND_COA_BIOSYNTHESIS | 16 | -0.3190038 | -0.8920226 | 0.5946502 | 0.60322034 |
| KEGG_PENTOSE_AND_GLUCURONATE_INTERCONVERSIONS | 26 | -0.30651078 | -0.8795498 | 0.5804598 | 0.6117345 |
| KEGG_AMINO_SUGAR_AND_NUCLEOTIDE_SUGAR_METABOLISM | 44 | -0.26082727 | -0.7946886 | 0.6967871 | 0.72997844 |
| KEGG_THYROID_CANCER | 29 | -0.22201212 | -0.67454076 | 0.86885244 | 0.8885997 |
| KEGG_METABOLISM_OF_XENOBIOTICS_BY_CYTOCHROME_P450 | 67 | -0.20255029 | -0.665002 | 0.87525153 | 0.88629144 |
| KEGG_DRUG_METABOLISM_CYTOCHROME_P450 | 69 | -0.19239278 | -0.6380425 | 0.9233871 | 0.9035821 |
| KEGG_ASCORBATE_AND_ALDARATE_METABOLISM | 23 | -0.2165328 | -0.5888997 | 0.92514396 | 0.9343543 |
| KEGG_RETINOL_METABOLISM | 62 | -0.17515075 | -0.584889 | 0.9728682 | 0.9233217 |
